# Synthesis, Characterization, and Computational Studies on Gallium(III) and Iron(III) Complexes with a Pentadentate Macrocyclic *bis*-Phosphinate Chelator and Their Investigation As Molecular Scaffolds for ^18^F Binding

**DOI:** 10.1021/acs.inorgchem.3c03135

**Published:** 2023-12-06

**Authors:** Danielle E. Runacres, Victoria K. Greenacre, John M. Dyke, Julian Grigg, George Herbert, William Levason, Graeme McRobbie, Gillian Reid

**Affiliations:** † School of Chemistry, University of Southampton, Southampton SO17 1BJ, United Kingdom; ‡ GE HealthCare, Pollards Wood, Nightingales Lane, Chalfont St. Giles, Buckinghamshire HP8 4SP, United Kingdom

## Abstract

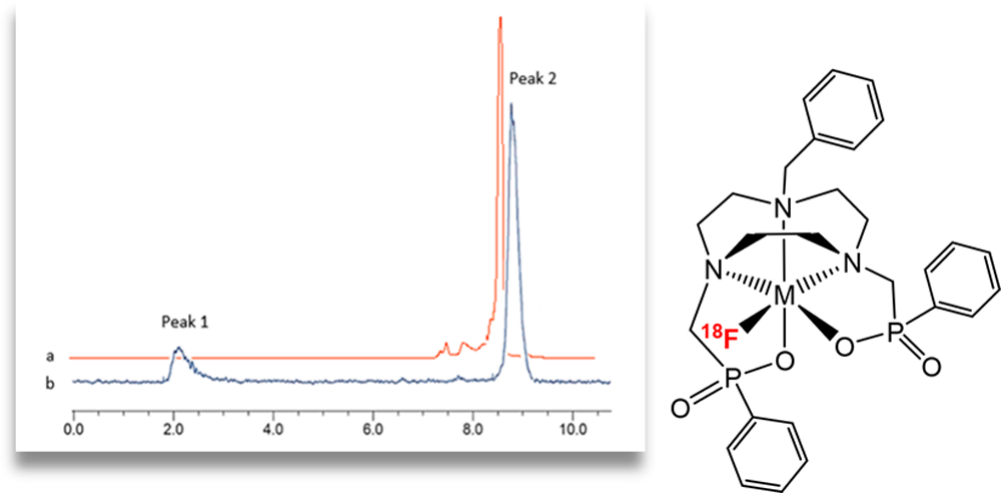

With the aim of obtaining improved molecular scaffolds for ^18^F binding to use in PET imaging, gallium(III) and iron(III) complexes with a macrocyclic *bis*-phosphinate chelator have been synthesized and their properties, including their fluoride binding ability, investigated. Reaction of Bn-tacn (1-benzyl-1,4,7-triazacyclononane) with paraformaldehyde and PhP(OR)_2_ (R = Me or Et) in refluxing THF, followed by acid hydrolysis, yields the macrocyclic *bis*(phosphinic acid) derivative, H_2_(Bn-NODP) (1-benzyl-4,7-phenylphosphinic acid-1,4,7-triazacyclononane), which is isolated as its protonated form, H_2_(Bn-NODP)·2HCl·4H_2_O, at low pH (HCl_aq_), its disodium salt, Na_2_(Bn-NODP)·5H_2_O at pH 12 (NaOH_aq_), or the neutral H_2_(Bn-NODP) under mildly basic conditions (Et_3_N). A crystal structure of H_2_(Bn-NODP)·2HCl·H_2_O confirmed the ligand’s identity. The mononuclear [GaCl(Bn-NODP)] complex was prepared by treatment of either the HCl or sodium salt with Ga(NO_3_)_3_·9H_2_O or GaCl_3_, while treatment of H_2_(Bn-NODP)·2HCl·4H_2_O with FeCl_3_ in aqueous HCl gives [FeCl(Bn-NODP)]. The addition of 1 mol. equiv of aqueous KF to these chloro complexes readily forms the [MF(Bn-NODP)] analogues. Spectroscopic analysis on these complexes confirms pentadentate coordination of the doubly deprotonated (*bis*-phosphinate) macrocycle via its N_3_O_2_ donor set, with the halide ligand completing a distorted octahedral geometry; this is further confirmed through a crystal structure analysis on [GaF(Bn-NODP)]·4H_2_O. The complex adopts the geometric isomer in which the phosphinate arms are coordinated unsymmetrically (isomer 1) and with the stereochemistry of the three N atoms of the tacn ring in the *RRS* configuration, denoted (N)*RRS,* and the phosphinate groups in the *RR* stereochemistry, denoted (P)*RR,* (isomer 1/*RR*), together with its (N)*SSR* (P)*SS* enantiomer. The greater thermodynamic stability of isomer 1/*RR* over the other possible isomers is also indicated by density functional theory (DFT) calculations. Radiofluorination experiments on the [MCl(Bn-NODP)] complexes in partially aqueous MeCN/NaOAc_aq_ (Ga) or EtOH (Ga or Fe; i.e. without buffer) with ^18^F^–^ target water at 80 °C/10 min lead to high radiochemical incorporation (radiochemical yields 60–80% at 1 mg/mL, or ∼1.5 μM, concentration of the precursor). While the [Fe^18^F(n-NODP)] is unstable (loss of ^18^F^–^) in both H_2_O/EtOH and PBS/EtOH (PBS = phosphate buffered saline), the [Ga^18^F(Bn-NODP)] radioproduct shows excellent stability, RCP = 99% at *t* = 4 h (RCP = radiochemical purity) when formulated in 90%:10% H_2_O/EtOH and *ca*. 95% RCP over 4 h when formulated in 90%:10% PBS/EtOH. This indicates that the new “Ga^III^(Bn-NODP)” moiety is a considerably superior fluoride binding scaffold than the previously reported [Ga^18^F(Bn-NODA)] (Bn-NODA = 1-benzyl-4,7-dicarboxylate-1,4,7-triazacyclononane), which undergoes rapid and complete hydrolysis in PBS/EtOH (refer to *Chem. Eur. J.***2015**, *21*, 4688–4694).

## Abstract 2

The preparation and characterization of a *bis*(phosphinate)-functionalized macrocyclic chelator, H_2_(Bn-NODP), is reported, together with the distorted octahedral [MX(Bn-NODP)] (M = Ga, Fe) complexes; radiofluorination of the chloro species in partially aqueous media gives high radiochemical yields and, particularly for M = Ga, good radiochemical stability in PBS/EtOH at pH 7.4.

## Introduction

Fluorine-18 is the most widely used radioisotope in the clinic for positron emission tomography (PET) imaging due to its favorable characteristics, including a relatively short, but manageable, half-life (*ca*. 110 min), wide availability (cyclotron production), the dominance of β^+^ emission in its radio-decay pathway, favorable positron energy, and the lower toxicity of ^18^F (and the ^18^O decay product) relative to many metal radionuclides.

Advances in diagnostic imaging mean radiotracers with peptides incorporated that can target receptors which are overexpressed in unhealthy cells are often employed.^1^ Traditional C–^18^F labeling requires a high temperature and is therefore incompatible with peptides. Therefore, typically, radiofluorine is attached to a carbon center first, followed by conjugation to a peptide, resulting in multistep and often time-consuming synthesis. On the other hand, radiometal-based tracers incorporating bioconjugated targeting peptides often linked via the chelator unit are well-known. These bind to the target of interest, allowing highly selective imaging. Taking advantage of this, there has been significant interest over the past decade or so in exploiting highly fluorophilic main group compounds and metal-based complexes to achieve fast, late-stage radiolabeling under (partially) aqueous conditions, therefore simplifying the radiofluorination procedure.

Moreover, the recent advent of total body PET, for which clinicians may require multiple different, highly selective PET tracers to be administered in a short time frame, maximizes clinical information while minimizing radiation exposure for the patient. The net result is an increased need for new classes of PET tracers, using new types of chemistries, that can be prepared rapidly via simple procedures.

With this in mind, radiotracers based upon inorganic-fluoride compounds could offer several important advantages, opening up new chemistries, a single-step approach using a preformed metal chelate scaffold, direct radiofluorination, and simple purification under mild conditions.^2,3^ Certain main group and transition metal species show high fluoride affinities, which can result in favorable thermodynamics and fast reaction kinetics for binding to radiofluorine; these inorganic scaffolds can then be conjugated via the macrocyclic coligand to a range of peptides. In particular, work from a number of groups has focused on boron^4^ and silicon-based^5^ molecules bearing fluorine-18 as PET tracers, while trivalent main group metal ions (aluminum(III),^6−8^ gallium(III),^9−11^ indium(III)^12^) and transition metal ions (scandium(III)^13^ and iron(III)^14^) bearing mostly macrocyclic chelators based upon the tacn (1,4,7-triazacyclononane) core have attracted considerable interest as potential metal-chelate scaffolds for radiofluorine. Coordination to macrocyclic ligands increases the thermodynamic and kinetic stability of the complexes, therefore reducing the likelihood of byproduct formation during radiolabeling and reducing the likelihood of hydrolysis and liberation of ^18^F^–^*in vivo*.

The first metal-based complexes for ^18^F^–^ binding and PET imaging were reported by McBride and co-workers, leading to a range of Al–^18^F tracers, demonstrating that the judicious choice of chelator and metal provides an effective strategy for simple, late-stage radiofluorination (high radiochemical yields) and produces radiotracers with excellent stability under physiological conditions.^6^ A number of these systems are now undergoing clinical studies. More recent work has also employed a range of acyclic pentadentate ligands based upon N- and O-donor groups.^15^

Our own work has shown that ^18^F^–^ is readily incorporated into Al(III) and Ga(III) complexes with neutral tacn-based ligands, [MCl_3_(BnMe_2_-tacn)], via Cl/^18^F exchange reactions in partially aqueous MeCN,^9a^ while ^18^F/^19^F isotopic exchange using [MF_3_(BnMe_2_-tacn)] (M = Ga or Fe) occurs at sub-30-nM precursor concentrations, under mild, partially aqueous conditions.^9c^ These compounds also showed high ^18^F uptake and very good radiochemical purity (RCP) over several hours in PBS (phosphate buffered saline) and HSA (human serum albumin).

Following work based from the “Al–F” chemistry from McBride and co-workers and studies on the pentadentate Bn-NODA (1-benzyl-4,7-dicarboxylate-1,4,7-triazacyclononane) ligand with AlCl_3_ and ^18^F[F^–^] from Shetty et al.,^7^ we also reported a related Ga(III) species, [Ga^18^F(Bn-NODA)]. However, while this radio-complex was obtained in high radiochemical yield (RCY) and showed excellent stability up to pH = 6.5, liberation of fluoride occurred in PBS and HSA solutions (pH ∼ 7.5).^9b^ We suggested that this instability of the Ga(III) species at higher pH may reflect the strain caused by the acute chelate bite angles associated with the carboxylate pendant arms. This prompted consideration of replacing the carboxylate arms with phosphinate (−CH_2_P(O)(R)O^–^) groups akin to ligands developed by Parker and co-workers for radiometal binding, including with Ga(III), and which present less acute chelate bite angles involving the phosphinate arms.^16^

**Scheme 1:**
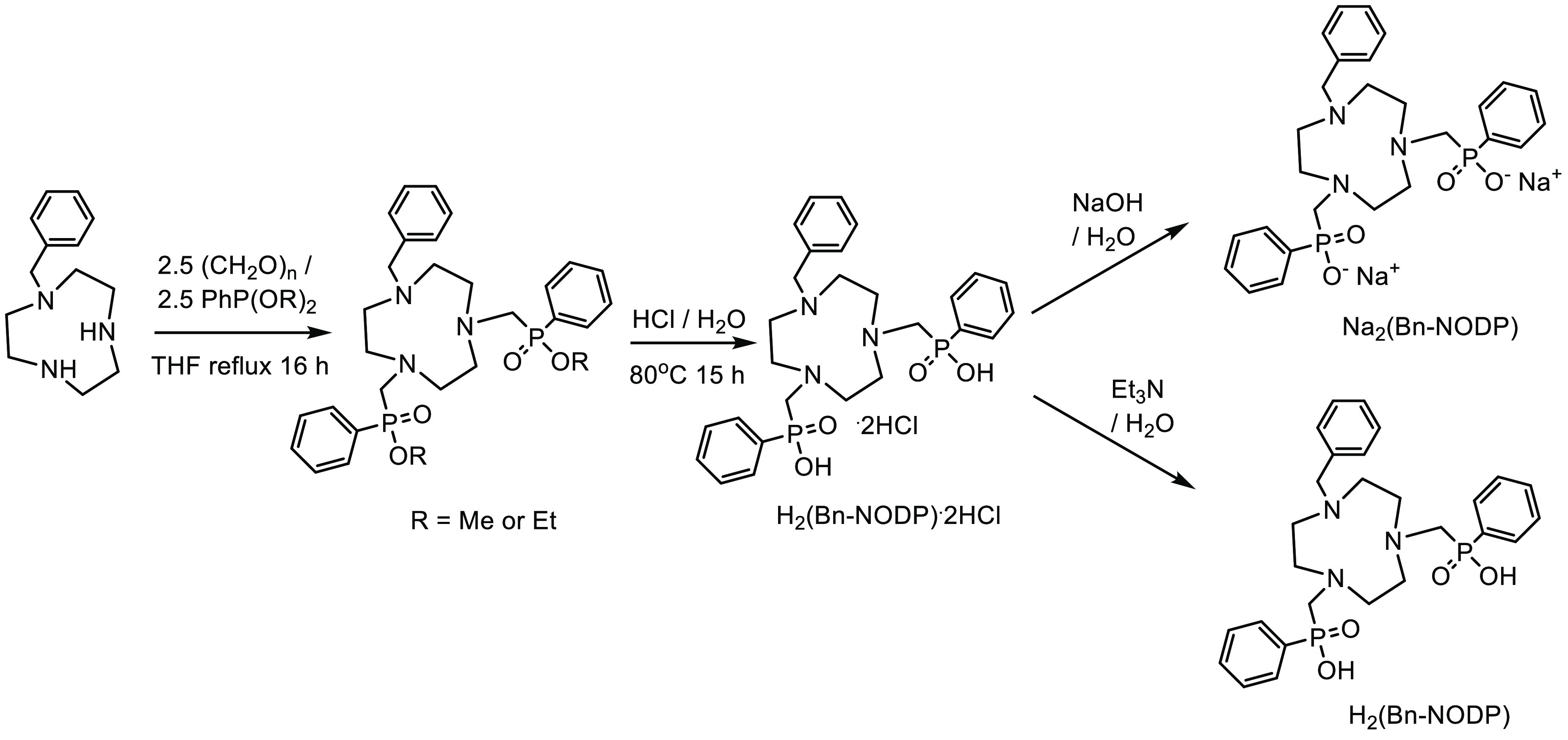
Synthesis Route for H_2_(Bn-NODP), along with Its HCl and Na^+^ Salts

We describe here the preparation of a novel *bis*-phosphinic acid functionalized macrocyclic ligand, H_2_(Bn-NODP); (Scheme 1), based upon the 1,4,7-triazacyclononane core, and its reactions to afford [MCl(Bn-NODP)] (M = Ga, Fe) and [MF(Bn-NODP)]·4H_2_O, the crystal structure of which is reported for M = Ga. Density functional theory (DFT) calculations (B3LYP-D3 and BP86-D3 functionals) have been undertaken to explore the electronic structures and relative stabilities of the possible geometric and stereoisomers of [MX(Bn-NODP)], and the possibility that hydrogen bonding may also play a role in determining the relative isomer energy order was also investigated by DFT and AIM (Atoms-in-Molecules) calculations. Finally, radiofluorination of [MCl(Bn-NODP)] in partially aqueous solvent is discussed.

## Results and Discussion

The method we employed for the preparation of the macrocyclic *bis*-phosphinic acid, H_2_(Bn-NODP) (and its salts, H_2_(Bn-NODP)·2HCl·4H_2_O and Na_2_(Bn-NODP)·5H_2_O, respectively), incorporating the tacn (1,4,7-triazacyclononane) macrocyclic core, was based upon a modification of the published procedures for the corresponding *tris*-phosphinic acid, H_3_(NOTP)·2HCl·2H_2_O.^16^ Thus, Bn-tacn (1-benzyl-1,4,7-triazacyclononane) was refluxed with ca. 2.5 mol. equiv of paraformaldehyde and diethoxyphenylphosphite (or dimethoxyphenylphosphite), followed by acid hydrolysis of the *bis*-phosphinate ester intermediate. The *bis*-phosphinic acid could be isolated as a hydrate of its HCl salt, H_2_(Bn-NODP)·2HCl·4H_2_O (Scheme 1), with δ(^31^P{^1^H}) = 34.55 (*d*_4_-MeOH) and ESI^+^ MS (MeOH) found to be *m*/*z* 528.4 ([Bn-NODP+H]^+^). However, it proved difficult to remove minor impurities from this salt, and the number of HCl and H_2_O molecules associated was difficult to ascertain reliably, although the product itself can be used directly for further reactions with metal salts. We found it to be preferable to isolate the ligand as its sodium salt, Na_2_(Bn-NODP)·5H_2_O, by adjusting to pH 12 with aqueous NaOH (Scheme 1), followed by purification by flash chromatography, allowing the ligand to be isolated as a white powdered solid in good yield. High resolution ESI^+^ MS (MeOH) shows the expected molecular ion in each case, and the presence of the associated water was determined via both elemental analysis and from X-ray crystallographic analysis (see below).

The ^1^H, ^13^C{^1^H}, and ^13^C DEPT-135 NMR spectra (*d*_4_-MeOH) of the H_2_(Bn-NODP) (and its HCl and Na^+^ salts) are consistent with incorporation of two chemically equivalent phosphinic acid functions into Bn-tacn, leading to three distinct tacn-CH_2_ groups in the ^13^C{^1^H} NMR spectra (the two CH_2_ groups adjacent to the N atoms bearing the phosphinate pendant arms), with the expected large doublet ^1^*J*_PC_ couplings evident on the *C*H_2_P and *ipso*-*C*P resonances, and smaller, longer-range couplings observed on the *o*-, *m*-, and *p*-*C*H groups of the phenyl ring directly bonded to P. The ^31^P{^1^H} NMR spectrum of the H_2_(Bn-NODP) shows a singlet at 27.21 ppm, between those of the HCl salt (34.55 ppm) and the Na^+^ salt (24.42 ppm). Similar trends were observed in the data reported for the *tris*-phosphinic acid derivative of tacn, H_3_(NOTP) (NOTP = 1,4,7-triphenylphosphinate-1,4,7-triazacyclononane).^16c^

**Figure 1:**
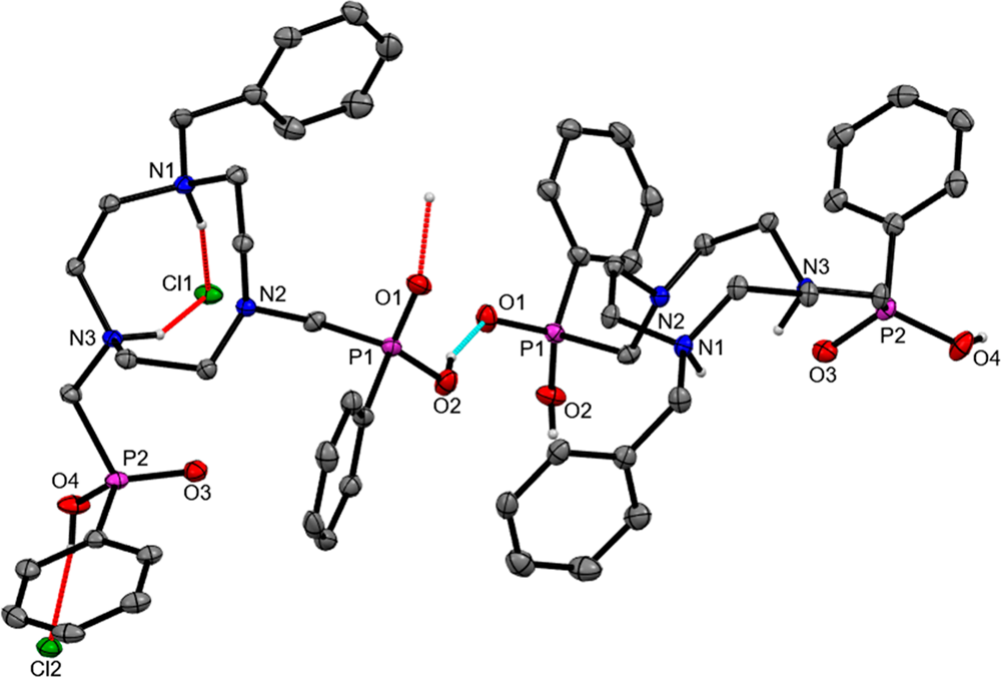
View of the structure of [H_2_(Bn-NODP)]·2HCl·H_2_O showing the atom labeling scheme and illustrating the H-bonding interactions (dashed lines). The ellipsoids are drawn at the 50% probability level, and H atoms, except those on heteroatoms, are omitted for clarity. H-bonding distances (Å): Cl1···N3 = 3.050, Cl1···N1 = 3.155, Cl1···O5 (lattice water) = 3.255, Cl2···O4 = 2.866, (P–OH···O=P) O1···O2 = 2.471.

Crystals of the HCl salt, [H_2_(Bn-NODP)]·2HCl·H_2_O, were obtained from a H_2_O/MeCN/MeOH solution of the salt over several weeks, and the X-ray structure confirms (Figure 1) that the H_2_(Bn-NODP) core is doubly protonated at the tacn ring, with one of the Cl^–^ anions (Cl1) H-bonded and bridging the two protonated N atoms. The second Cl^–^ anion (Cl2) is involved in H-bonding to a terminal P–OH group, and there is further H-bonding to a lattice water molecule and between a P–OH group in one macrocycle and a P=O group in a neighboring molecule (PO2···O1=P = 2.471 Å), leading to a weakly associated 1D chain in the solid state.

**Figure 2:**
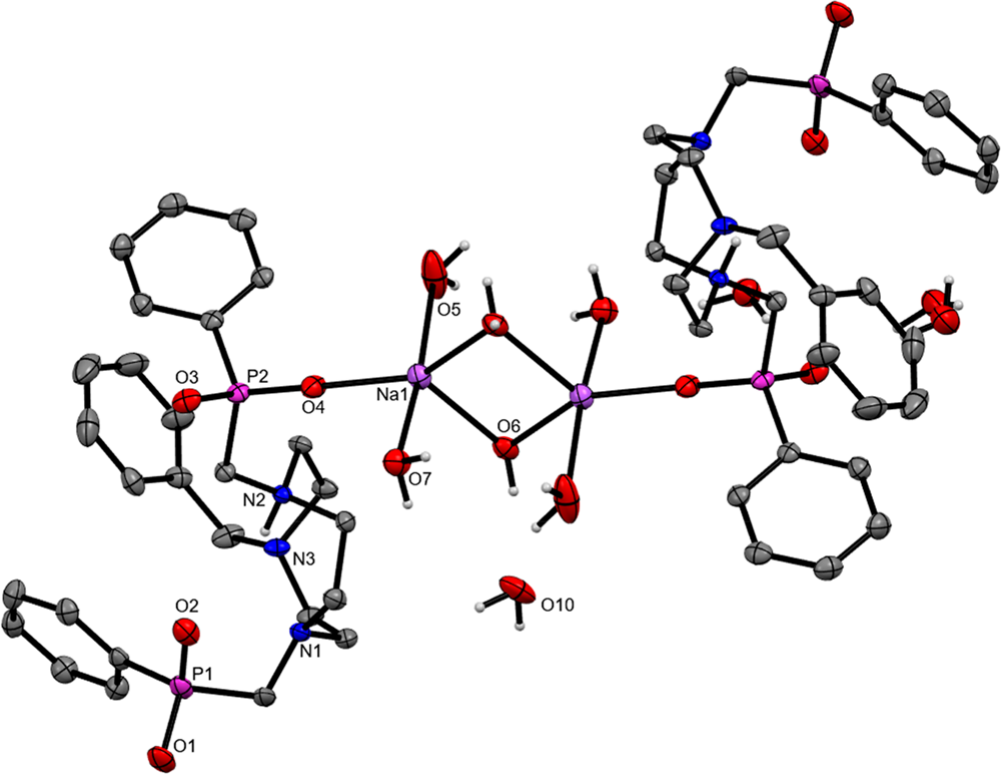
View of the structure of [{H(Bn-NODP)Na(H_2_O)_2_}_2_(μ-OH_2_)]·6H_2_O showing the atom labeling scheme. The ellipsoids are drawn at the 50% probability level. H atoms (except the H atoms associated with heteroatoms) are omitted for clarity. Selected bond lengths (Å) and angles (deg): Na1–O4 = 2.1779(15), Na1–O5 = 2.380(2), Na1–O6 = 2.4161(16), Na1–O7 = 2.3707(16), P1–O1 = 1.5076(14), P1–O2 = 1.5048(15), P2–O3 = 1.5037(13), P2–O4 = 1.4855(14), O4–Na1–O6 = 115.50(6).

A few crystals of a minor product were also obtained after several weeks from slow diffusion of Et_2_O into the MeOH filtrate after precipitation and removal of Na_2_(Bn-NODP)·5H_2_O. Upon crystallographic analysis, this species was shown to be the dimeric sodium complex, [{H(Bn-NODP)Na(H_2_O)_2_}_2_(μ-OH_2_)_2_]·6H_2_O (Figure 2), in which each macrocyclic ligand is associated with one sodium ion. In the dimer, each Na^+^ ion is five-coordinate (trigonal bipyramidal) through two axial water ligands, with two bridging waters and one O atom (from a phosphinate function on the macrocycle) in the equatorial plane. The second (uncoordinated) phosphinic acid pendant arm on each macrocycle is also deprotonated (i.e., phosphinate), and charge balance is achieved via a single proton bridging two amine N atoms of each of the tacn rings. There is further extensive H-bonding to solvent water molecules as illustrated in the SI (Figure S10). Coordination complexes containing a similar “(H_2_O)_2_Na(μ-H_2_O)_2_Na(H_2_O)_2_” core are well documented in the literature.^17^

The structures of [{H(Bn-NODP)Na(H_2_O)_2_}_2_(μ-OH_2_)_2_]·6H_2_O and the HCl salt described above exemplify the effect of pH on the speciation of H_2_(Bn-NODP) and the extent of H-bonding possible and suggest that rich coordination chemistry can be anticipated for this ligand.

### Preparation of [MCl(Bn-NODP)] (M = Ga, Fe)

**Scheme 2:**
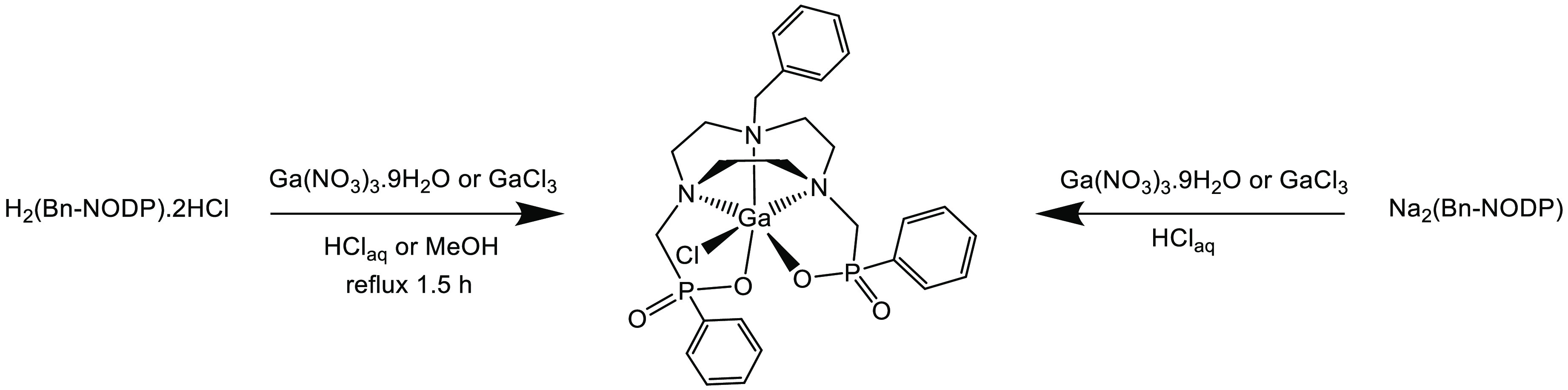
Methods for the Preparation of [GaCl(Bn-NODP)]

Several reactions using H_2_(Bn-NODP)·2HCl or Na_2_(Bn-NODP) with either Ga(NO_3_)_3_·9H_2_O or GaCl_3_ in water were performed as illustrated in Scheme 2, producing the target complex, [GaCl(Bn-NODP)]. In each case, the macrocyclic ligand acts as a dianionic chelator showing a high affinity for pentadentate coordination to the metal, with Cl^–^ completing the distorted six-coordinate environment at Ga(III), producing the neutral [GaCl(Bn-NODP)]·4H_2_O complex as a white powdered solid. ESI^+^ MS (MeOH) shows peaks with the correct isotopic distributions corresponding to [GaCl(Bn-NODP) + H]^+^, and one Ga–Cl stretching vibration is also evident in the IR spectrum (υ_Ga–Cl_ = 377 cm^–1^), as expected.

**Figure 3:**
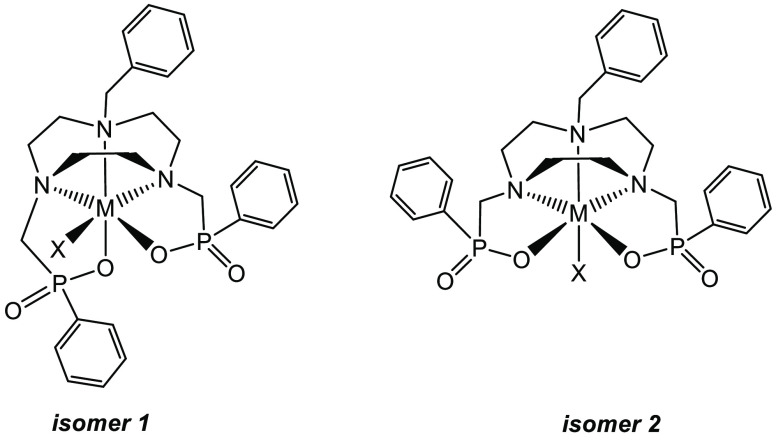
Two geometric isomers of [MX(Bn-NODP)] (note that the chirality at the P atoms can also give rise to *RR*, *SS*, *RS*, or *SR* forms of the ligand in each geometric isomer).

The six-coordinate [MX(Bn-NODP)] complexes can, in principle, exist as two distinct geometric isomers (Figure 3), one in which the phosphinate groups are inequivalent, with the coordinated O donor from one phosphinate lying *trans* to the N atom carrying the other phosphinate group, and the coordinated O donor of the second phosphinate lying *trans* to the N-Bn group (isomer 1), and the other in which the phosphinates are equivalent, with the coordinated O donors of each phosphinate lying *trans* to an N donor bearing a phosphinate pendant arm (isomer 2). Furthermore (assuming retention of the RRS/SSR chirality for the tacn ring nitrogens observed crystallographically), the chirality at the P atoms of the phosphinate groups can also give rise to four stereoisomers for isomer 1 and three for isomer 2. [The crystal structure of [GaF(Bn-NODP)]·4H_2_O shows the chirality at the tacn N atoms to be *RRS*/*SSR* (equivalent energies). This *RRS* stereochemistry for the tacn N atoms was retained throughout for the calculations concerning the effect of chirality at the phosphinate groups.]

**Figure 4:**
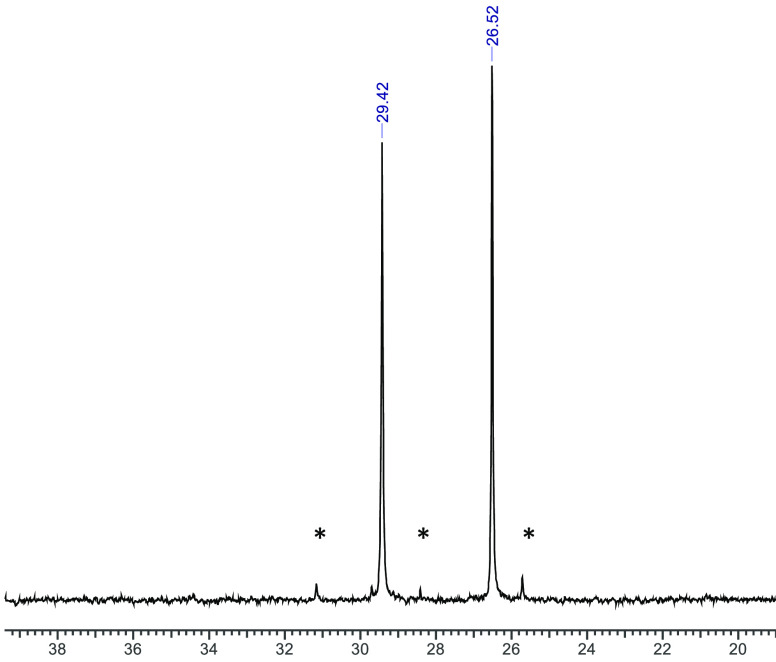
^31^P{^1^H} NMR spectrum of [GaCl(Bn-NODP)] in *d*_4_-MeOH (298 K). * = minor unidentified species.

The ^31^P{^1^H} NMR spectrum of the [GaCl(Bn-NODP)] complex in *d*_4_-MeOH (Figure 4) shows two singlets which integrate to a 1:1 ratio, with chemical shifts similar to those of the neutral H_2_(Bn-NODP), although we note that the δ^31^P{^1^H} values move around by a few parts per million depending on the synthesis route used, most likely reflecting secondary interactions between the phosphinate groups and solvent. This is consistent with isomer 1 being the only geometric form present in solution, and in accord with the ^31^P{^1^H} NMR spectrum expected from DFT calculations for this isomer (*vide infra*). In the ^1^H NMR spectrum, the H atoms on the C*H*_2_-Bn pendant group are also inequivalent, indicating that the Bn group is “locked”, as reported previously for the Al–F complex of a NODA derivative bearing a CH_2_–Ar pendant group.^4e^

**Scheme 3:**
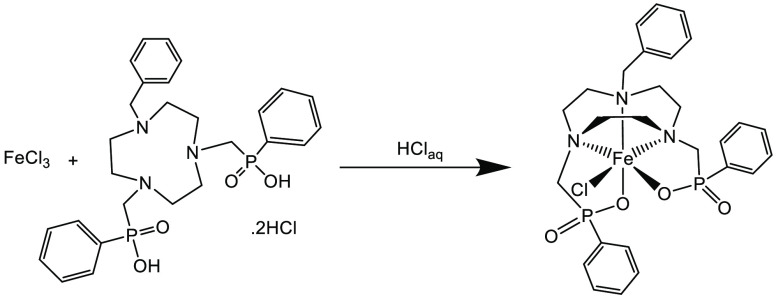
Method for the Synthesis of [FeCl(Bn-NODP)]

The high-spin (d^5^) Fe(III) complex, [FeCl(Bn-NODP)] (Scheme 3), is paramagnetic and therefore unsuitable for NMR analysis. However, it was identified by a combination of elemental analysis, IR spectroscopy (υ_Fe–Cl_ = 383 cm^–1^), and ESI^+^ MS (MeOH), showing *m*/*z* 617.2, with the expected isotope pattern.

With the required [MCl(Bn-NODP)] (M = Ga and Fe) complexes in hand, we proceeded to test their prospects as fluoride binding scaffolds, also with a view to characterizing the [M^19^F(Bn-NODP)] to use as references for the radio-hplc analyses, and prior to undertaking the radiofluorination experiments and potentially for radiolabeling via ^18^F/^19^F isotopic exchange.

The addition of 1 mol. equiv of aqueous KF to a solution of [GaCl(Bn-NODP)] in MeOH afforded a white solid, the IR spectrum of which showed a loss of the υ_Ga–Cl_ peak and the appearance of a new peak at 585 cm^–1^, consistent with υ_Ga–F_. Similarly, Cl/F exchange in the [FeCl(Bn-NODP)] complex with 1 equiv of KF led to a new peak at 575 cm^–1^ (υ_Fe–F_). The ESI^+^ MS data (MeOH) showed that the major peaks corresponded to [MF(Bn-NODP)+H]^+^ for both systems. ^31^P{^1^H} and ^19^F{^1^H} NMR data were also collected for the diamagnetic [GaF(Bn-NODP)] complex. The ^31^P{^1^H} spectrum showed a doublet and a singlet, indicating retention of the isomer 1 geometric form and where one phosphinate group couples to the coordinated fluoride to produce the doublet, with a small ^3^*J*_PF_ coupling of 3 Hz (SI Figure S6b). The ^19^F{^1^H} NMR spectrum is a broad singlet at −176.1 ppm. The broadening is most likely due to the direct bonding to the quadrupolar ^69/71^Ga isotopes (both *I* = 3/2), masking the small ^3^*J*_PF_ coupling. The NMR, IR, and mass spectra for Bn-NODP and the new complexes are presented in SI Figures S1–S9.

**Figure 5:**
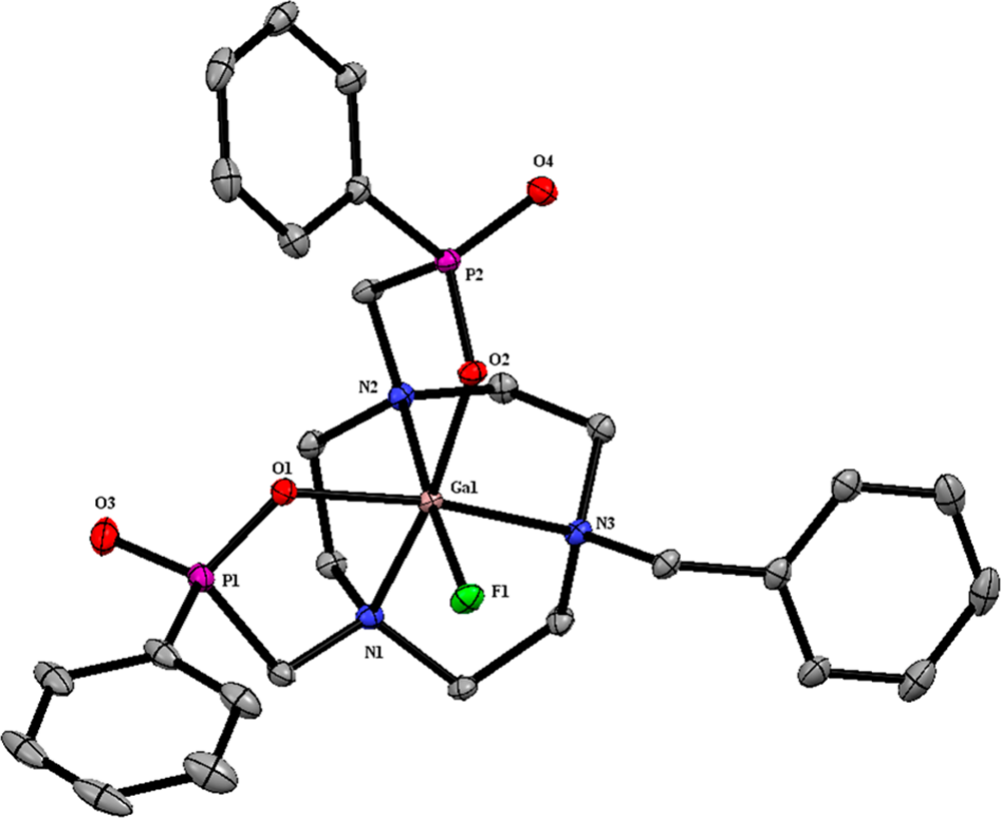
View of the structure of [GaF(Bn-NODP)]·4H_2_O showing the atom labeling scheme. The ellipsoids are drawn at the 50% probability level, and H atoms and lattice water molecules are omitted for clarity.

Finally, single crystals of [GaF(Bn-NODP)]·4H_2_O were grown by slow evaporation of an aqueous solution of the complex. The structure (Figure 5, Table 1) confirms pentadentate coordination of the dianionic Bn-NODP chelator to gallium(III) via an N_3_O_2_ donor set, with the fluoride ligand completing a distorted octahedral geometry. The [GaF(Bn-NODP)] molecules adopt the asymmetric isomer 1 form, with the phosphinates in the *RR*, i.e., (N)*RRS*-(P)*RR* configuration) and its (N)*SSR*-(P)*SS* enantiomer (see also footnote 1), and with *d*(Ga–F) = 1.832(1) Å, *d*(Ga–O) = 1.936(1) and 1.940(2) Å, and three slightly longer *d*(Ga–N) in the range 2.141(2)–2.153(2) Å. Notably, one of the (nonbonded) P···F distances in the molecule is ca. 1 Å shorter than the other, 3.529 (P1–F1) and 4.477 Å (P2–F1), respectively, which may account for the observation of the small P–F doublet coupling on just one of the ^31^P{^1^H} NMR resonances.

**Table 1:** Selected Bond Lengths and Angles for [GaF(Bn-NODP)]·4H_2_O

bond lengths/Å			
Ga1–F1	1.832(1)	P1–O1	1.536(2)
Ga1–O2	1.936(1)	P1–O3	1.493(2)
Ga1–O1	1.940(2)	P2–O2	1.536(1)
Ga1–N1	2.141(2)	P2–O4	1.496(2)
Ga1–N2	2.150(2)		
Ga1–N3	2.153(2)		

bond angles/deg			
O1–Ga1–N1	86.00(7)	F1–Ga–N1	95.85(6)
O1–Ga1–N2	93.69(7)	F1–Ga–N2	173.10(6)
O2–Ga1–N2	85.40(6)	F1–Ga–N3	90.38(6)
O2–Ga1–N3	98.92(6)	F1–Ga1–O1	92.27(6)
O1–P1–C10	102.8(1)	F1–Ga1–O2	97.84(6)
O2–P2–C19	102.73(9)		

The Ga–O and Ga–N bond distances in [GaF(Bn-NODP)] compare well with those found in the C_3_-symmetric [Ga(NOTP)]·5H_2_O (space group: *P*3̅), *d*(Ga–O) = 1.912(4) and *d*(Ga–N) = 2.135(6) Å.^16b^ However, turning to the chelate angles involving the pendant phosphinate groups, in [GaF(Bn-NODP)]·4H_2_O we observe ∠(O1–Ga–N1) = 86.00(7) and ∠(O2–Ga–N2) = 85.40(6)°; while these compare very closely with ∠(O–Ga–N) = 86° in [Ga(NOTP)]·5H_2_O,^16b^ they are *ca*. 3–4° larger than the corresponding O–Ga–N chelate angles in the previously reported structure of [GaF(Bn-NODA)]·2H_2_O,^9b^ suggesting that the Bn-NODP ligand is a better fit for the Ga(III) ion.

Further analysis of the extended structure also reveals significant F···H–O and O···H–O hydrogen-bonding interactions involving both the coordinated, highly electronegative fluoride ligand with the lattice water molecules, and between lattice waters. Part of the resulting 3D assembly is shown in SI Figure S7.

Collectively, these preparative scale experiments support our hypothesis that Bn-NODP may be a superior pentadentate ligand scaffold for effective binding to the Ga–^18^F or Fe–^18^F moieties in subsequent radiochemistry experiments and for maintaining the integrity of the complex under physiological conditions (pH 7.4), compared to our previous findings for [Ga^18^F(Bn-NODA)].^9b^

### Density Functional Theory Calculations

The observation that only one geometric isomer, isomer 1, is formed for the gallium(III) halide complexes of Bn-NODP (both in solution and in the solid state) is likely to be, at least in part, a consequence of the coordinated Bn-NODP ligand forming only five-membered chelate rings, all of which lead to angles subtended at gallium(III) that are significantly below 90°. In isomer 1, only two adjacent chelate rings lie in the same plane, whereas the more symmetrical isomer 2 places three adjacent five-membered rings coplanar, leading to a more strained geometry.

To gain insight into the electronic structures and relative stabilities of the two geometric forms, isomer 1 and isomer 2 of [GaX(Bn-NODP)] (X = F, Cl) and their stereoisomers, a series of density functional theory (DFT) calculations were performed on isolated structures. For the seven isomeric forms of [GaF(Bn-NODP)], the geometries were optimized and the relative energies (Δ*E*) and standard free energies (Δ*G*_298K_^ϕ^) were computed. The results obtained are shown in Table 2. The lowest energy isomer at both the B3LYP-D3 and BP86-D3 levels is isomer 1/*RR*, with isomer 2/*RS* the second lowest energy isomer. Table 2 also shows the calculated sum of hydrogen bonding (HB) energies for each isomer. It was found that, although H-bonding is important in these structures, for example at the BP86-D3 level, isomer 1/*RR* has 11 HBs (4 F···H, 6 O···H, 1 C···H HBs), there is no obvious trend in HB values with the relative energy of each isomer. Therefore, although H-bonding contributes to the total energy of each isomer, it is not the major factor in determining the lowest energy structure or relative energy order (see Tables 2 and S2). To investigate this further, the nuclear–nuclear repulsion energy (*E*_nn_) was obtained for each minimum energy structure. The total energy (E_TOT_) can be written as *E*_TOT_ = *E*_elect_ + *E*_nn_, where *E*_elect_ is the electronic energy, made up of electron kinetic energy (*E*_T_), electron–nuclear (*E*_v_), coulomb (*E*_coul_), exchange (*E*_x_), and electron correlation (*E*_corr_) terms. Values for *E*_nn_ for all seven minimum energy structures were computed (see Table S3; Table S2 lists values of *E*_TOT_). At the B3LYP-D3 level, the lowest *E*_nn_ structure was found to be isomer 1/*RR*, with isomer 2/*RS* slightly higher, whereas at the BP86-D3 level, isomer 2/*RS* had the lowest *E*_nn_ with isomer 1/*RR* slightly higher. However, as shown in Table 2 and Table S2, when *E*_elect_ is included, values of *E*_TOT_ place isomer 1/*RR* as the lowest, with both functionals. This means that *E*_nn_ and *E*_elect_ both make significant contributions in determining the relative energy of the seven isomers considered. The relative energy order of the isomers is the same for both functionals.

**Table 2:** Relative Energies (Δ*E* and Δ*G*_298K_^ϕ^) of [GaF(Bn-NODP)] Isomers at the B3LYP-D3 and BP86-D3 Levels

	B3LYP-D3			BP86-D3		
isomer	relative energy, Δ*E*/kcal mol^–1^	relative free energy, Δ*G*_298K_^ϕ^/kcal mol^–1^	sum of HB energies, ∑ HB_i_/kcal mol^–1^ (no. of HBs)	relative energy, Δ*E* a.u./kcal mol^–1^	relative free energy, Δ*G*_298K_^ϕ^/kcal mol^–1^	sum of HB energies, ∑HB_i_/kcal mol^–1^ (no. of HBs)
isomer 1*/SS*	15.89	15.42	20.25 (11)	13.42	13.17	22.20 (12)
isomer 2*/SS*	12.46	12.45	29.30 (11)	10.83	9.86	31.18 (12)
isomer 2/*RR*	11.34	11.34	26.52 (11)	10.11	10.88	20.63 (11)
isomer 1*/SR*	8.05	8.02	26.10 (12)	6.78	6.81	27.24 (12)
isomer 1/*RS*	7.79	7.39	22.79 (10)	6.54	6.20	26.42 (12)
isomer 2/*RS*	5.31	5.25	29.90 (10)	5.02	5.02	30.73 (10)
isomer 1/*RR*	0.00	0.00	27.34 (11)	0.00	0.00	30.08 (12)

As four lattice water molecules were found to be H-bonded with each [GaF(Bn-NODP)] unit of isomer 1/*RR* in the crystal structure, to confirm that their effect on the optimizations is small, calculations were also carried out on this isomer both with and without these peripheral water molecules, at the B3LYP-D3 and BP86-D3 levels. They were (a) a fixed geometry calculation using the geometrical parameters of isomer 1/*RR* from the crystal structure, with the four water molecules present in their crystal structure positions; (b) a fixed geometry calculation as in (a), with no water molecules present; (c) a full geometry optimization calculation using the geometrical parameters of isomer 1/*RR* from the crystal structure, with the four water molecules initially in their crystal structure positions; and (d) a full geometry optimization calculation as in (c), with no water molecules present.

The results of these calculations shown in Figure S17 and Table S10 confirm that the water molecules move only slightly from their crystal structure positions on optimization. Also, the computed geometrical parameters of [GaF(Bn-NODP)] isomer 1/*RR* change only very slightly with and without added water. Comparison of the optimized geometrical parameters (bond lengths and angles), with and without the water molecules, with the experimental parameters from the crystal structure shows good agreement at both the B3LYP-D3 and BP86-D3 levels (Table S10).

Table 3 shows the computed (BP86-D3) Mulliken charges on the Ga atom and the six atoms bonded to it (F, N, N, N_(trans-Bn)_, O, O) for [GaF(Bn-NODP)] isomer 1/*RR* for the cases (a)–(d). For all cases, gallium has a positive charge, whereas the donor atoms carry negative charges, with the largest negative charge on the coordinated O atoms of the phosphinate groups.

**Table 3:** Computed BP86-D3 Mulliken Charges on the Ga Atom and the Six Atoms Bonded to Ga (F, N, N, N_(trans-Bn)_, O, O) for the Cases (a)–(d) Listed in the Text for [GaF(Bn-NODP)] Isomer 1/*RR*

atom	[GaF(Bn-NODP)] case (a) (X-ray structure, with four waters)	[GaF(Bn-NODP)] case (b) (X-ray structure, no water)	[GaF(Bn-NODP)] case (c) (optimized, with four waters added)	[GaF(Bn-NODP)] case (d) (optimized, no water)
Ga	1.210	1.173	1.333	1.296
F	–0.506	–0.479	–0.502	–0.477
N	–0.555	–0.554	–0.490	–0.480
N	–0.542	–0.536	–0.496	–0.489
N_(*trans* Bn)_	–0.551	–0.550	–0.503	–0.490
O	–0.626	–0.625	–0.686	–0.703
O	–0.625	–0.626	–0.723	–0.722

On going from (b) → (a) and (d) → (c) (i.e., with the four peripheral water molecules present in cases (a) and (c), compared with (b) and (d), the positive charge on gallium shows a very small increase (see Table 3). For the seven isomers of [GaF(Bn-NODP)], the computed Mulliken charges on the Ga atom and the six atoms bonded to Ga in the optimized structures (B3LYP-D3 and BP86-D3 levels) are listed in Table S5, as well as the computed Mulliken charges on all atoms for structures isomer 1/*RR* and isomer 2/*RS*.

If the Ga–F unit can be viewed as (Ga^3+^F^–1^)^2+^ before coordination with the macrocyclic ligand (charge −2; L^2–^) i.e., gallium has a nominal initial charge of +3, then on complexation, electron transfer occurs from the five ligand atoms (N, N, N_(*trans*-Bn)_, O, O) to Ga, which reduces the positive charge on Ga to about +1.3 (as in cases c and d). Table S9 compares BP86-D3 atomic charge densities for [GaF(Bn-NODP)] isomer 1/*RR* with computed atomic charge densities for (Ga–F)^2+^ and L^2–^ units (with the Ga–F and ligand groups having the same geometrical parameters as in the complex). This table shows that, on forming the complex, the total charge on Ga reduces from +1.97 to +1.30 and the charge of F changes from +0.03 to −0.47. The negative charges on all other ligand donor atoms bonded to Ga (N, N, N_(*trans*-Bn)_, O, O) also increase, with the total negative charge on these five atoms increasing on coordination as electron density is drawn from the rest of the macrocyclic unit. If the [GaF(Bn-NODP)] isomer 1/*RR* complex were to be formed from neutral GaF combined with a neutral ligand, similar trends in changes in atomic charges on coordination are observed, but the charge on Ga in the GaF unit (+0.44) is much lower than that computed in the complex (+1.30). [GaF(Bn-NODP)] is, therefore, closer to (GaF)^2+^(Bn-NODP)^2–^ than (GaF)^0^(Bn-NODP)^0^.

Similar calculations performed on the seven isomers of the chloro analog, [GaCl(Bn-NODP)], also showed isomer 1/*RR* to be the lowest in energy. The computed bond lengths and angles for this isomer are shown in Table S4, and the Mulliken charges on the atoms bonded to Ga, listed for each isomer in Table S6, are very similar to those obtained for [GaF(Bn-NODP)], with the charge on Ga being slightly greater in [GaF(Bn-NODP)] than in [GaCl(Bn-NODP)].

Computed AIM hydrogen bond (HB) strengths of the seven [GaF(Bn-NODP)] isomers are shown in Table S7, and AIM HB results obtained for [GaCl(Bn-NODP)] isomer 1/*RR* are in Table S8 (at the B3LYP-D3 and BP86-D3 levels). The forms of some of the molecular orbitals (HOMO, HOMO–1, LUMO, LUMO+1) of isomer 1/*RR* of [GaF(Bn-NODP)] are shown in Figures S11–14, showing them to be concentrated on the aromatic rings of the molecules rather than on the metal. Figures S15 and S16 show the equivalent diagrams for isomer 2/*RS* at the BP86-D3 and B3LYP-D3 levels.

### Radiofluorination of [MCl(Bn-NODP)] (M = Ga, Fe)

**Scheme 4:**
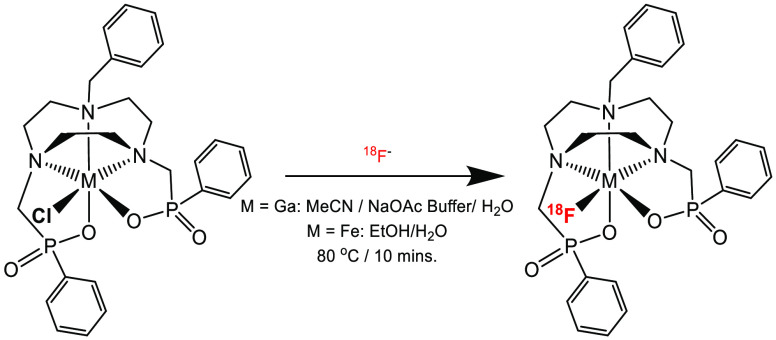
Conditions for Radiofluorination of [MCl(Bn-NODP)]

Radiofluorination experiments were performed via Cl^–^/^18^F^–^ halide exchange reactions (using ^18^F^–^ in target water directly) on [MCl(Bn-NODP) (M = Ga and Fe) using starting activities between 80 and 200 MBq, in partially aqueous MeCN (M = Ga) or EtOH (M = Fe), using either 1 mg or 0.1 mg of [MCl(Bn-NODP)] per milliliter of solvent and heating at 80 °C for 10 min (Scheme 4). The ^18^F^–^ incorporation was measured by integration of the radio-hplc traces. For [GaCl(Bn-NODP)], the radiochemical yield was increased by the addition of 0.3 mL of MeCN to the reaction medium to aid solubility.

**Figure 6:**
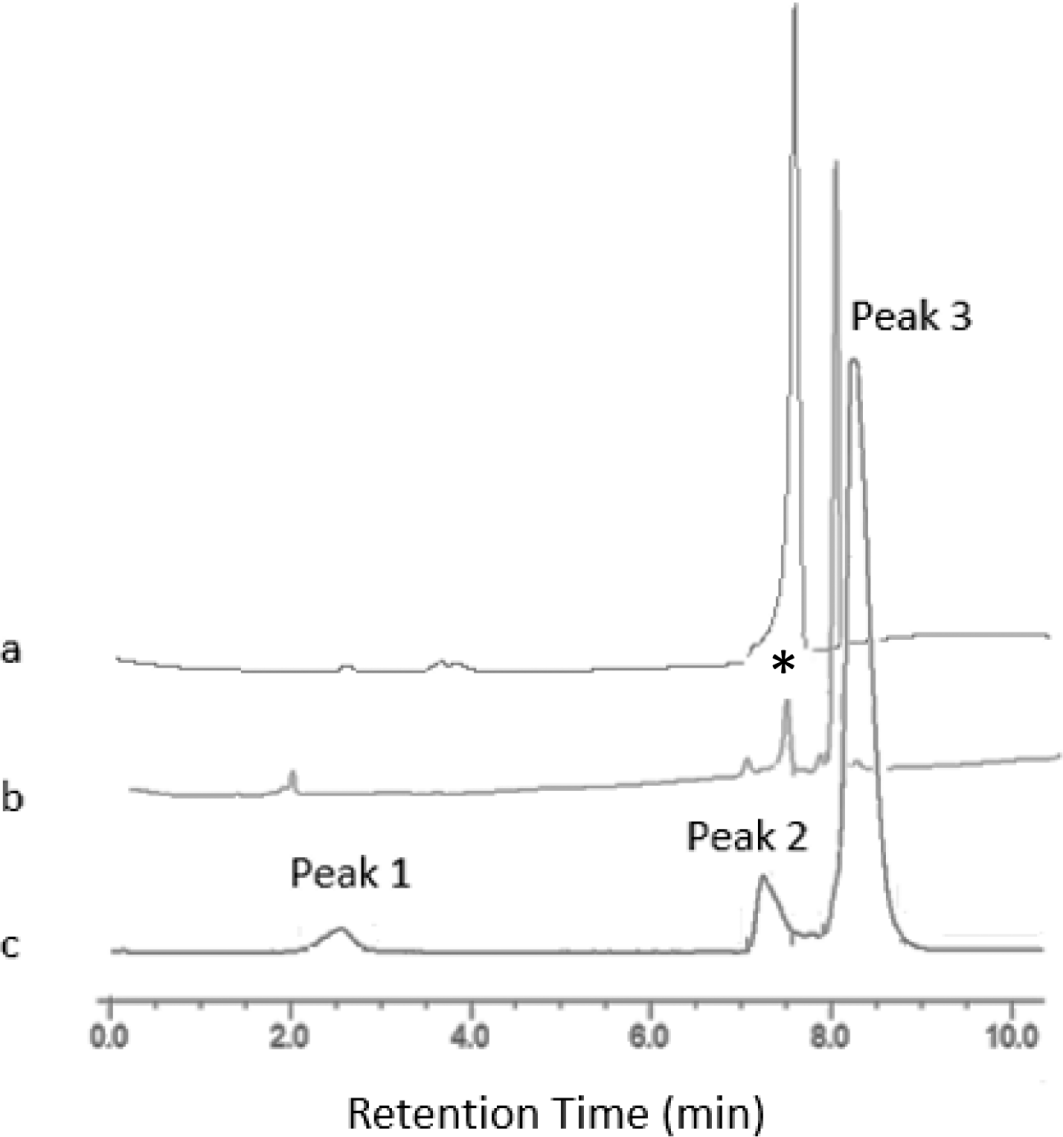
(a) Analytical UV-HPLC trace of the reference standard compound [GaCl(Bn-NODP)] (*R*_t_ = 7.66 min); (b) analytical UV-HPLC trace of the reference standard compound [Ga^19^F(Bn-NODP)] (*R*_t_ = 8.19 min); (c) analytical radio-HPLC trace of the crude product from radiofluorination of [GaCl(Bn-NODP)] (1 mg mL^–1^). Peak 1: *R*_t_ = 2.57 min 3.4% ([^18^F]F^–^]. Peak 2: *R*_t_ = 7.25 min 8.0% (unidentified). Peak 3: *R*_t_ = 8.27 min 88.6% ([Ga^18^F(Bn-NODP)]). (* = UV peak from residual [GaCl(Bn-NODP)].)

The radiofluorination of [GaCl(Bn-NODP)] resulted in a ∼71% radiochemical yield (RCY) when starting with 1 mg of precursor per milliliter (1.424 μM concentration). When using a lower concentration of 142 nM, the RCY dropped to 6%. The identity of the main radio-product was confirmed by comparison with the UV trace of the inactive reference standard, [Ga^19^F(Bn-NODP)], and a minor, as yet unidentified, radio impurity (which could be a small amount of a different isomer) was also observed with Rt = 7.25 min (Figure 6, Table 4). Purification to remove unreacted ^18^F^–^ was achieved using a solid phase extraction (SPE) cartridge method (see Experimental Section) before formulating the radioproduct in either 90:10 H_2_O/EtOH or 90:10 PBS/EtOH to investigate the radiochemical stability over time. The radiochemical stability of purified [Ga^18^F(Bn-NODP)] was monitored over time for a range of samples. After SPE purification (RCP at *t* = 0 was 100%), the RCP was 95% after 3.5 h, thus showing no significant loss of [^18^F]F^–^ from the main [Ga^18^F(Bn-NODP)] radioproduct when formulated in 90:10 H_2_O/EtOH. The ratio of the minor radio impurity also remained proportional to the main radioproduct.

**Table 4:** Conditions Used for Cl/^18^F Radiofluorination Experiments; Total Solvent Volume 1 mL in Each Case

precursor	mass (mg)	precursor conc. (nm)	solvent	*T*/°C (time/min)	% RCY
[GaCl(Bn-NODP)]·4H_2_O	1	1424	75:25 (EtOH/H_2_O)	80 (10)	58 ± 5^a^
[GaCl(Bn-NODP)]·4H_2_O	1	1424	45:30:25 (NaOAc/MeCN/H_2_O)	80 (10)	71 ± 15^b^
[GaCl(Bn-NODP)]·4H_2_O	0.1	142	45:30:25 (NaOAc/MeCN/H_2_O)	80 (10)	6 ± 2^b^
[FeCl(Bn-NODP)]·3H_2_O	1	1511	75 25 (EtOH/H_2_O)	80 (10)	83 ± 9^b^
[FeCl(Bn-NODP)]·3H_2_O	0.1	151	75:25 (EtOH/H_2_O)	80 (10)	77 ± 6^a^

a *N* = 2.

b *N* = 3.

**Figure 7:**
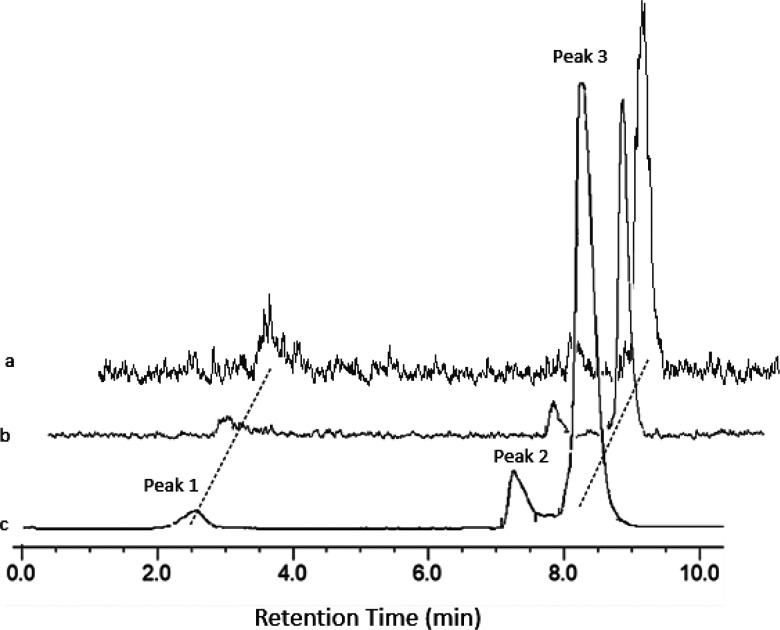
(a) Analytical radio-HPLC of the crude product from radiofluorination of [GaCl(Bn-NODP)] (1 mg mL^–1^). Peak 1: *R*_t_ = 2.57 min 3.4% ([^18^F]F^–^]. Peak 2: *R*_t_ = 7.25 min 8.0% (unidentified). Peak 3: *R*_t_ = 8.27 min 88.6% ([Ga^18^F(Bn-NODP)]). (b) analytical radio-HPLC trace of the purified product eluted from an HLB cartridge (formulated in 10% EtOH/90% PBS). Peak 1: *R*_t_ = 2.717 min 3.7% ([^18^F]F^–^]. Peak 2: *R*_t_ = 7.39 min 7.3% (unidentified). Peak 3: *R*_t_ = 8.38 min 88.9% ([Ga^18^F(Bn-NODP)]). (c) Analytical radio-HPLC trace of the purified product eluted from an HLB cartridge (formulated in 10% EtOH/90% PBS after 240 min. Peak 1: *R*_t_ = 2.77 min 8.8% ([^18^F]F^–^]). Peak 2: *R*_t_ = 7.29 min 6.2% (unidentified). Peak 3: *R*_t_ = 8.37 min 85.0% ([Ga^18^F(Bn-NODP)]).

Formulating in 90:10 PBS/EtOH also gave a very high RCP (∼96%) after 3.5 h (Figure 7, Table 5). For comparison, the previously reported studies of [Ga^18^F(Bn-NODA)], obtained with RCY = 65–70% in NaOAc buffer after heating at 80 °C for 30 min, showed a very low RCP in serum/EtOH or PBS/EtOH, due to rapid liberation of ^18^F^–^.^9b^ It was suggested that this was as a result of cleavage of the Ga–O(carboxylate) bonds, opening up the coordination sphere and promoting hydrolysis. The replacement of the carboxylate pendant arms with phosphinate groups in [Ga^18^F(Bn-NODP)] described in this work indeed leads to significantly improved stability at physiologically relevant pH.

**Table 5:** Radiochemical Purity (RCP) As a Function of Time in Different Formulations (at 1 mg of Precursor per mL) for [M^18^F(Bn-NODP)] (M = Ga, Fe)

[Ga^18^F(Bn-NODP)]	% RCP	
time/h	10:90 EtOH/H_2_O	10:90 EtOH/PBS
0	100	100
1	96	95
2.5	94	94
3.5	94	95

[Fe^18^F(Bn-NODP)]	% RCP	
time (h)	10:90 EtOH/H_2_O	10:90 EtOH/PBS
0	92	88
1	90	2
2	74	0
3	48	0
4	35	0

Due to the much higher stability observed for the phosphinate-based [Ga^18^F(Bn-NODP)] in PBS/EtOH compared to the carboxylate-based [Ga^18^F(Bn-NODA)], additional radiolabeling experiments to test whether the NaOAc buffer was necessary to generate [Ga^18^F(Bn-NODP)] were also carried out in aqueous EtOH in the absence of NaOAc. These experiments gave an RCY of 58 ± 5%. The precursor was found to be less soluble in EtOH, which may contribute to the lower RCY observed.

Radiofluorination via ^18^F/^19^F Isotopic exchange from [Ga^19^F(Bn-NODP)] was also investigated. However, under analogous conditions, these experiments failed to show any uptake of ^18^F^–^.

**Figure 8:**
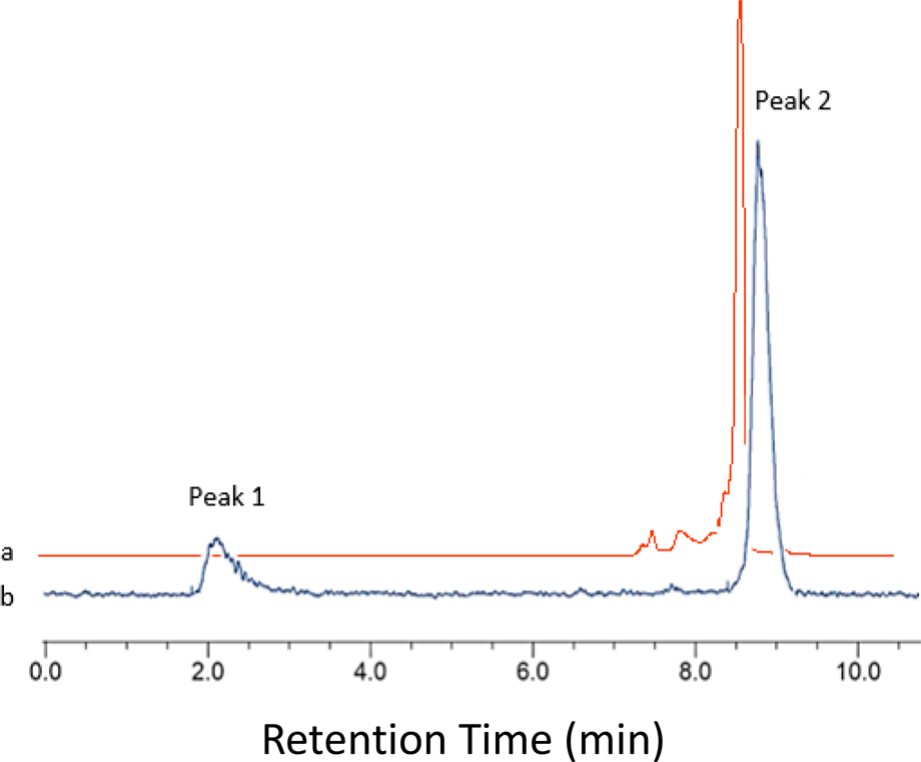
(a) Analytical UV-HPLC trace (red) for the reference standard compound [Fe^19^F(Bn-NODP)] (*R*_t_ = 8.69 min); (b) analytical radio-HPLC trace (blue) of the crude product from radiofluorination of [FeCl(Bn-NODP)] (1 mg mL^–1^). Peak 1: *R*_t_ = 2.14 min 17.1% ([^18^F]F^–^]. Peak 2: *R*_t_ = 8.80 min 82.9% ([Fe^18^F(Bn-NODP)]).

Radiofluorination experiments on the [FeCl(Bn-NODP)] complex using the same conditions as above, in 75:25 EtOH/H_2_O, led to higher RCY values of 83% and 77% (for 1 and 0.1 mg/mL, respectively) compared to the gallium complex. These radiofluorination experiments produced a single radioproduct in each case, which was identified as [Fe^18^F(Bn-NODP)] via comparison of the HPLC-UV trace of the [Fe^19^F(Bn-NODP)] reference standard (Figure 8). Subsequent SPE purification with an HLB cartridge and formulation in 90:10 PBS/EtOH showed the rapid complete liberation of [^18^F^–^] over time, although the complex showed better stability in 90:10 H_2_O/EtOH. This lack of stability in PBS is in contrast to the radiofluorination of [FeF_3_(BnMe_2_-tacn)], which did not show liberation of ^18^F^–^.^14^

The radiochemical purities (RCPs) of the various formulated solutions over time are shown in Table 5 and graphically in Figure 9. Additional HPLC radiotraces are shown in Figures S18–S26.

**Figure 9:**
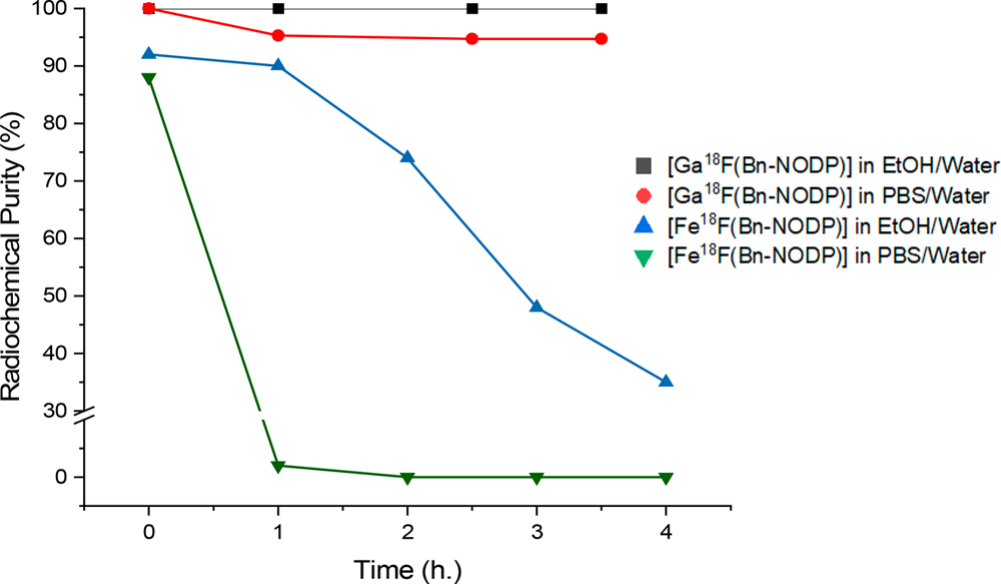
Graph showing the change in RCP as a function of time for [M^18^F{Bn-NODP)] in various formulations.

## Experimental Section

Ga(NO_3_)_3_·9H_2_O, GaCl_3_, FeCl_3_, paraformaldehyde, KF (Sigma-Aldrich), and Bn-tacn (Chematech) were used as received. PhP(OMe)_2_ (Alfa Aesar) and PhP(OEt)_2_ (Strem) were dried over 4 Å sieves before use. THF was dried by distillation from Na/benzophenone ketyl. The ligand and complex syntheses were carried out using standard Schlenk and vacuum line techniques. Chromatographic purification of 1-benzyl-4,7-*bis*(methylenephenylphosphinic acid)-1,4,7-triazacyclononane (Na_2_–Bn-NODP and H_2_–Bn-NODP) used a Biotage Selekt flash chromatography system (reverse phase SFar C18 column, 0–60% MeCN:H_2_O).

Infrared spectra were recorded using a PerkinElmer Spectrum 100 spectrometer in the range 4000–200 cm^–1^, with samples prepared as Nujol mulls between CsI plates. ^1^H, ^13^C{^1^H}, and ^13^C DEPT-135 NMR spectra were recorded using a Bruker AV 400 spectrometer and referenced to the residual protio-resonance of the solvent. ^19^F{^1^H} and ^31^P{^1^H} were recorded in *d*_4_-MeOH solutions using a Bruker AV 400 spectrometer and referenced to CFCl_3_ and external 85% H_3_PO_4_, respectively. Spectra were recorded at 295 K unless indicated otherwise. Samples were analyzed by ESI^+^ MS using a Waters Acquity TQD mass tandem quadrupole mass spectrometer. Samples were introduced to the mass spectrometer via an Acquity H-Class quaternary solvent manager (with TUV detector at 254 nm, sample and column manager). Gradient elution was from 20 to 100% MeCN/0.1% formic acid (FA)/H_2_O/0.1% FA over 5 min at a flow rate of 0.6 mL/min. Duplicate microanalyses were performed by Medac Ltd., with the majority of measurements within ±0.4% of the theoretical value. However, in a few cases, the values are slightly outside this range, probably reflecting both the recognized inherent variability of microanalytical measurements across different facilities^18^ and the variable amounts of water of crystallization in the samples.

### Synthesis of [H_2_(Bn-NODP)]·2HCl·4H_2_O

1-Benzyl-1,4,7-triazacyclononane (2.00 g, 9.1 mmol) was dissolved in THF (20 mL). PhP(OMe)_2_ (3.72 g, 21.9 mmol) and (CH_2_O)_*n*_ (0.66 g, 21.9 mmol) were added, and the solution was refluxed for 16 h. The solvent was removed *in vacuo*, and the phosphinate ester intermediate was obtained as a yellow oil. Then, 6 M HCl (20 mL) was added, and the solution was heated to 80 °C for 15 h under N_2_. Solvent was removed *in vacuo* and the residue washed with Et_2_O and MeCN several times to leave a white solid. Colorless crystals were grown from slow evaporation of MeOH and Et_2_O over a few days. Yield: 4.27 g, 59%. Required for C_27_H_35_N_3_O_4_P_2_·2HCl·4H_2_O (672.51): C, 48.22; H, 6.74; N, 6.24%. Found: C, 48.58; H, 5.89; N, 5.74%. ^1^H NMR (*d*_4_-MeOH/ppm): δ 7.80 (m, [4H], Ar-*H*), 7.71 (m, [2H]), 7.55 (m, [9H], Ar-*H*), 4.48 (s, [2H], NC*H*_2_Ar), 3.45 (br m, [2H], C*H*_2_, tacn), 3.35 (br m, [2H], C*H*_2_, tacn), 3.23 (d, ^1^*J*_PH_ = 4.8 Hz, [4H], PC*H*_2_), 3.14–2.62 (br m, [8H], C*H*_2_, tacn). ^13^C{^1^H} NMR (*d*_4_-MeOH/ppm): δ 134.16 (d, ^4^*J*_PC_ = 2.9 Hz, *p*-*C*H, Ph), 133.93 (s, *ipso*-C, Bn), 132.64 (d, ^3^*J*_PC_ = 10.3 Hz, *m*-*C*H, Ph), 132.64 (*m*-*C*H, Bn), 132.01 (d, ^1^*J*_PC_ = 119.6 Hz, *ipso*-*C*, Ph), 131.68 (*p*-*C*H, Bn), 130.67 (*o*-*C*H, Bn), 130.20 (d, ^2^*J*_PC_ = 12.5 Hz, *o*-*C*H, Ph), 61.28 (*C*H_2_Bn), 56.09 (d, ^1^*J*_PC_ = 108.6 Hz, *C*H_2_P), 53.06 (*C*H_2_NBn), 52.77, 51.07 (br, 2 × *C*H_2_, tacn). ^31^P{^1^H} (*d*_4_-MeOH/ppm): δ 34.55 (s). IR (Nujol/cm^–1^): 3291br (O–H), 1680br (H–O–H), 1170m (P=O), 951s (P–OH). MS (ESI^+^, MeOH) found: *m*/*z* 528.4 ([H_2_(Bn-NODP)+H]^+^).

### Synthesis of Na_2_(Bn-NODP)·5H_2_O

1-Benzyl-1,4,7-triazacyclononane (1.00 g, 4.55 mmol) was dissolved in THF (20 mL). PhP(OEt)_2_ (2.17 g, 11.0 mmol) and (CH_2_O)_*n*_ (0.33 g, 11.0 mmol) were added, and the solution was refluxed for 16 h. The solvent was removed *in vacuo*, and the phosphinate ester intermediate was obtained as a yellow oil. Then, 6 M HCl (20 mL) was added, and the solution was heated to 80 °C for 15 h. The pH was raised to 14 via addition of aqueous NaOH, and a white solid precipitated, which was isolated via filtration. This crude product was then dissolved in MeOH, dry loaded with silica, and purified using a Biotage Selekt flash chromatography system (reverse phase SFar C18 column, 0–60% MeCN/H_2_O). The clean fractions were combined and brought to dryness to yield a sticky solid that was washed twice with MeCN to yield the product as a white solid. Yield: 1.34 g, 45%. Required for C_27_H_33_N_3_Na_2_O_4_P_2_·5H_2_O (661.57): C, 49.02; H, 6.55; N, 6.35%. Found: C, 49.06; H, 6.48; N, 6.20%. ^1^H NMR (*d*_4_-MeOH/ppm): δ 7.75 (m, [4H], Ar-*H*), 7.48 (m, [2H], 7.40 (m, [9H], Ar-*H*), 4.08 (s, [2H], NC*H*_2_Ar), 3.04–2.68 (m, [16H], NC*H*_2_P and C*H*_2_ tacn). ^13^C{^1^H} NMR (*d*_4_-MeOH/ppm): δ 139.69 (d, ^1^*J*_PC_ = 123.2 Hz, *ipso*-*C*, Ph), 134.31 (*ipso*-*C*, Bn), 132.60 (d, ^3^*J*_PC_ = 9.5 Hz, *m*-*C*H, Ph), 132.15 (s, *m*-*C*H, Bn), 131.82 (d, ^4^*J*_PC_ = 2.9 Hz, *p*-*C*H, Ph), 130.34 (s, *p*-*C*H, Bn), 130.16 (s, *o*-*C*H, Bn), 129.36 (*d*, ^2^*J*_PC_ = 11.7 Hz, *o*-*C*H, Ph), 61.12 (*C*H_2_Bn), 58.59 (d,^1^*J*_PC_ = 106.4 Hz, *C*H_2_P), 55.39, 54.01 (br, 2 × CH_2_, tacn), 52.83 (CH_2_NBn, tacn). ^31^P{^1^H} (*d*_4_-MeOH): δ 24.41 (s). HRMS (ESI^+^, MeOH) found: *m*/*z* 572.1822 ([Na_2_(Bn-NODP)+H]^+^ calcd *m*/*z* 572.1820), 550.2005 ([NaH(Bn-NODP)+H]^+^ calc. *m*/*z* 550.2001), 528.2183 ([H_2_([Bn-NODP)+H]]^+^ calcd *m*/*z* 528.2181).

### Synthesis of H_2_(Bn-NODP)

1-Benzyl-1,4,7-triazacyclononane (2.00 g, 9.1 mmol) was dissolved in THF (20 mL). PhP(OMe)_2_ (3.72 g, 21.9 mmol) and (CH_2_O)_*n*_ (0.66 g, 21.9 mmol) were added, and the solution was refluxed for 16 h. The solvent was removed *in vacuo*, and the phosphinate ester intermediate was obtained as a yellow oil. Then, 6 M HCl (20 mL) was added, and the solution was heated to 80 °C for 15 h under N_2_. Solvent was removed *in vacuo* and the residue stirred in Et_3_N (20 mL) for 30 min, then the solvent was removed *in vacuo*. The residue was then purified using a Biotage Selekt flash chromatography system (reverse phase SFar C18 column, 0–60% MeCN/H_2_O) to yield the desired product as a hydroscopic white solid. ^1^H NMR (*d*_4_-MeOH/ppm): δ 7.83 (m, [4H], Ar-*H*), 7.63 (m, [2H], 7.46 (m, [9H], Ar-*H*), 4.39 (s, [2H], NC*H*_2_Ar), 3.11–2.77 (m, [16H], NC*H*_2_P and C*H*_2_ tacn). ^13^C{^1^H} NMR (*d*_4_-MeOH/ppm): δ 137.03 (d,^1^*J*_PC_ = 126.2 Hz, *ipso*-*C*, Ph), 133.10 (s, *ipso*-C, Bn), 132.64 (br, overlapping *p*-*C*H, Ph and *m*-*C*H, Bn), 132.56 (d, ^3^*J*_PC_ = 9.4 Hz, *m*-*C*H, Ph), 130.75 (s, *p*-*C*H, Bn), 130.31 (s, *o-C*H, Bn), 129.59 (d, ^2^*J*_PC_ = 11.8 Hz, *o*-*C*H, Ph), 60.55 (s, *C*H_2_Bn), 58.36 (d, ^1^*J*_PC_ = 107.8 Hz, *C*H_2_P), 54.69 (br d, 2 × *C*H_2_, tacn), 52.35 (s, *C*H_2_NBn, tacn). ^31^P{^1^H} (*d*_4_-MeOH/ppm): δ 27.21 (s). MS (ESI^+^, MeOH) found: *m*/*z* 528.4 ([H_2_(Bn-NODP)+H]^+^).

### Synthesis of [GaCl(Bn-NODP)]·*x*H_2_O

#### Method 1

Ga(NO_3_)_3_·9H_2_O (0.063 g, 0.15 mmol) was dissolved in water (2 mL), and [Na_2_(Bn-NODP)]·5H_2_O (0.100 g, 0.15 mmol) in water (1 mL) was added to the solution. The solution was stirred for 30 min. To the remaining solution was added 6 M HCl (0.2 mL), causing a white precipitate to form. This was isolated via filtration and dried *in vacuo.* Yield: 0.078 g, 74%. Required for C_27_H_33_ClGaN_3_O_4_P_2_·4H_2_O (702.25): C, 46.15; H, 5.88; N, 5.98%. Found: C, 45.38; H, 5.09; N, 5.68%. ^1^H NMR (*d*_4_-MeOH), ppm): δ 8.20–8.07 (m, [4H]), 7.68–7.41 (m, [11H], Ar-*H*), 4.68 (d, ^2^*J*_HH_ = 14.2 Hz, [1H], NC*H*_2_Bn), 4.09 (d, ^2^*J*_HH_ = 14.2 Hz, [1H], NC*H*_2_Bn), 3.90 (td, ^2^*J*_HH_ = 13.3 Hz, ^2^*J*_HP_ = 6.2 Hz, [1H], PC*H*_2_N), 3.80 (td, ^2^*J*_HH_ = 12.8 Hz, ^2^*J*_HP_ = 6.4 Hz, [1H], PC*H*_2_N), 3.66–3.14 (m, [12H], PC*H*_2_N and C*H*_2_ tacn), 2.97 (m, [1H], C*H*_2_, tacn), 2.54 (dd, [1H], ^2^*J*_HH_ = 13.0 Hz, ^4^*J*_HP_ = 5.0 Hz, C*H*_2_, tacn). ^31^P{^1^H} (*d*_4_-MeOH, ppm): δ 29.41 (s, [1P]), 26.51 (s, [1P]). ^71^Ga NMR (*d*_4_-MeOH, ppm): not observed. MS (ESI^+^, MeOH) found: *m*/*z* 632.4 ([GaCl(Bn-NODP)+H]^+^).

#### Method 2

Ga(NO_3_)_3_·9H_2_O (0.124 g, 0.30 mmol) was dissolved in MeOH (5 mL), and [H_2_(Bn-NODP)]·2HCl·4H_2_O (0.200 g, 0.30 mmol) was dissolved in MeOH (2 mL) and added to the solution. The solution was stirred for 3 h. The pale-yellow solution was filtered then brought to dryness *in vacuo*. The sticky solid was dissolved in MeCN (2 mL), filtered, and layered with Et_2_O (3 mL). A white solid was collected via filtration and dried *in vacuo.* Yield: 0.134 g, 64%. ^1^H NMR (*d*_4_-MeOH), ppm): δ 8.19–8.06 (m, [4H]), 7.68–7.41 (m, [11H], Ar-*H*), 4.68 (d, ^2^*J*_HH_ = 14.2 Hz, [1H], NC*H*_2_Bn), 4.09 (d, ^2^*J*_HH_ = 13.9 Hz, [1H], NC*H*_2_Bn), 3.90 (td, ^2^*J*_HH_ = 13.3 Hz, ^2^*J*_HP_ = 6.1 Hz, [1H], PC*H*_2_N), 3.80 (td, ^2^*J*_HH_ = 13.3 Hz, ^2^*J*_HP_ = 6.1 Hz, [1H], PC*H*_2_N), 3.67–3.15 (m, [12H], PC*H*_2_N and C*H*_2_ tacn), 2.97 (m, [1H], C*H*_2_, tacn), 2.54 (dd, [1H], ^2^*J*_HH_ = 13.0 Hz, ^4^*J*_HP_ = 5.1 Hz, C*H*_2_ tacn). ^31^P{^1^H} (*d*_4_-MeOH, ppm): δ 31.02 (s, [1P]), 29.71 (s, [1P]). MS (ESI^+^, MeOH) found: *m*/*z* 632.4 ([GaCl(Bn-NODP)+H]^+^). IR (Nujol/cm^–1^): 3482 br (O–H), 1634br (H–O–H), 376s (Ga–Cl).

#### Method 3

GaCl_3_ (0.026 g, 0.15 mmol) was dissolved in water (2 mL), and [Na_2_(Bn-NODP)]·5H_2_O (0.100 g, 0.15 mmol) in water (1 mL) was added to the solution. The solution was stirred for 30 min. Then, 6 M HCl (0.2 mL) was added, causing a white precipitate to form. This was isolated via filtration and dried *in vacuo.* Yield: 0.055 g, 52%. ^1^H NMR (*d*_4_-MeOH), ppm): δ 8.19–8.07 (m, [4H]), 7.65–7.41 (m, [11H], Ar-*H*), 4.68 (d, ^2^*J*_HH_ = 14.2 Hz, [1H], NC*H*_2_Bn), 4.09 (d, ^2^*J*_HH_ = 14.3 Hz, [1H], NC*H*_2_Bn), 3.90 (td, ^2^*J*_HH_ = 13.3 Hz, ^2^*J*_HP_ = 6.1 Hz, [1H], PC*H*_2_N), 3.87 (td, ^2^*J*_HH_ = 13.0 Hz, ^2^*J*_HP_ = 5.8 Hz, [1H], PC*H*_2_N), 3.65–3.12 (m, [12H], PC*H*_2_N and C*H*_2_ tacn), 2.94 (m, [1H], C*H*_2_, tacn), 2.52 (dd, [1H], ^2^*J*_HH_ = 13.1 Hz, ^4^*J*_HP_ = 5.0 Hz, C*H*_2_, tacn). ^31^P{^1^H} (*d*_4_-MeOH, ppm): δ 27.58 (s, [1P]), 26.08 (s, [1P]). MS (ESI^+^, MeOH) found: *m*/*z* 632.4 ([GaCl(Bn-NODP)+H]^+^).

#### Method 4

GaCl_3_ (0.026 g, 0.15 mmol) was dissolved in water (2 mL), and [H_2_(Bn-NODP)]·2HCl·4H_2_O (0.100 g, 0.15 mmol) in water (1 mL) was added to the solution. The solution was stirred for 30 min. Then, 6 M HCl (0.2 mL) was added, causing a white precipitate to form. This was isolated via filtration and dried *in vacuo.* Yield: 0.072 g, 68%. ^1^H NMR (*d*_4_-MeOH), ppm): δ 8.22–8.05 (m, [4H]), 7.68–7.37 (m, [11H], Ar-*H*), 4.68 (d, ^2^*J*_HH_ = 14.3 Hz, [1H], NC*H*_2_Bn), 4.09 (d, ^2^*J*_HH_ = 13.8 Hz, [1H], NC*H*_2_Bn), 3.90 (td, ^2^*J*_HH_ = 13.3 Hz, ^2^*J*_HP_ = 5.9 Hz, [1H], PC*H*_2_N), 3.86 (td, ^2^*J*_HH_ = 13.3 Hz, ^2^*J*_HP_ = 6.2 Hz, [1H], PC*H*_2_N), 3.65–3.11 (m, [12H], PC*H*_2_N and C*H*_2_, tacn), 2.94 (m, [1H], C*H*_2_, tacn), 2.52 (dd, [1H], ^2^*J*_HH_ = 13.0 Hz, ^4^*J*_HP_ = 5.0 Hz, C*H*_2_, tacn). ^31^P{^1^H} (*d*_4_-MeOH, ppm): δ 27.72 (s, [1P]), 26.11 (s, [1P]). MS (ESI^+^, MeOH) found: *m*/*z* 632.4 ([GaCl(Bn-NODP)+H]^+^).

### Synthesis of [GaF(Bn-NODP)]·4H_2_O

[GaCl(Bn-NODP)]·4H_2_O (0.100 g, 0.14 mmol) was dissolved in MeOH (2 mL), KF (0.008 g, 0.14 mmol) was added and the solution refluxed for 4 h. The solution was brought to dryness *in vacuo*. The sticky solid was dissolved in MeCN (2 mL), filtered, and layered with Et_2_O (3 mL). A white solid was collected via filtration and dried *in vacuo.* Yield: 0.065 g, 68%. Required for C_27_H_33_FGaN_3_O_4_P_2_·4H_2_O (686.30): C, 47.25; H, 6.02; N, 6.12%. Found: C, 47.58; H, 5.49; N, 5.82%. ^1^H NMR (*d*_4_-MeOH), ppm): δ 8.19–8.07 (m, [4H]), 7.65–7.40 (m, [11H], Ar-*H*), 4.68 (d, ^2^*J*_HH_ = 14.3 Hz, [1H], NC*H*_2_Bn), 4.09 (d, ^2^*J*_HH_ = 14.1 Hz, [1H], NC*H*_2_Bn), 3.90 (td, ^2^*J*_HH_ = 13.3 Hz, ^2^*J*_HP_ = 6.1 Hz, [1H], PC*H*_2_N), 3.87 (td, ^2^*J*_HH_ = 13.0 Hz, ^2^*J*_HP_ = 5.9 Hz, [1H], PC*H*_2_N), 3.68–3.10 (m, [12H], PC*H*_2_N and C*H*_2_ tacn), 2.93 (m, [1H], C*H*_2_, tacn), 2.52 (dd, [1H], ^2^*J*_HH_ = 12.9 Hz, ^4^*J*_HP_ = 5.1 Hz, C*H*_2_, tacn). ^31^P{^1^H} NMR (*d*_4_-MeOH, ppm): δ 26.96 (d, ^3^*J*_PF_ = 3.0 Hz, [1P]), 26.49 (s, [1P]). ^19^F{^1^H} NMR (*d*_4_-MeOH, ppm): δ −176.1 (br s). ^71^Ga NMR (*d*_4_-MeOH, ppm): not observed. IR data (Nujol, ν/cm^–1^): 3649 br (O–H), 1670 br (H–O–H), 585s (Ga–F). MS (ESI^+^ MeOH) found: *m*/*z* 614.3 ([GaF(Bn-NODP)+H]^+^. Single crystals were obtained from an aqueous solution of [GaF(Bn-NODP)]·4H_2_O via slow evaporation over a few days.

### Synthesis of [FeCl(Bn-NODP)]·3H_2_O

FeCl_3_ (0.057 g, 0.35 mmol) was dissolved in water (3 mL). H_2_(Bn-NODP)·2HCl·4H_2_O (0.200 g, 0.30 mmol) was dissolved in water (2 mL) and added, and the solution was heated to 80 °C for 4 h. Then, 6 M HCl (1 mL) was added, and the solution was stirred for 30 min to form a yellow precipitate, which was isolated via filtration and dried *in vacuo*. Yield: 0.061 g, 31%. Required for C_27_H_33_ClFeN_3_O_4_P_2_·3H_2_O (661.86): C, 48.34; H, 5.86; N, 6.26%. Found: C, 48.35; H, 5.26; N, 5.66%. IR data (Nujol, ν/cm^–1^): 3425 br (O–H), 1651 br (H–O–H), 383s Fe–Cl. MS (ESI^+^ MeOH) found: *m*/*z* 617.2 ([FeCl(Bn-NODP)+H^+^]).

### Synthesis of [FeF(Bn-NODP)]·5H_2_O

[FeCl(Bn-NODP)]·3H_2_O (0.050 g, 0.08 mmol) was dissolved in MeOH (3 mL), and KF (0.005 g, 0.08 mmol) was added. The solution was refluxed for 3 h. The colorless solution was filtered to remove any inorganic particulates, and the supernatant was then reduced to dryness, redissolved in MeCN (1 mL), filtered, and brought to dryness *in vacuo*, leaving a white solid. Yield: 0.020 g, 36%. Required for C_27_H_33_FFeN_3_O_4_P_2_·5H_2_O (690.43): C, 46.97; H, 6.28; N, 6.09%. Found: C, 47.16; H, 5.55; N, 5.95%. IR data (Nujol, ν/cm^–1^): 3395 br (O–H), 1649 br (H–O–H), 575s Fe–F. MS (ESI^+^ MeOH) found: *m*/*z* 601.2 ([FeF(Bn-NODP)+H^+^]).

### X-ray Crystallography

Single crystals were grown as described above, and crystallographic parameters are summarized in Table S1. Data collection used a Rigaku AFC12 goniometer equipped with an enhanced-sensitivity (HG) Saturn724^+^ detector mounted at the window of an FR-E^+^ SuperBright molybdenum (λ = 0.71073 Å) rotating anode generator with VHF or HF Varimax optics (70 or 100 μm focus), with the crystal held at 100 K (N_2_ Cryostream). Structure solution and refinement were performed using SHELX (T)-2018/2 and SHELX-2018/3 through Olex2^19−21^ and were mostly straightforward, except for the Na_2_(Bn-NODP)·4H_2_O crystal, in which there was one disordered water molecule, successfully modeled at 0.33:0.67 occupancy. The H atoms associated with the lattice water molecules were clearly evident in the difference map, and all H atoms were added and refined with a riding model. Where additional restraints were required, details are provided in the cif file. CCDC reference numbers for the crystallographic information files in cif format are 2290361 ([{H(Bn-NODP)Na(H_2_O)_2_}_2_(μ-OH_2_)]·6H_2_O), 2290362 ([GaF(Bn-NODP)]·4H_2_O), and 2290363 ([H_2_(Bn-NODP)] ·2HCl·H_2_O).

### Computational Details

Density functional theory (DFT) calculations were carried out using the B3LYP^22^ and BP86^23^ functionals, augmented with the Grimme correction for dispersion (D3(BJ) version),^24^ using 6-311G(d,p) basis sets with Gaussian 16.^25^ Overall, the results obtained with the two functionals showed reasonably good agreement. Geometry optimizations were performed for each structure investigated, and energy minima were confirmed by the absence of any imaginary frequencies. In order to investigate the number, nature, and strength of the H-bonding interactions in each structure, the outputs from the geometry optimization calculations were used in AIM (Atoms in Molecules) calculations with Gaussian 16 and MULTIWFN.^26^ AIM theory^27^ is based upon a topological analysis of the electron density ρ(*r*) of a molecular system. It identifies a bond critical point (BCP) in a bond, which is a saddle point along the gradient path ∇ρ(*r*) connecting two local electron ρ(*r*) density maxima. At the BCPs, from the values of ρ(*r*) and ∇^2^ρ(*r*), it is possible to determine the nature of a chemical bond. In hydrogen bonds (HBs), ρ(*r*) and ∇^2^ρ(*r*) at the BCP should be both small and positive and are typically in the range 0.002–0.035 au and 0.02–0.15 au, respectively.^28^ The energy of each HB identified was calculated in this work using the approximate formula^29^
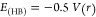
1where *V*(*r*) is the local potential energy density value at each BCP.

As discussed above, the [GaX(Bn-NODP)] complexes, where X = F or Cl, can exist as two geometric isomers (isomer 1 and isomer 2 in Figure 3), and the chirality at P leads to four stereoisomers for isomer 1 (*RR*, *SR*, *RS*, *SR*) and three stereoisomers (*RR*, *SS*, *SR*) for isomer 2 (*SR* and *RS* are the same for this isomer). Most calculations were performed for X = F, and some were carried out for X = Cl. The crystal structure of [GaF(Bn-NODP)]·4H_2_O, which adopts the isomer 1/*RR* form (Figure 5), provides input coordinates for the calculations and also indicates that hydrogen bonding, via O···H, F···H, and C···H interactions may contribute to the relative energy of the seven forms. This was investigated for each isomer by determining the number of HBs (hydrogen bonds), calculating *E*_(HB)_ for each HB using eq 1 and calculating the total HB energy, using AIM calculations.

### Radiofluorination Procedures

In a typical experiment, [GaCl(Bn-NODP)]·4H_2_O (1 mg, 1.42 μmol or 0.1 mg, 142 nmol) was dissolved in MeCN (0.3 mL) and 1 M NaOAc_aq_ buffer (0.45 mL), or for [FeCl(Bn-NODP)]·3H_2_O (1 mg, 1.51 μmol or 0.1 mg, 151 nmol), dissolved in EtOH (0.75 mL). To this solution, 0.25 mL of an aqueous solution containing [^18^F]F^–^ (80–200 MBq) was added and heated to 80 °C for 10 min. An aliquot (∼100 μL) of the crude reaction solution was diluted with water (900 μL) so that approximately 10% of the solvent composition was organic. The sample of diluted crude reaction solution was analyzed by analytical radio-HPLC, which confirmed the percentage incorporation of [^18^F]F^–^ into the metal complex (based upon integration of the radio peaks). The products were purified using SPE purification.

### SPE Purification Protocol

The crude reaction mixture was diluted with 3 mL of water and was trapped on an HLB (hydrophobic-lipophilic-balance) cartridge (Waters, P/N 186000132) and washed with water (10 mL) to remove [^18^F]F^–^, and then the product was eluted from the cartridge with ethanol (2 mL) into either (i) water to result in a formulated product in 90:10 H_2_O/EtOH or (ii) PBS (PBS solution prepared by dissolving one oxoid phosphate buffered saline tablet in 100 mL of deionized water), to result in a formulated product in 90:10 PBS/EtOH. The formulated product was analyzed by HPLC at *t* = 0 and various time intervals up to 4 h. Experiments were analyzed on an Agilent 1290 HPLC system with an Agilent 1260 DAD UV detector (G4212B) and a Bioscan FC3200 sodium iodide PMT with a rate meter. Dionex Chromeleon 6.8 Chromatography data recording software was used to integrate the peak areas.

### Analytical HPLC method

Column: Phenomenex Luna 5 μm C18(2) 250 × 4.6 mm. Mobile phase A = water, B = MeCN. Flow rate 1 mL min^–1^. Gradient 0–13 min (0–100% B).

## Conclusions and Outlook

Previous work has demonstrated that Al–^18^F complexes with NOTA- and NODA-derived chelates show great promise for new radiopharmaceuticals for PET imaging. However, with the advent of total body PET, it is timely to broaden the range of potential PET tracers. Earlier studies showed that, while [Ga^18^F(Bn-NODA)] is formed readily in partially aqueous solutions and shows good radiochemical stability over several hours in aqueous EtOH, rapid loss of fluoride has been observed at close to physiological pH (7.4).^9b^

In this work, we have developed a new dianionic, pentadentate bis(phosphinate) chelator, Bn-NODP, and demonstrated that it binds effectively to Ga(III) and Fe(III) ions, with a chloride ligand completing the distorted octahedral coordination sphere at the metal. Addition of aqueous fluoride (KF) results in rapid Cl/F ligand exchange, driven by the higher thermodynamic stability resulting from the formation of a strong M–F bond. Solution spectroscopic data on the gallium species, as well as X-ray crystal analysis of [GaF(Bn-NODP)]·4H_2_O, confirm that all of the complexes adopt the asymmetric geometric isomer, isomer 1, exclusively, and the crystal structure shows the *RR* stereoisomer. DFT calculations predict that isomer 1 *RR* is likely to be more stable than the other isomers, as observed experimentally.

Finally, we have demonstrated that both of the [MCl(Bn-NODP)] complexes undergo fast radiofluorination with ^18^F^–^ in partially aqueous solvents with brief (10 min) heating at 80 °C, giving very promising radiochemical yields. Furthermore, we have shown that the resulting [Ga^18^F(Bn-NODP)] radio product has excellent radiochemical stability when formulated in 90:10 H_2_O/EtOH and in 90:10 PBS/EtOH at pH 7.4 over 3.5 h. The significantly increased stability observed for [Ga^18^F(Bn-NODP)] over the previously reported [Ga^18^F(Bn-NODA)]^9b^ is likely to be at least in part due to the wider chelate bite angles (by 3–4°) present in the phosphinate derivatives, causing less strain in the complexes and leading to greater stability against hydrolysis and chelate ring-opening at higher pH. The lower stability of the [Fe^18^F(Bn-NODP)] radioproduct over time may be associated with the lower bond strength of Fe–F compared to Ga–F.

Our future work will explore complexes of Bn-NODP variants with other metal ions (Al, Sc), the effect of the P-bound substituent on the lipophilicity, as well as incorporation of linkers to facilitate bioconjugation and biological studies to evaluate these species further for PET imaging applications.

## Data Availability

doi.org/10.5258/SOTON/D2767 contains the *x*,*y*,*z* coordinates for the DFT calculations reported in this work.

## Supporting Information Available

Multinuclear NMR, IR, and mass spectra associated with the new compounds described; additional figures showing the H-bonding networks present in the crystal structures, together with the computational details from the DFT calculations and additional radiotraces associated with the work reported (PDF)
