# Correction to “Synthesis, Characterization, and Computational Studies on Gallium(III) and Iron(III) Complexes with a Pentadentate Macrocyclic *bis*-Phosphinate Chelator and Their Investigation As Molecular Scaffolds for ^18^F Binding”

**DOI:** 10.1021/acs.inorgchem.3c04499

**Published:** 2024-01-18

**Authors:** Danielle E. Runacres, Victoria K. Greenacre, John M. Dyke, Julian Grigg, George Herbert, William Levason, Graeme McRobbie, Gillian Reid

The concentrations of the solutions used for the ^18^F-radiolabeling experiments are incorrect because the ×1000 conversion from milliliters to liters was not made. This occurs in five places as follows:

Abstract, line 25: “or ∼1.5 μM” should read “or ∼1.4 mM”.

Page 20845, right column, line 20: “nM” should read “μM”.

Page 20850, right column, line 17: “μM” should read “mM”.

Page 20850, right column, line 18: “nM” should read “μM”.

Page 20851, Table 4, column 3, heading: “nm” should read “μM”.

