# Supplementary material for: Synthesis, Characterization, and Computational Studies on Gallium(III) and Iron(III) Complexes with a Pentadentate Macrocyclic bis-Phosphinate Chelator and Their Investigation As Molecular Scaffolds for 18F Binding
Source: Inorg Chem. 2023 Dec 6;62(50):20844–57. doi: 10.1021/acs.inorgchem.3c03135 (PMC10731642; doi:10.1021/acs.inorgchem.3c03135)
Supplement: Supplementary file 1 — ic3c03135_si_001.pdf [file ic3c03135_si_001.pdf]

Supporting information for:

Synthesis, characterization and computational studies on gallium(III) and iron(III) complexes with a pentadentate macrocyclic *bis*-phosphinate chelator and their investigation as molecular scaffolds for ^{18}F binding

Danielle E. Runacres[a], Victoria K. Greenacre[a], John M. Dyke[a], Julian Grigg[b], George Herbert[b], William Levason[a], Graeme McRobbie[b] and Gillian Reid[a]*

a. *School of Chemistry, University of Southampton, Southampton SO17 1BJ, UK; email: G.Reid@soton.ac.uk*

b. *GE HealthCare, Pollards Wood, Nightingales Lane, Chalfont St Giles, Bucks, HP8 4SP, UK*

Figure S1: Spectroscopic data for H_2(Bn-NODP)·2HCl

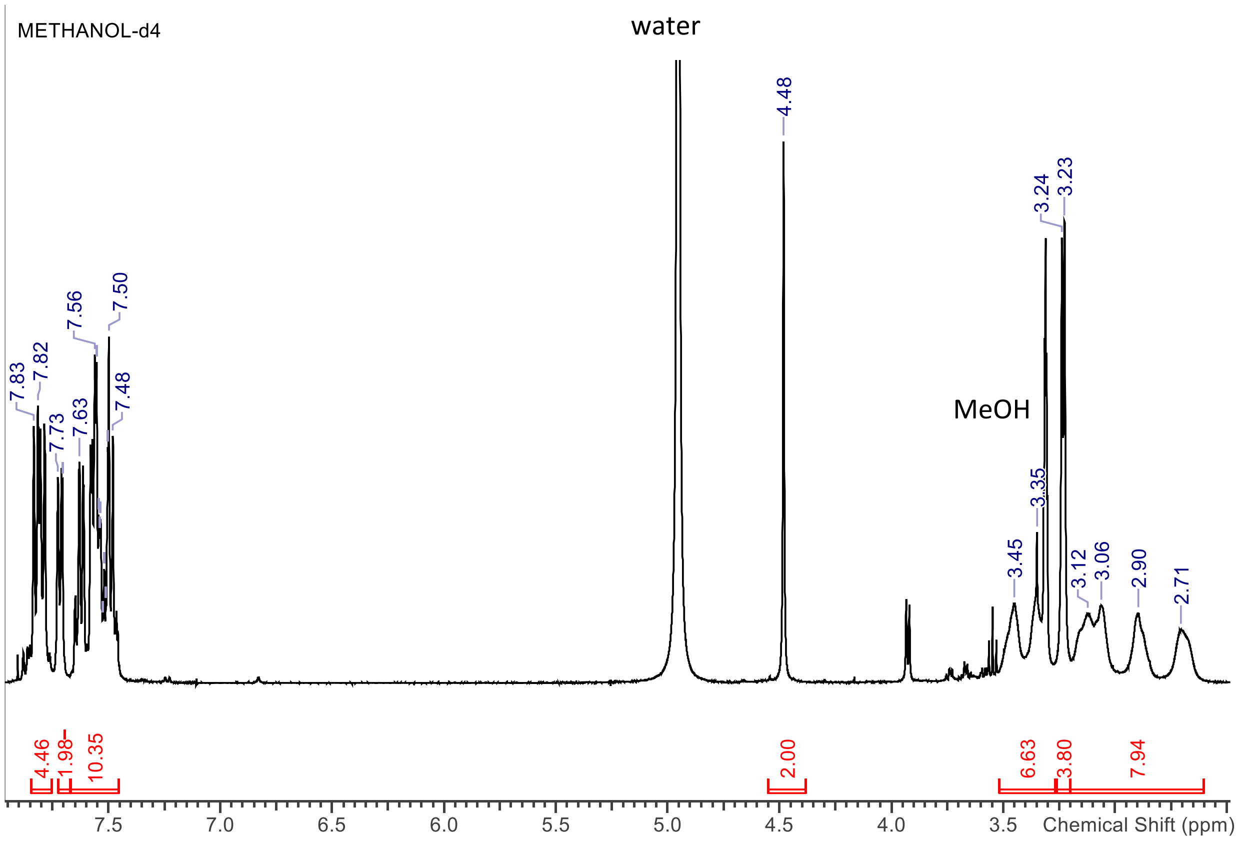

a) ^{1}H NMR: H_2(Bn-NODP)·2HCl in d4-MeOH

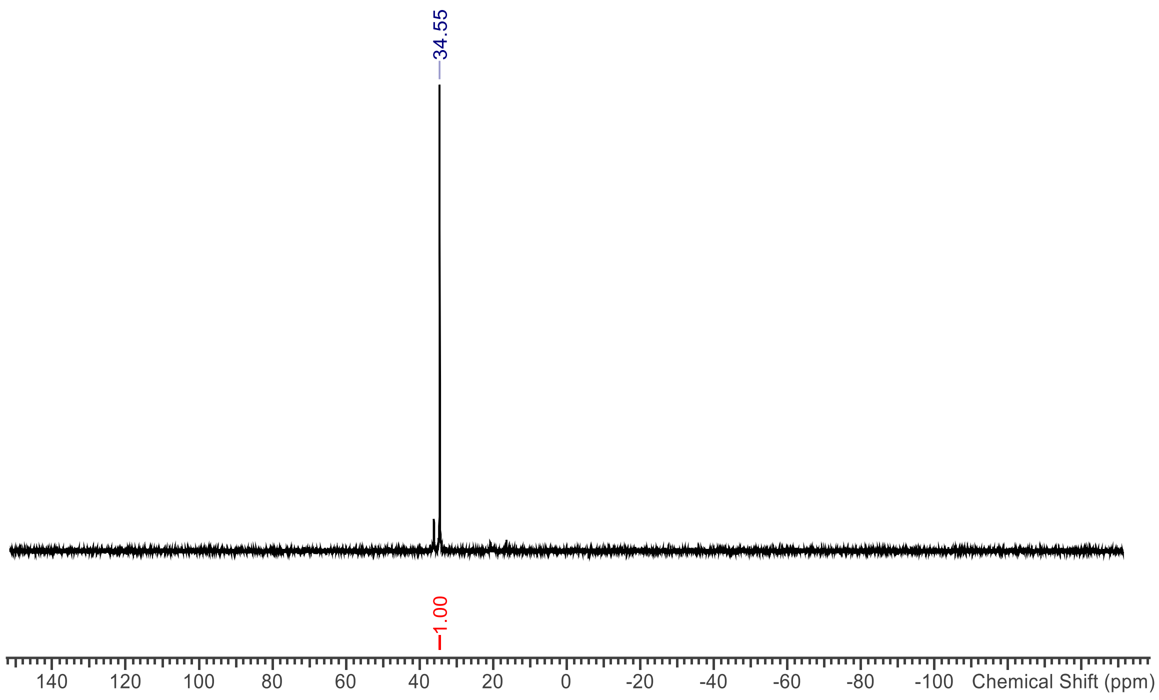

b) $^{31}P\{^{1}H\}$ NMR: H_2(Bn-NODP)·2HCl in d4-MeOH

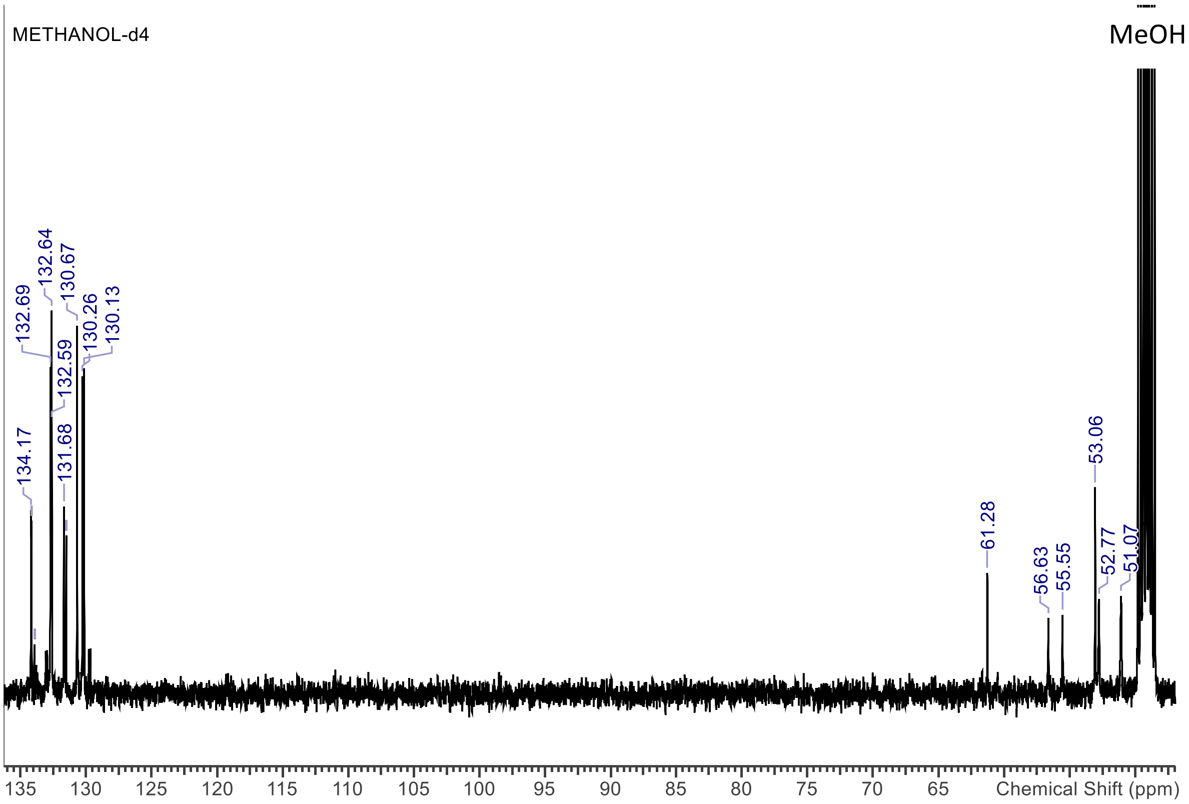

c) $^{13}C\{^{1}H\}$ NMR: H_2(Bn-NODP)·2HCl in d4-MeOH

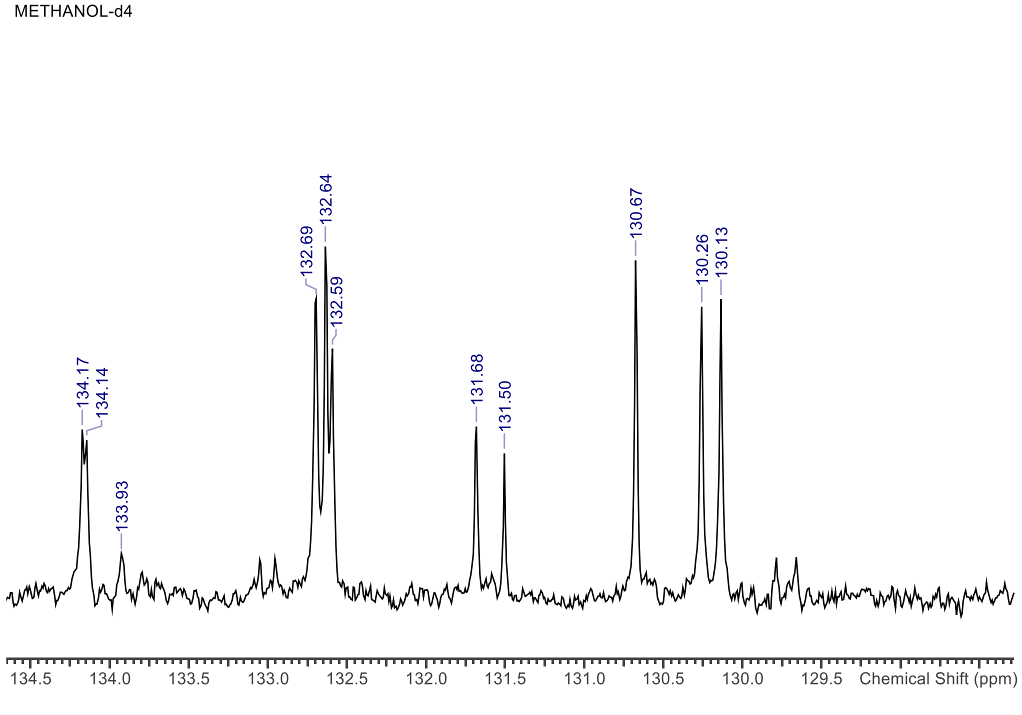

d) $^{13}C\{^{1}H\}$ NMR: H_2(Bn-NODP)·2HCl in d4-MeOH (expansion of aromatic region)

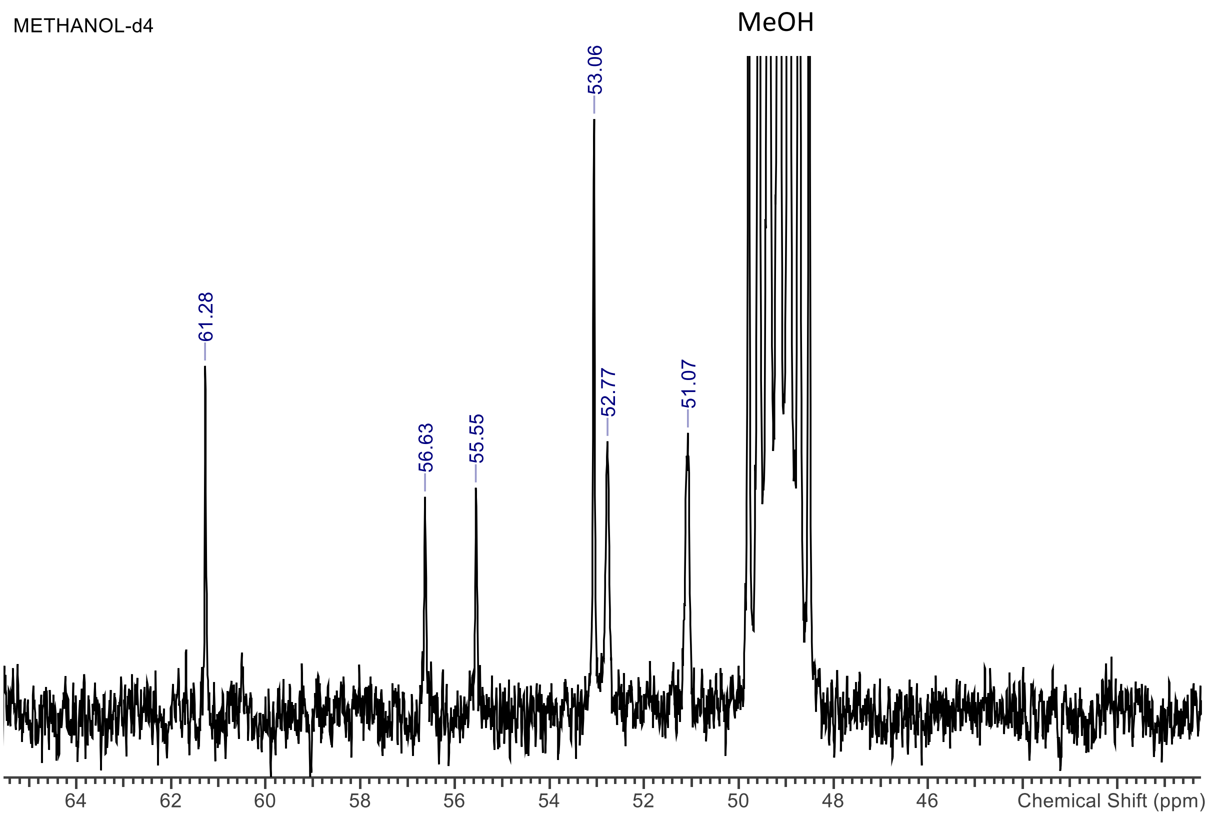

e) $^{13}C\{^1H\}$ NMR: H_2(Bn-NODP)·2HCl in d4-MeOH (expansion of aliphatic region)

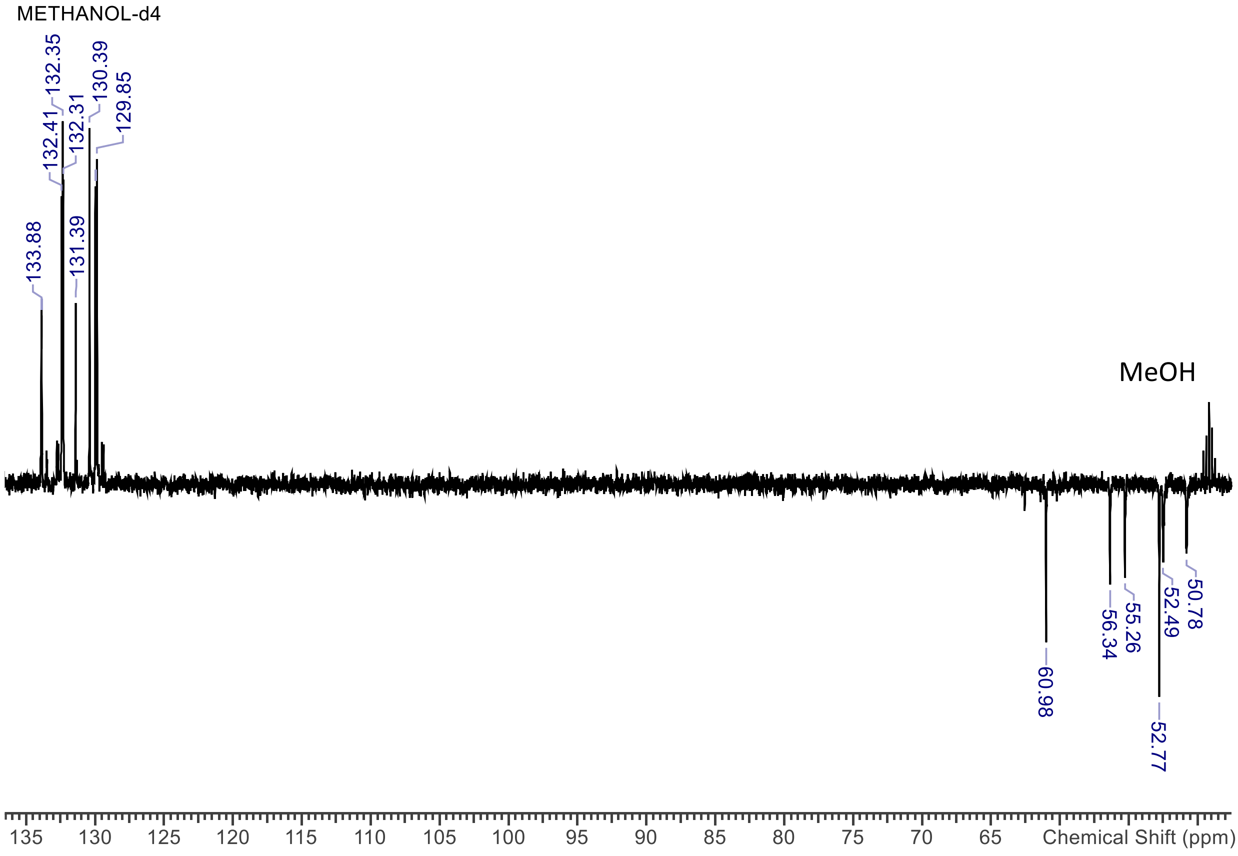

f) ^{13}C 135-dept NMR: H_2(Bn-NODP)·2HCl in d4-MeOH

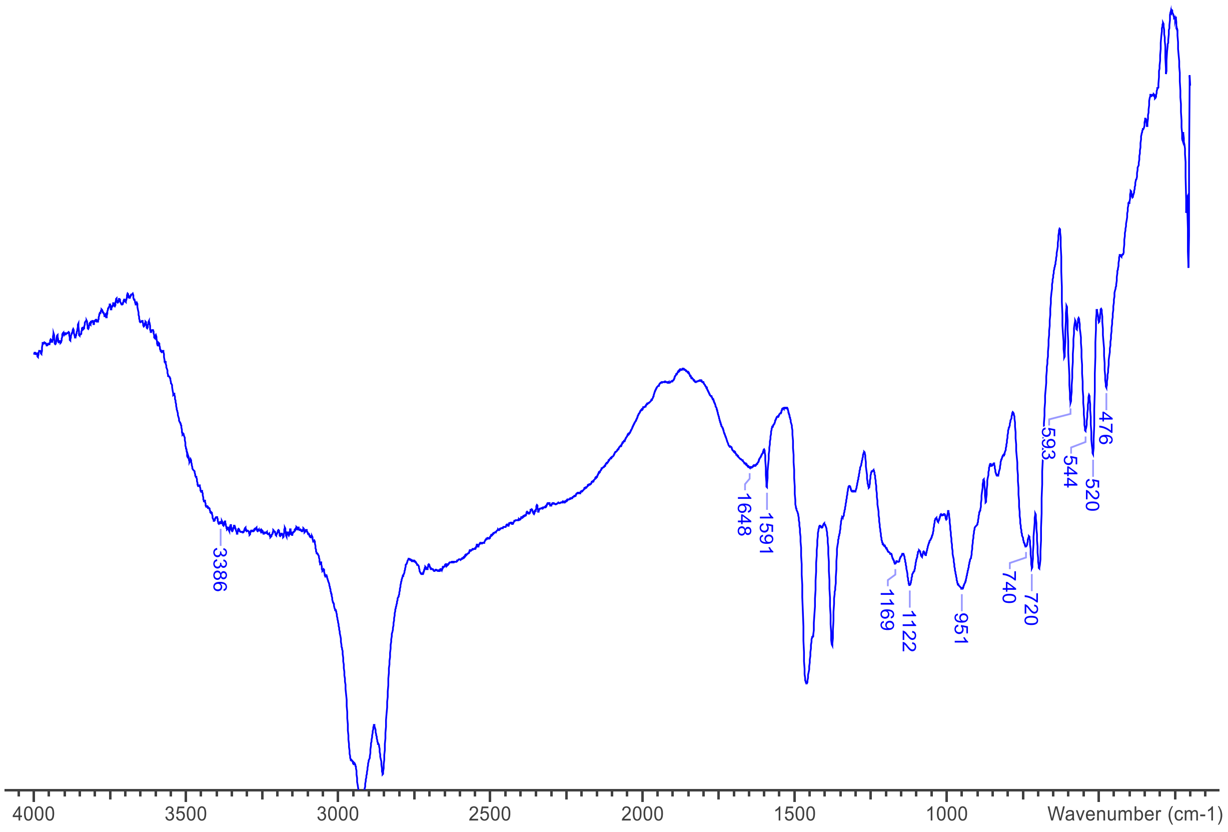

g) IR spectrum (Nujol mull) of H_2(Bn-NODP)·2HCl

Figure S2 Spectroscopic data for H_2(Bn-NODP)

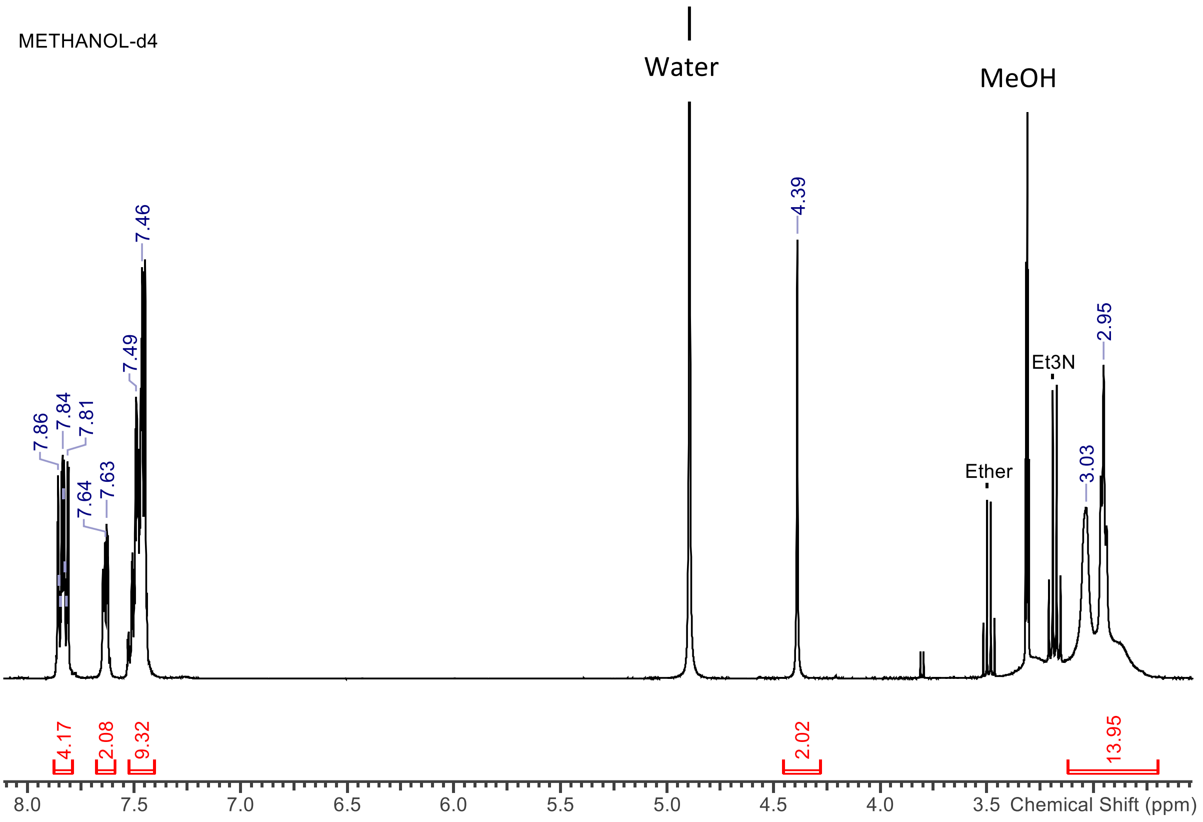

a) ^{1}H NMR: H_2(Bn-NODP) in d4-MeOH

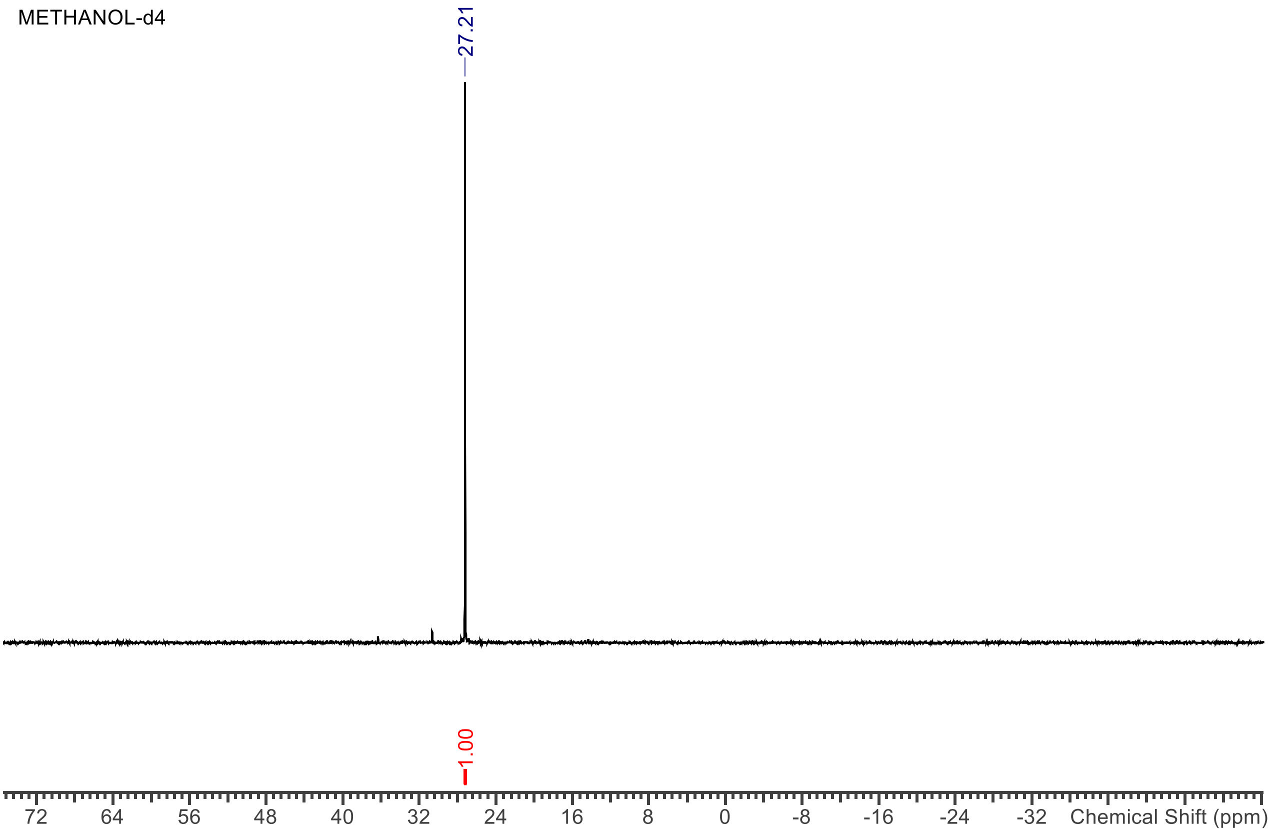

b) $^{31}P\{^1H\}$ NMR: H_2(Bn-NODP) in d4-MeOH

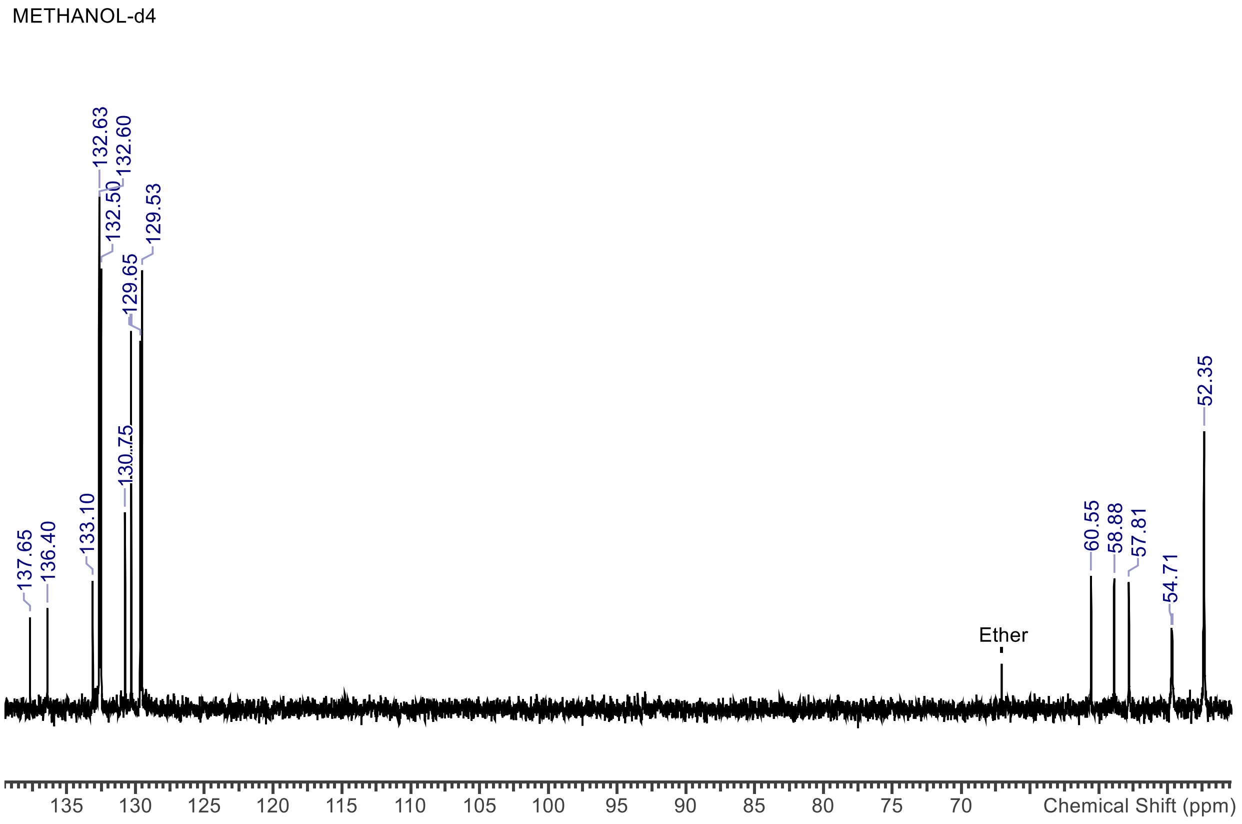

c) $^{13}C\{^{1}H\}$ NMR: H_2(Bn-NODP) in d_4-MeOH

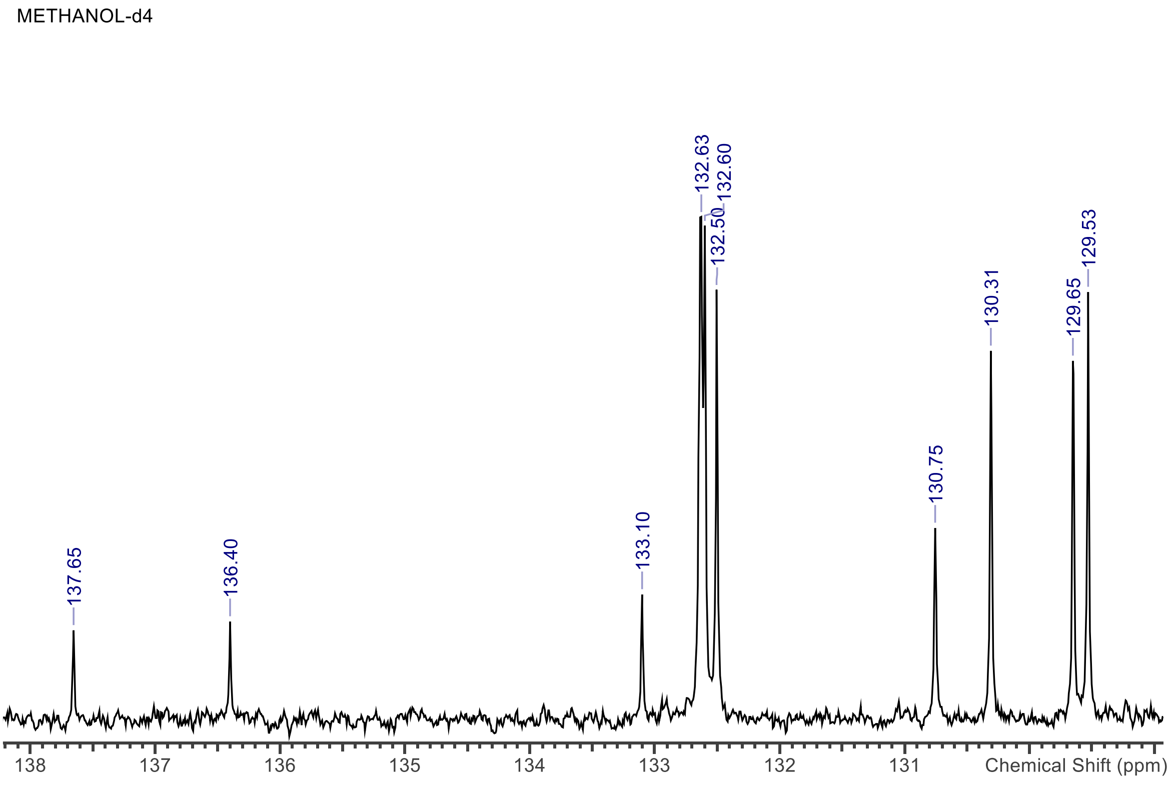

d) $^{13}C\{^{1}H\}$ NMR: H_2(Bn-NODP) in d4-MeOH (expansion of the aromatic region)

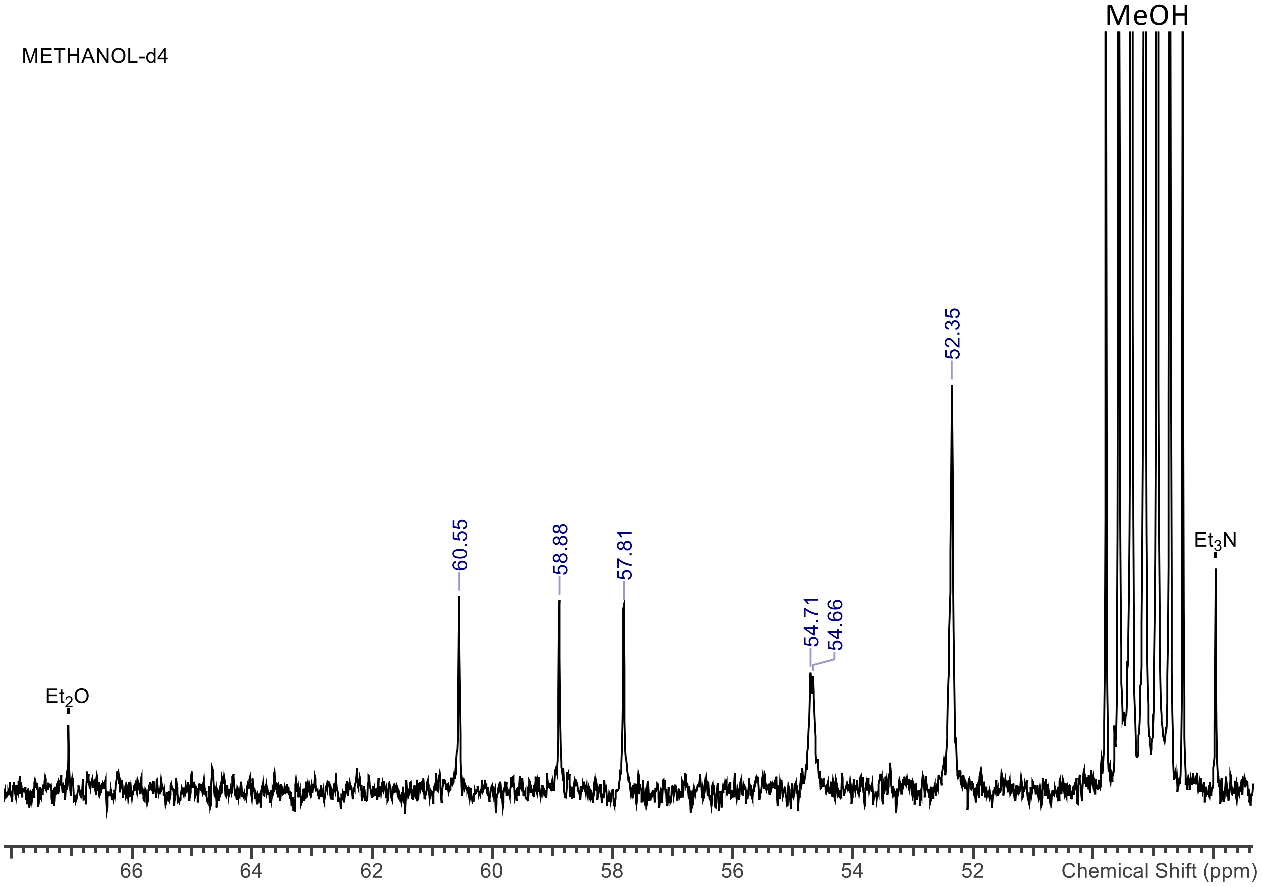

e) $^{13}C\{^{1}H\}$ NMR: [H_2(Bn-NODP)] in d4-MeOH (expansion of aliphatic region)

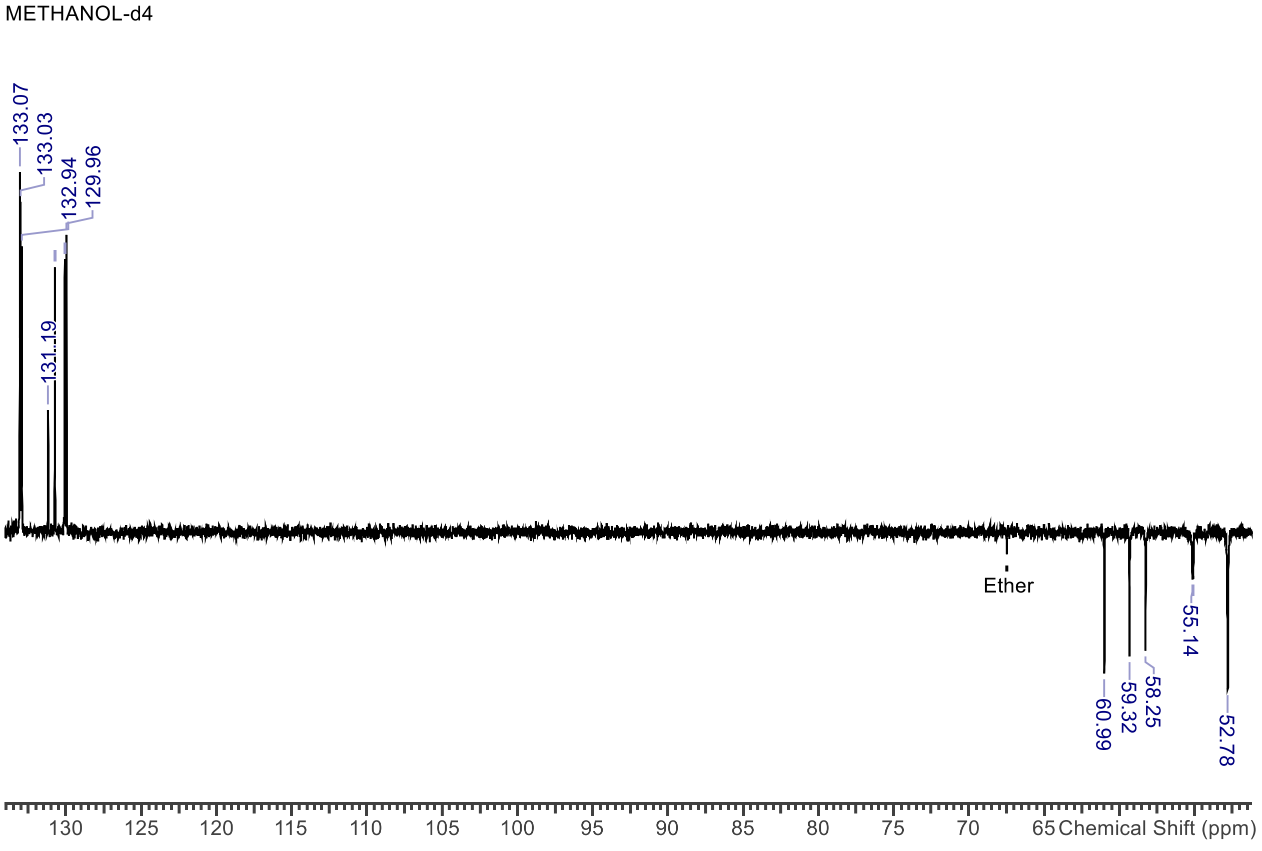

f) ^{13}C DEPT-135 NMR: H_2(Bn-NODP) in d_4-MeOH

Figure S3 Spectroscopic data for Na_2(Bn-NODP)

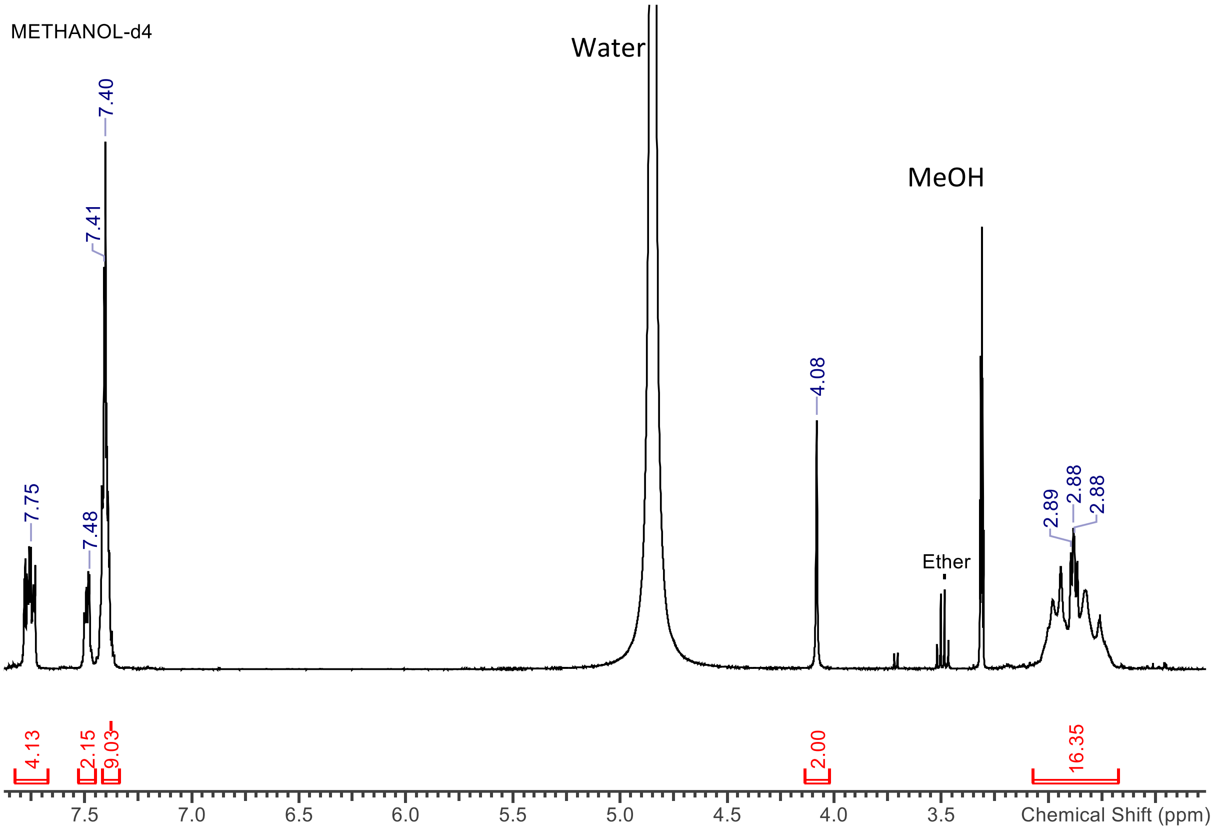

a) ^{1}H NMR: Na_2(Bn-NODP) in d_4-MeOH

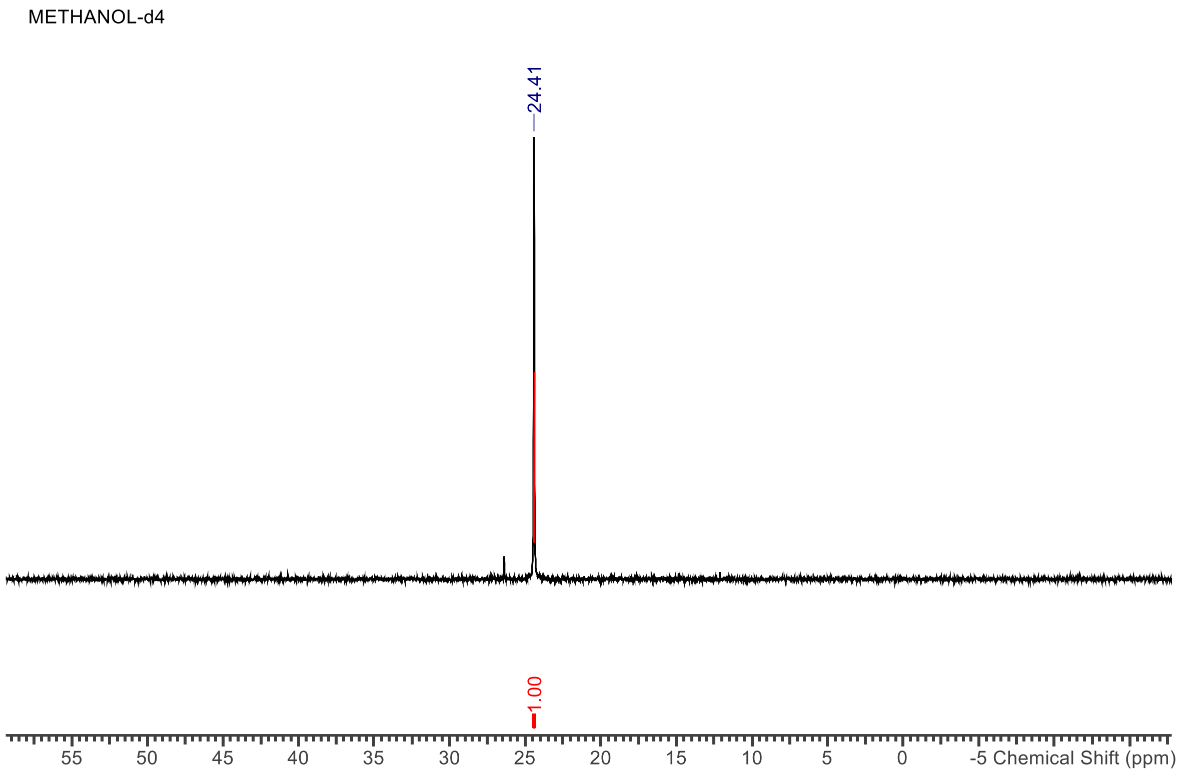

b) $^{31}P\{^{1}H\}$ NMR: Na_2(Bn-NODP) in d_4-MeOH

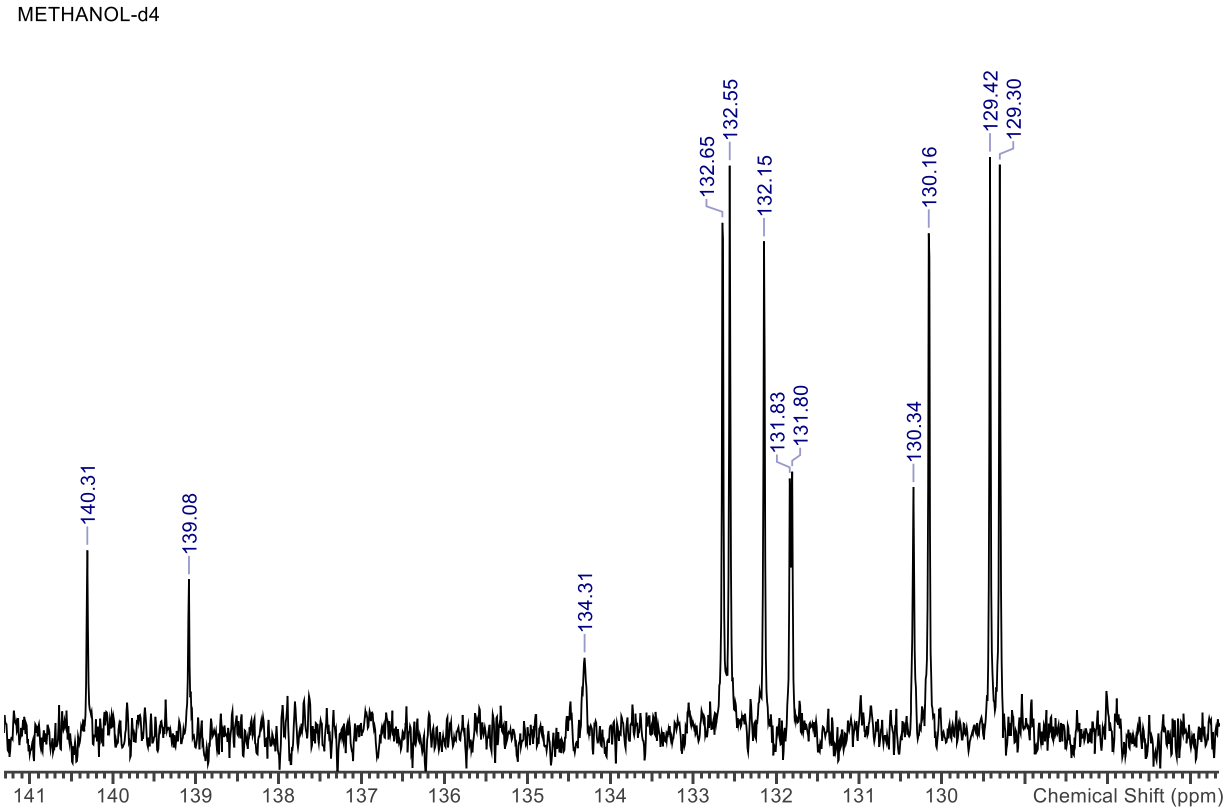

c) $^{13}C\{^{1}H\}$ NMR: [Na_2(Bn-NODP)] in in d_4-MeOH (expansion of the aromatic region)

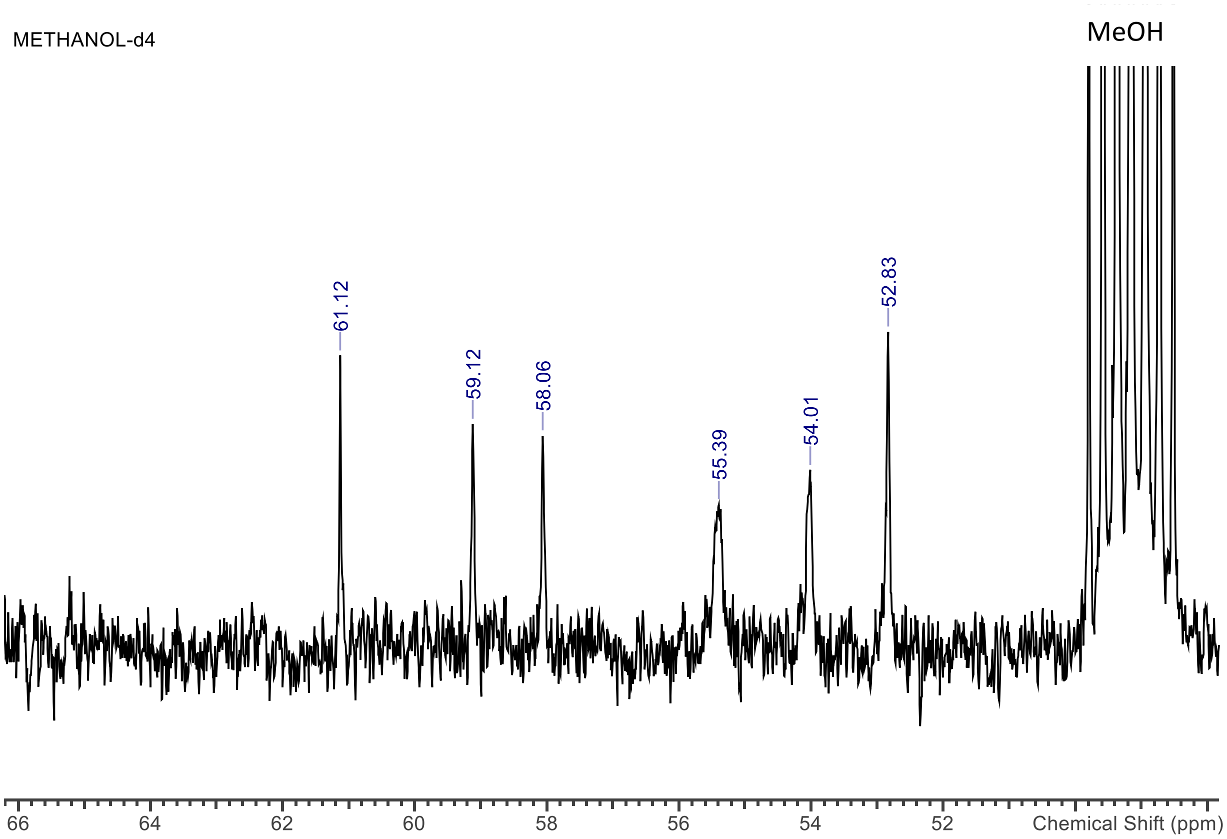

d) $^{13}C\{^{1}H\}$ NMR: Na_2(Bn-NODP) in in d_4-MeOH (expansion of the aliphatic region)

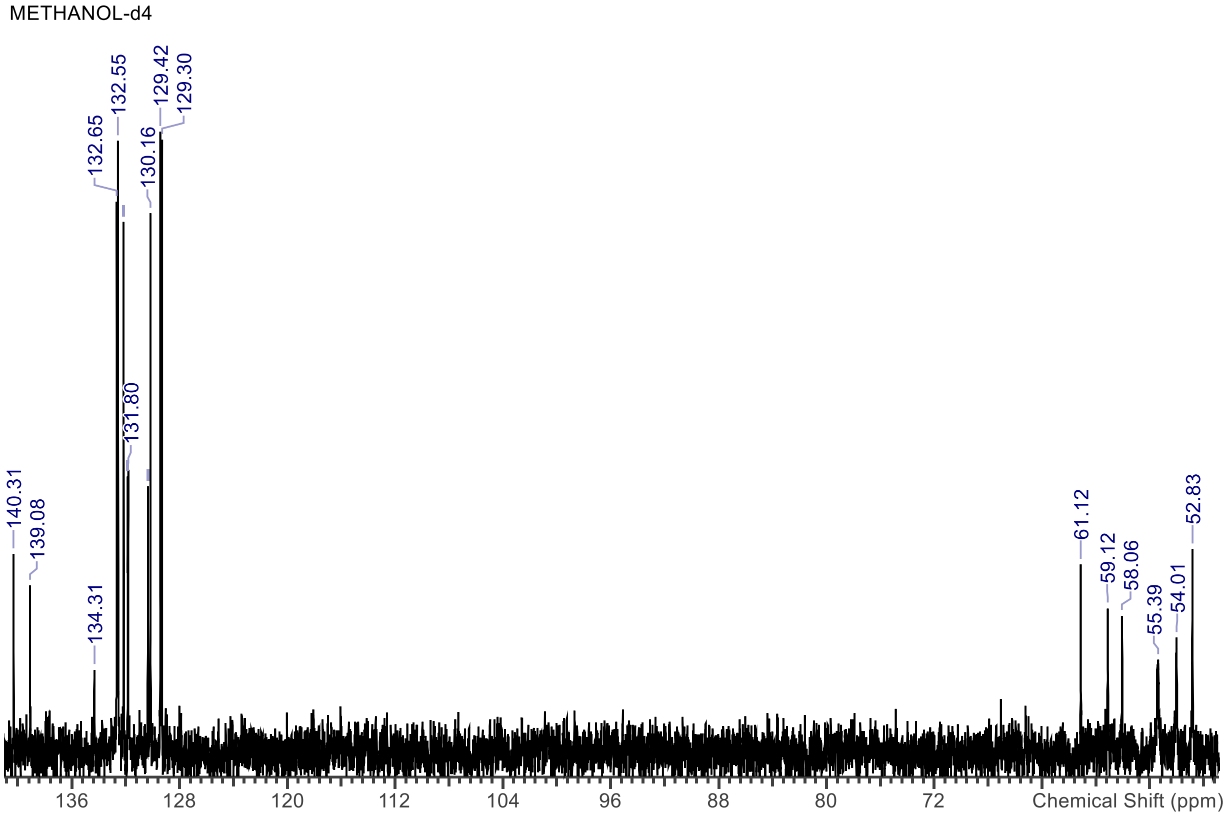

e) $^{13}C\{^{1}H\}$ NMR: Na_2(Bn-NODP) in in d_4-MeOH

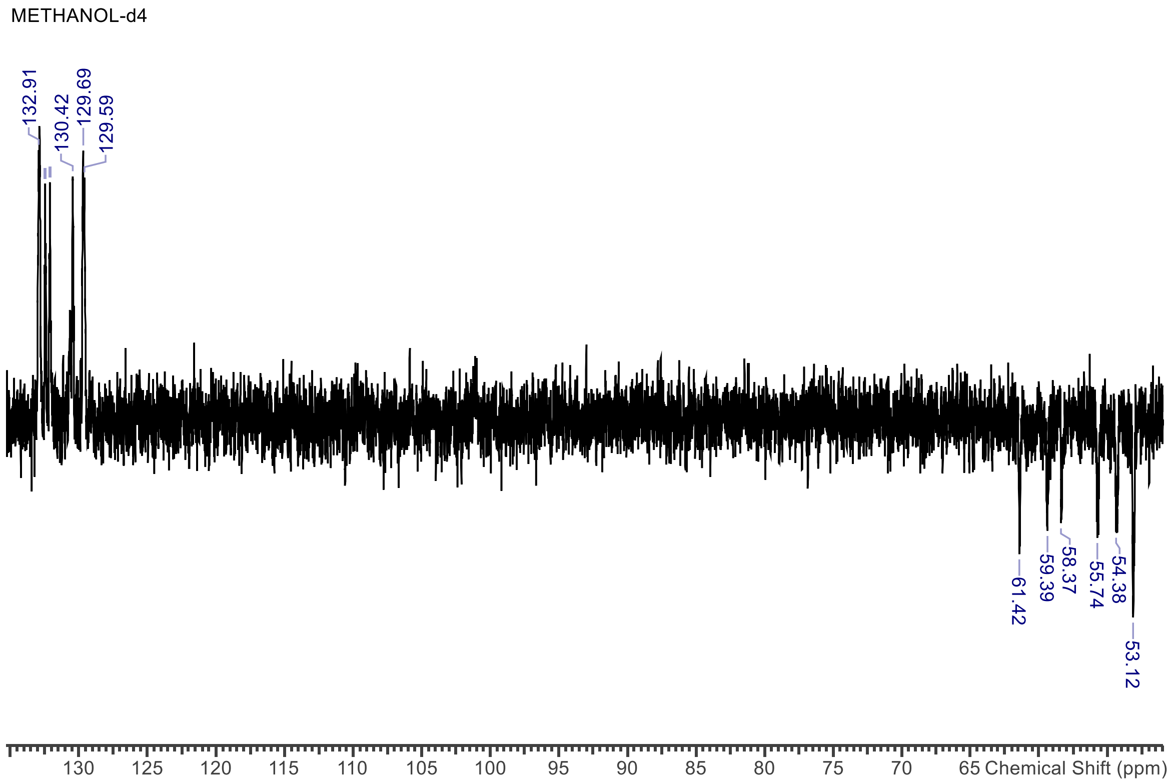

f) ^{13}C 135-dept NMR: Na_2(Bn-NODP) in d_4-MeOH

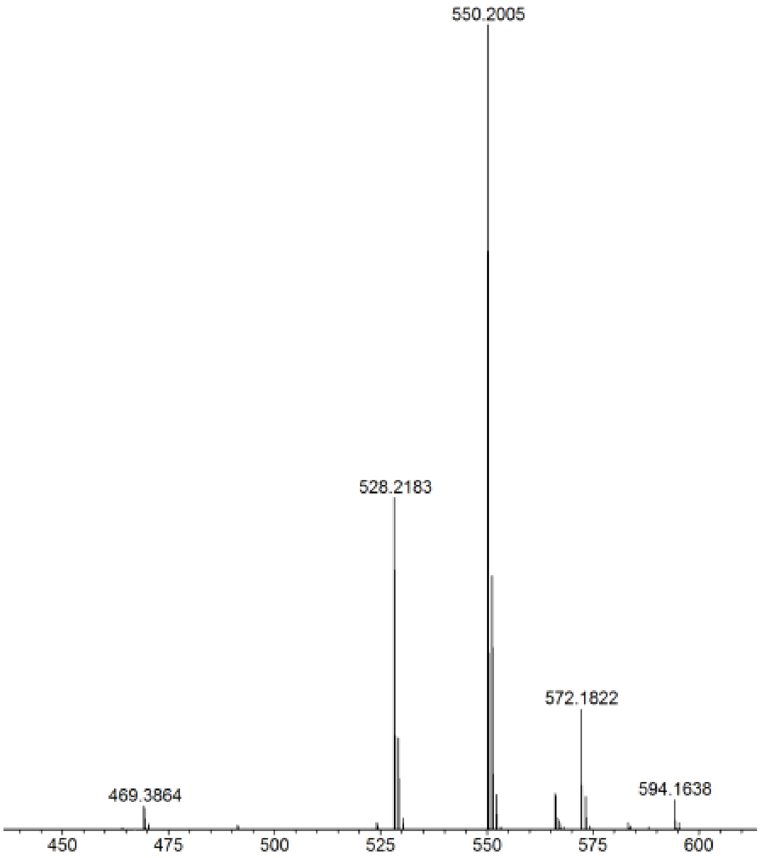

g) HRMS ESI^+ spectrum of Na_2(Bn-NODP) in MeOH

Figure S4 Spectroscopic data for [GaCl(Bn-NODP)] – Method 1

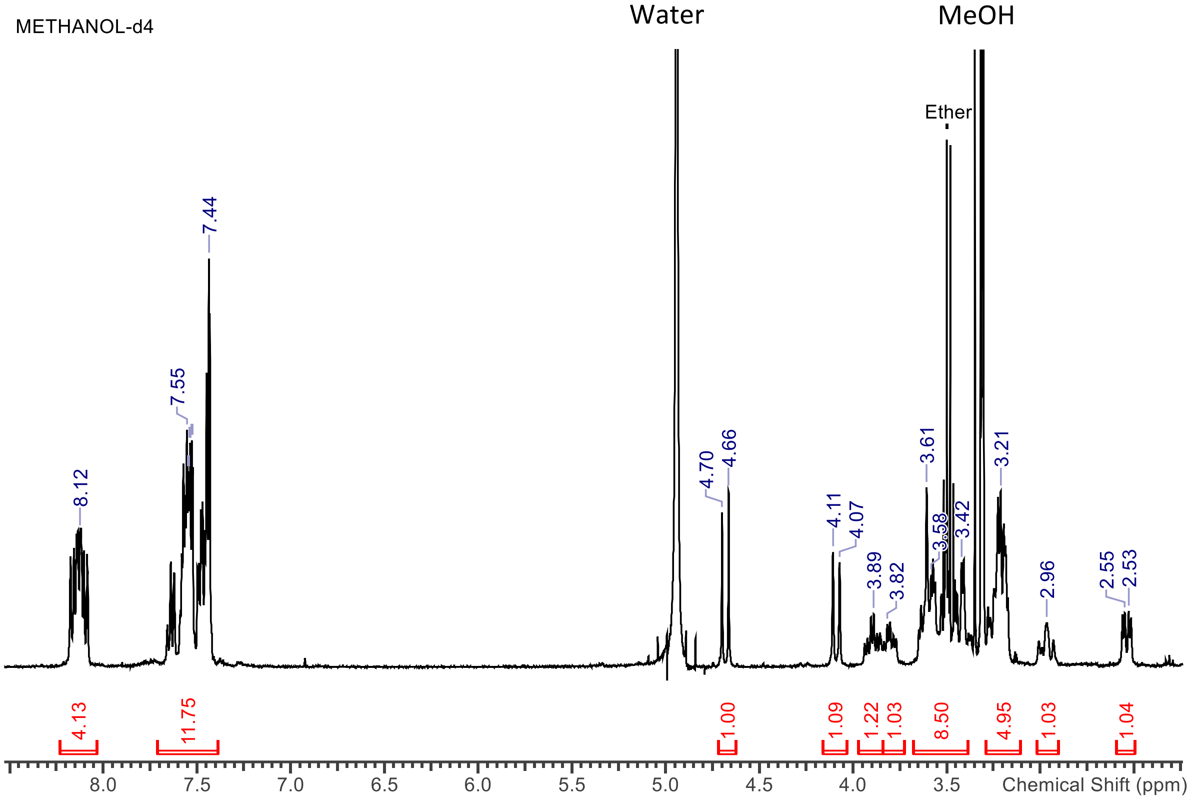

a) ^1H NMR: [GaCl(Bn-NODP)] in d_4-MeOH from $[Ga(NO_3)_3]\ 9H_2O$ + Na_2(Bn-NODP) in H_2O/HCl

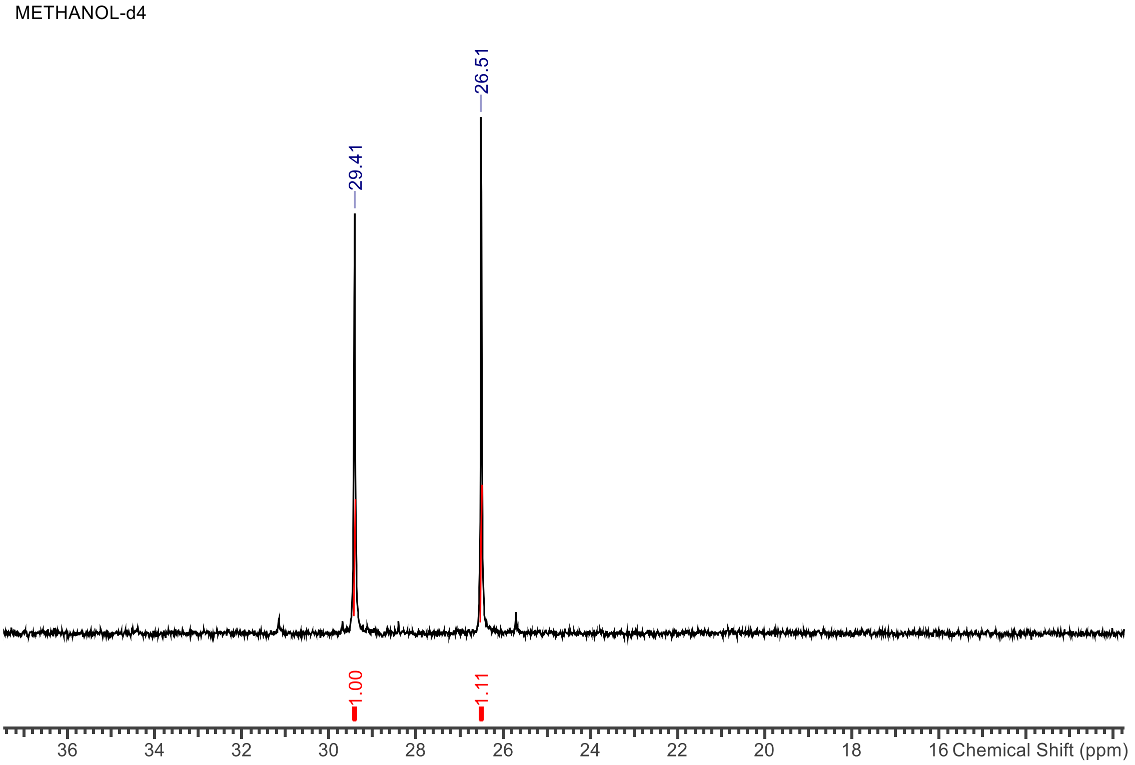

b) $^{31}P\{^1H\}$ NMR: [GaCl(Bn-NODP)] in d_4-MeOH from$[Ga(NO_3)_3]\ 9H_2O$ + Na_2(Bn-NODP) in H_2O/HCl

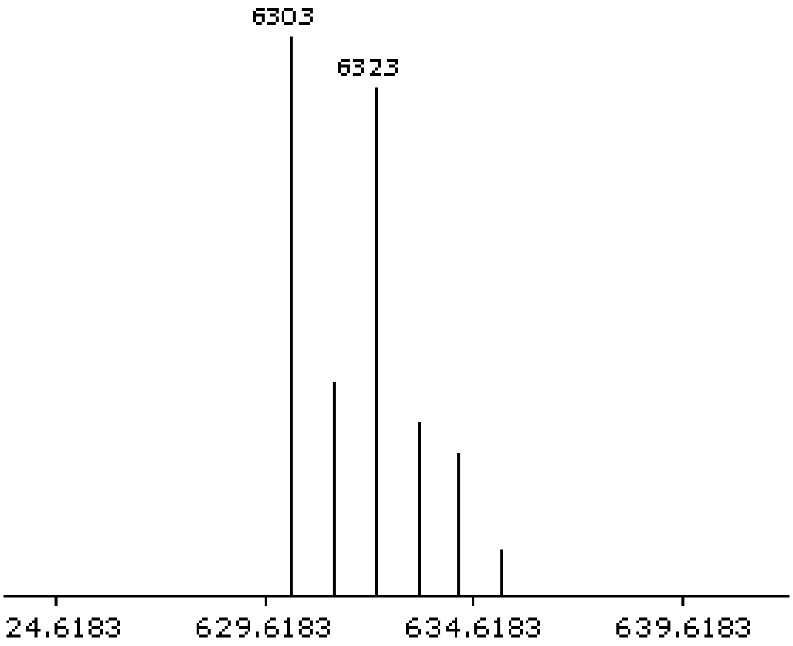

c) MS ESI$^+$ spectrum of [GaCl(Bn-NODP)] in MeOH from [Ga(NO_3)$_3$] 9H_2O + Na_2(Bn-NODP) in H_2O/HCl

Figure S4 Spectroscopic data for [GaCl(Bn-NODP)] – Method 2

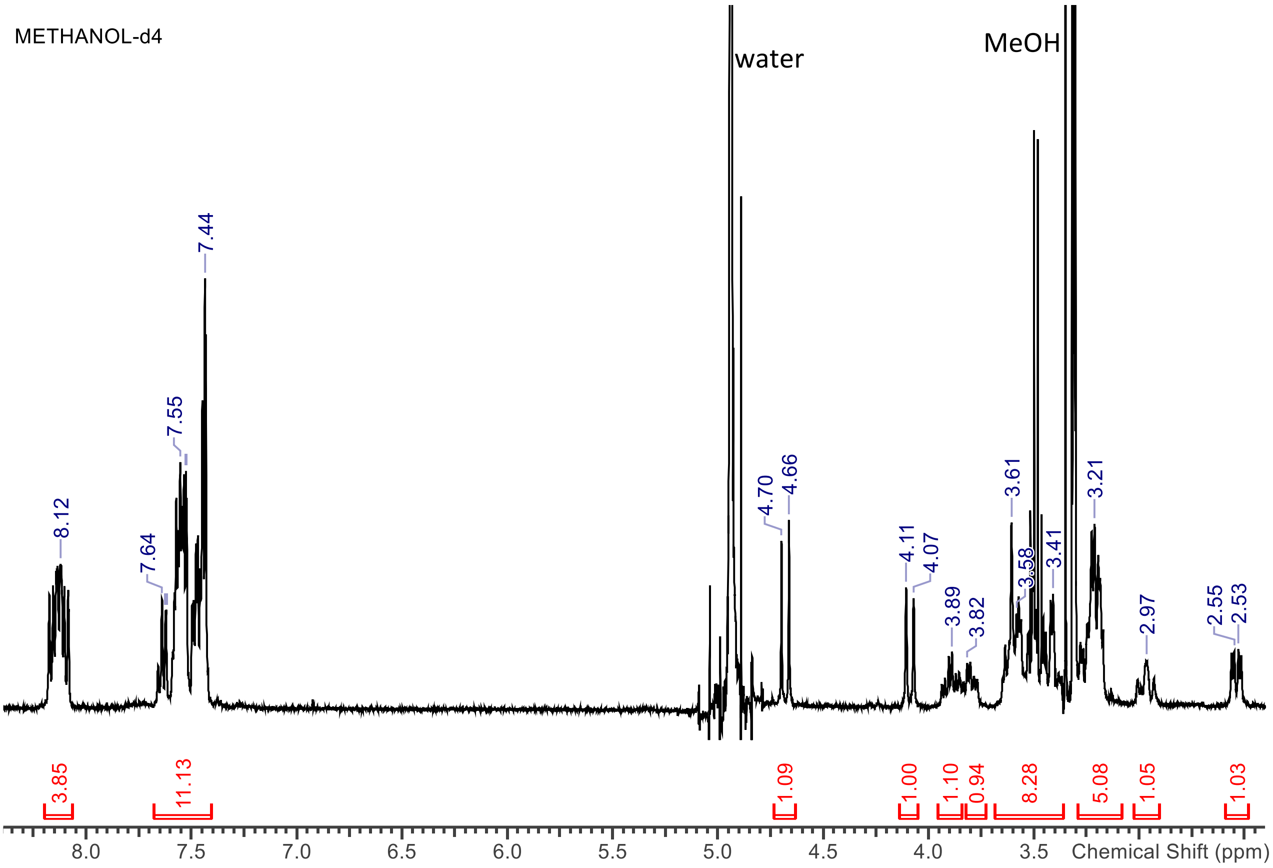

a) ^{1}H NMR: [GaCl(Bn-NODP)] in d_4-MeOH from $Ga(NO_3)_3 \cdot 9H_2O$ + H_2(Bn-NODP)·2HCl

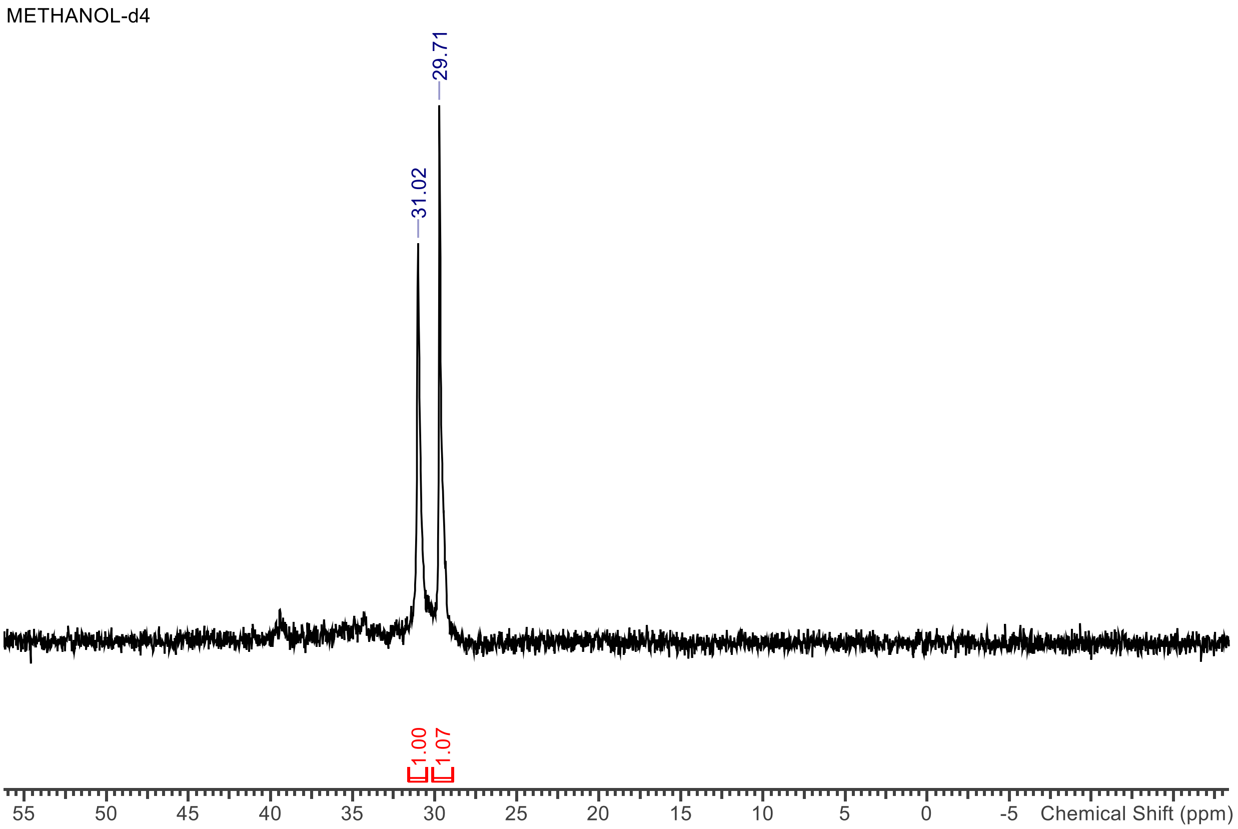

b) ^{31}P{^{1}H} NMR: [GaCl(Bn-NODP)] in d_4-MeOH from $Ga(NO_3)_3 \cdot 9H_2O$ + H_2(Bn-NODP)·2HCl

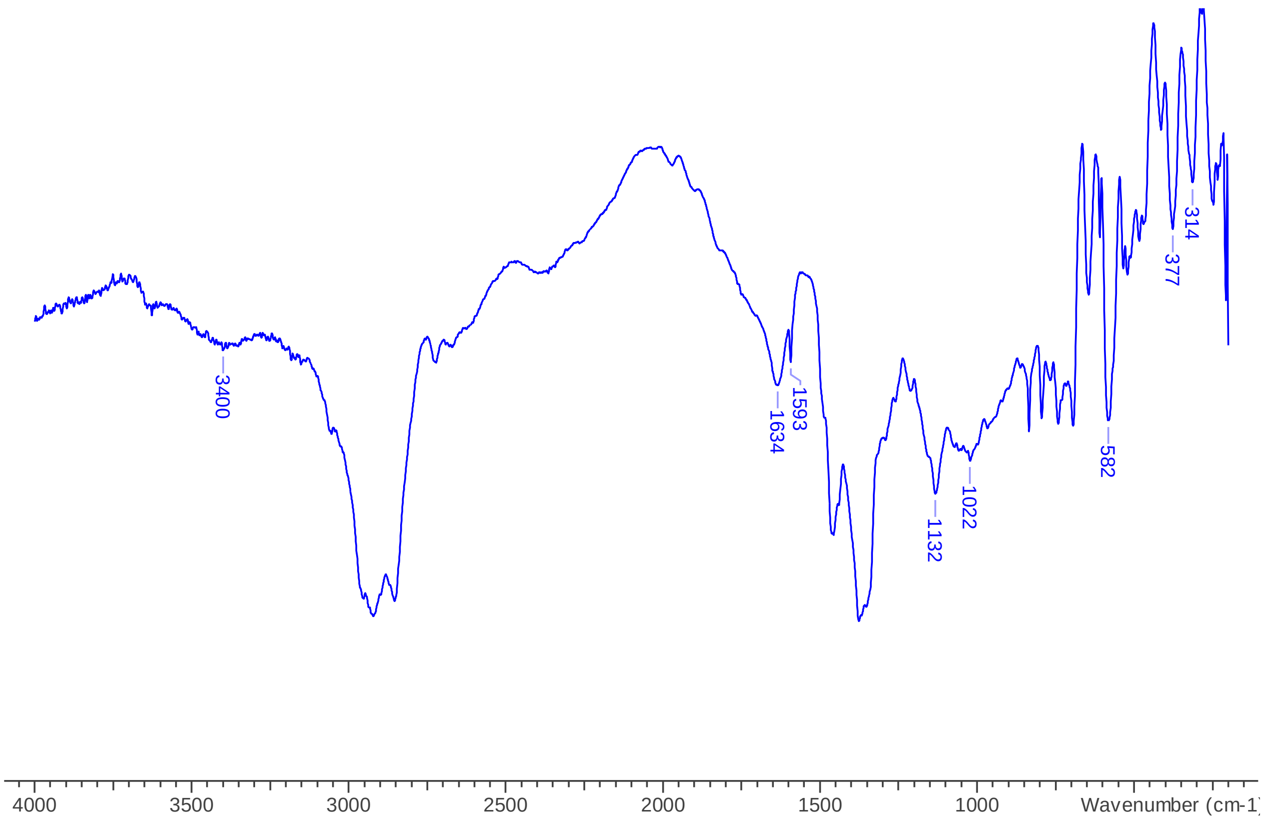

c) IR spectrum (Nujol mull) of [GaCl(Bn-NODP)] from $Ga(NO_3)_3·9H_2O$ + H_2(Bn-NODP)·2HCl

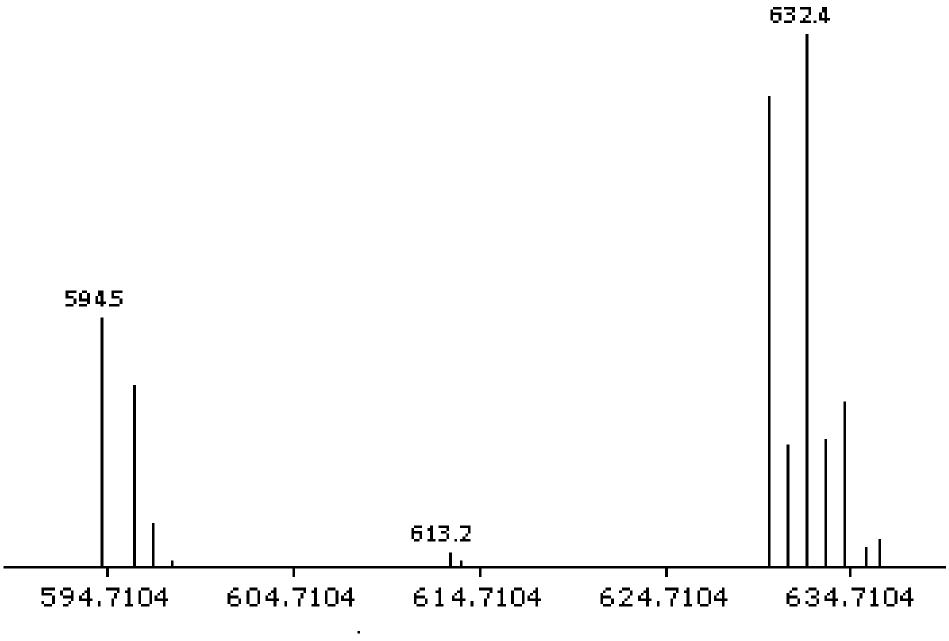

d) MS ESI$^+$ spectrum of [GaCl(Bn-NODP)] in MeOH from $Ga(NO_3)_3·9H_2O$ + H_2(Bn-NODP)·2HCl

Figure S5 Spectroscopic data for [GaCl(Bn-NODP)] – Method 3

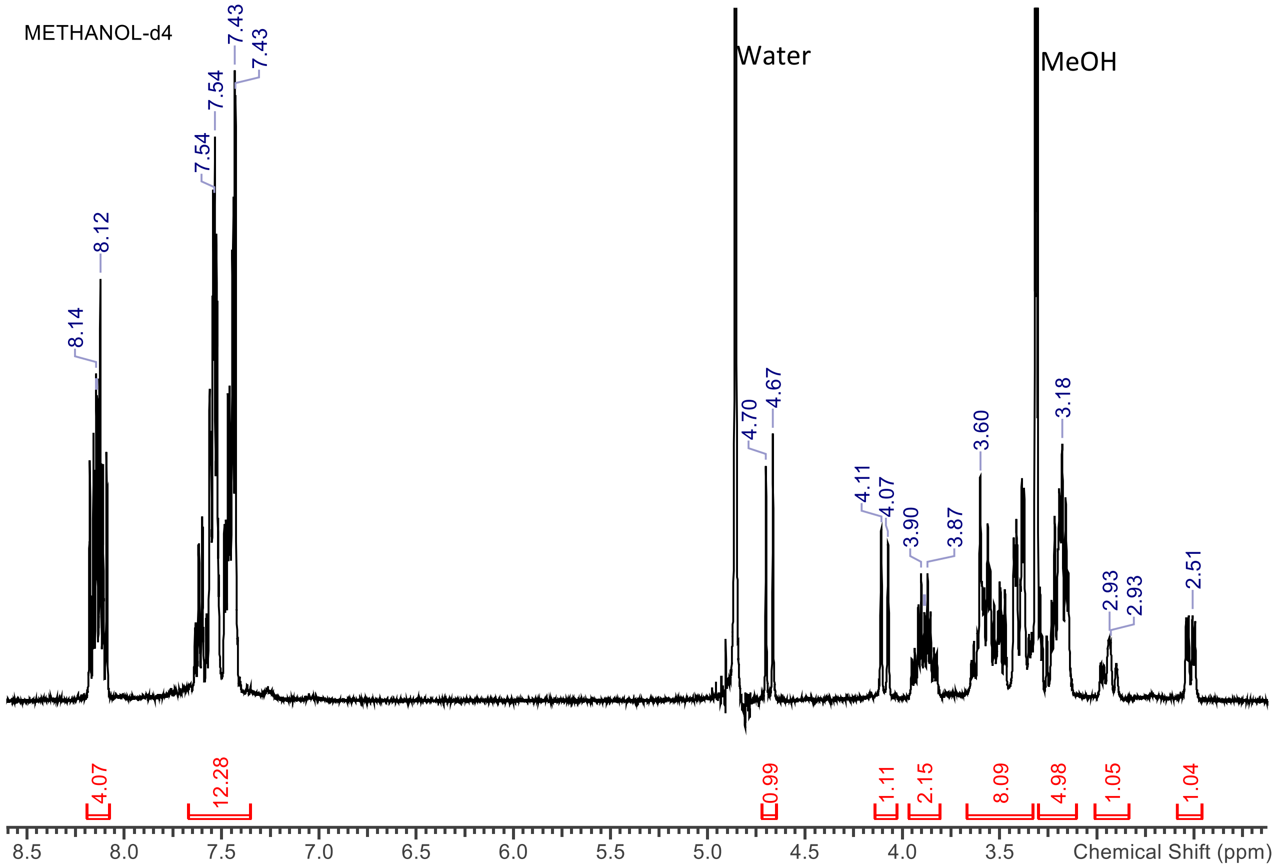

a) ^{1}H NMR: [GaCl(Bn-NODP)] in d_4-MeOH from: $GaCl_3$ + Na_2(Bn-NODP) in H_2O/HCl

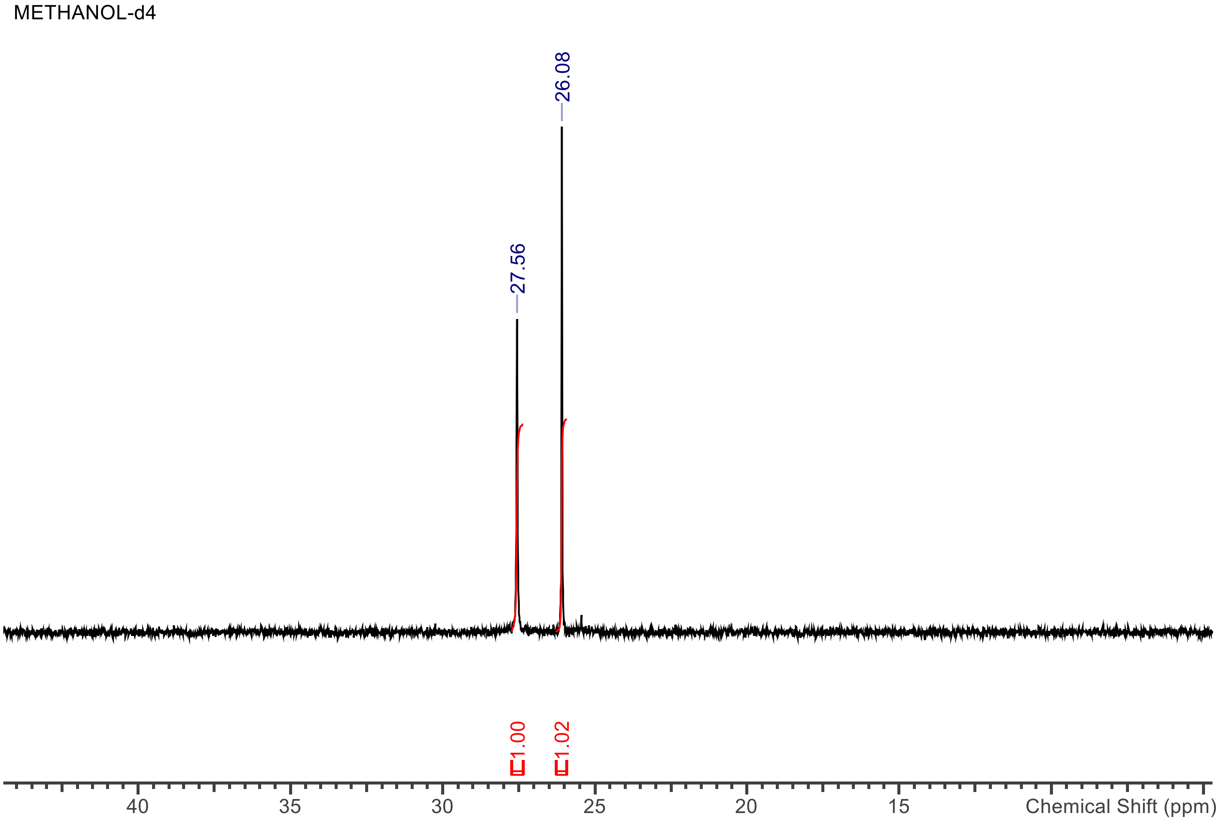

b) $^{31}P\{^{1}H\}$ NMR: [GaCl(Bn-NODP)] in d_4-MeOH from $GaCl_3$ + Na_2(Bn-NODP) in H_2O/HCl

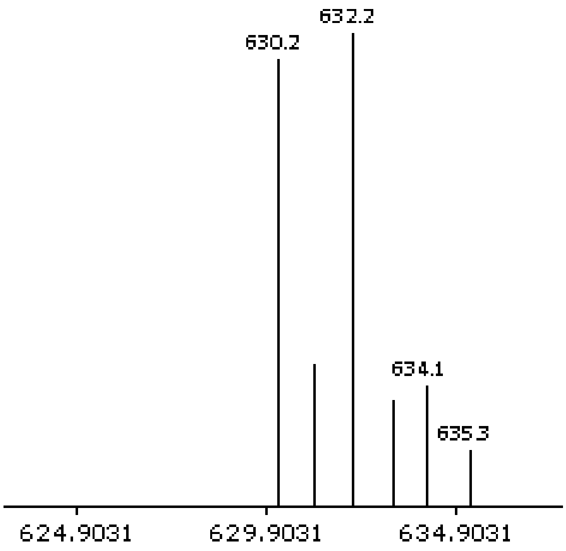

c) MS ESI$^+$ spectrum of [GaCl(Bn-NODP)] in MeOH from $GaCl_3$ + Na_2(Bn-NODP) in H_2O/HCl

Figure S6 Spectroscopic data for [GaCl(Bn-NODP)] – Method 4

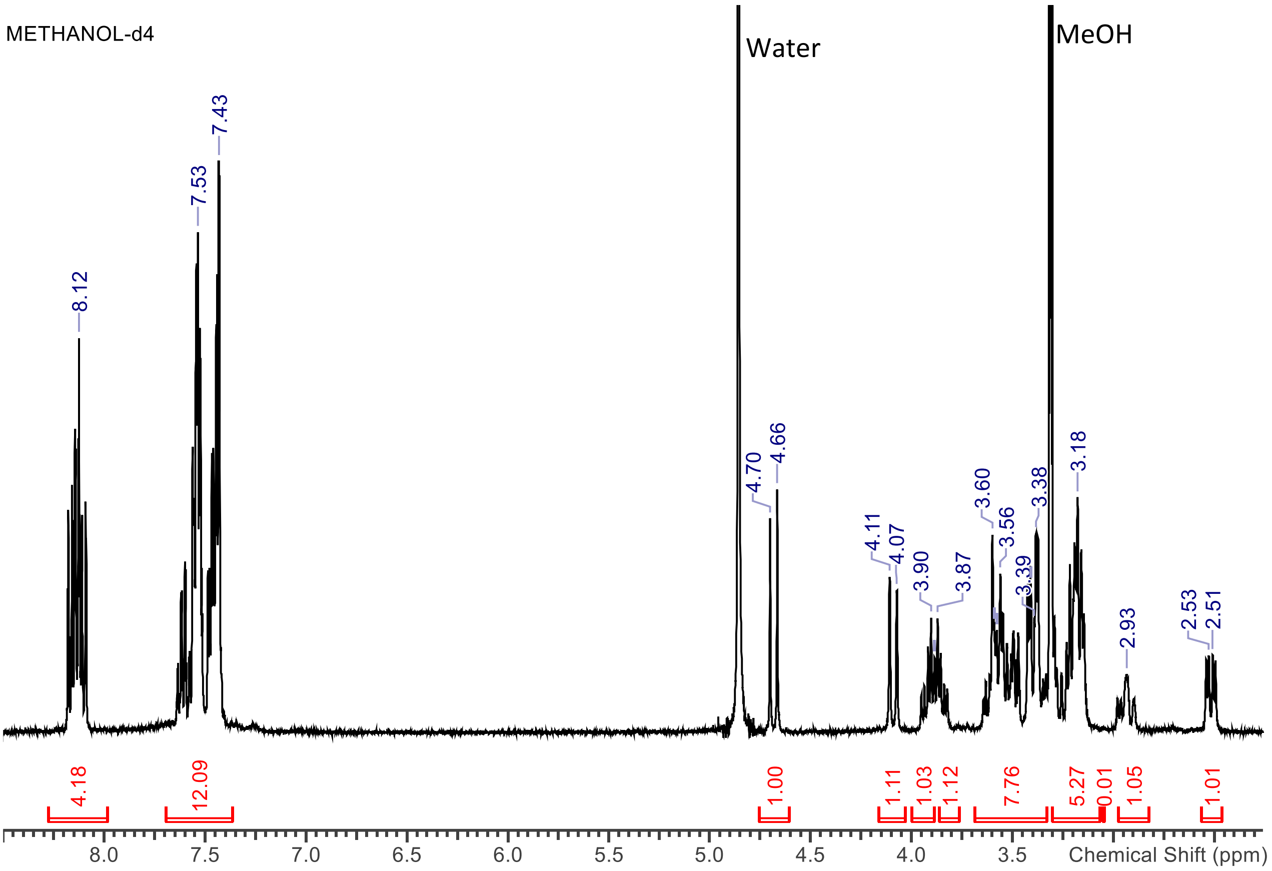

a) ^{1}H NMR: [GaCl(Bn-NODP)] in d_4-MeOH from $GaCl_3$ + H_2(Bn-NODP)·2HCl in H_2O/HCl

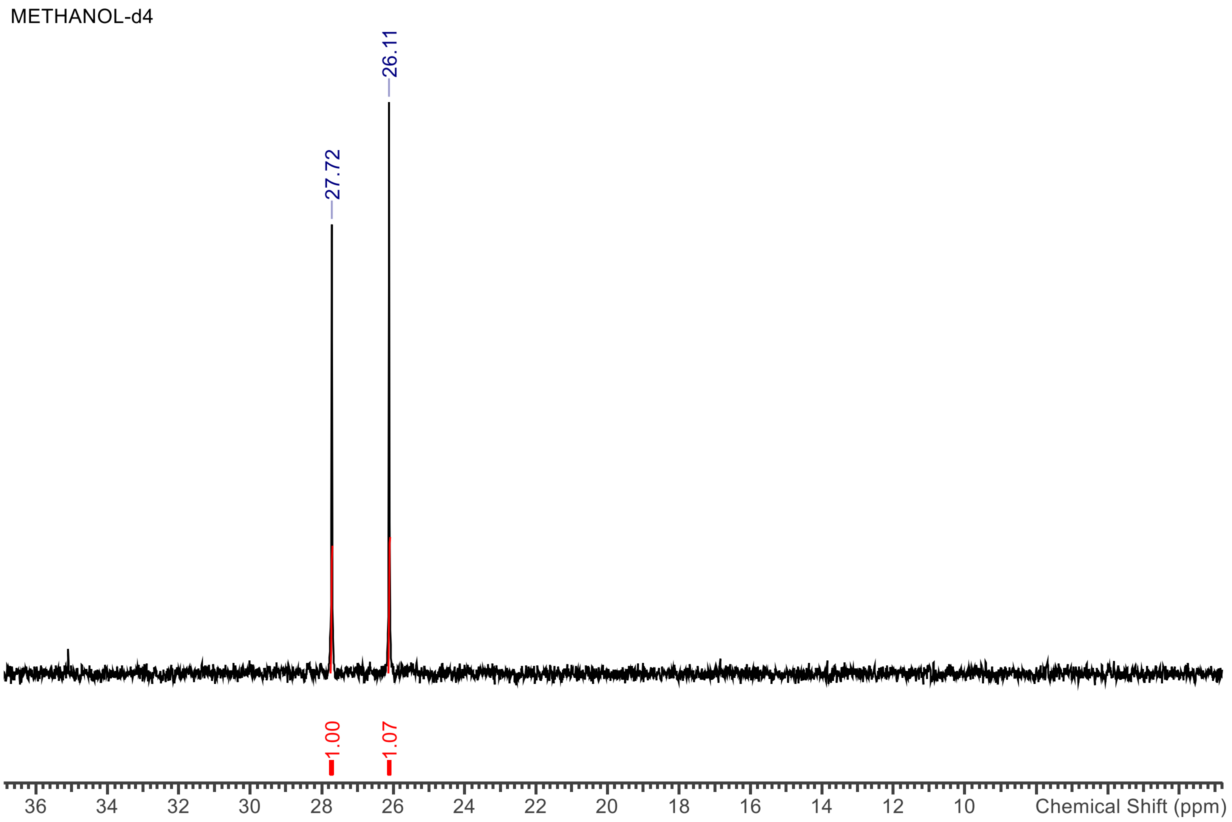

b) $^{31}P\{^{1}H\}$ NMR [GaCl(Bn-NODP)] in d_4-MeOH from $GaCl_3$ + H_2(Bn-NODP)·2HCl in H_2O/HCl

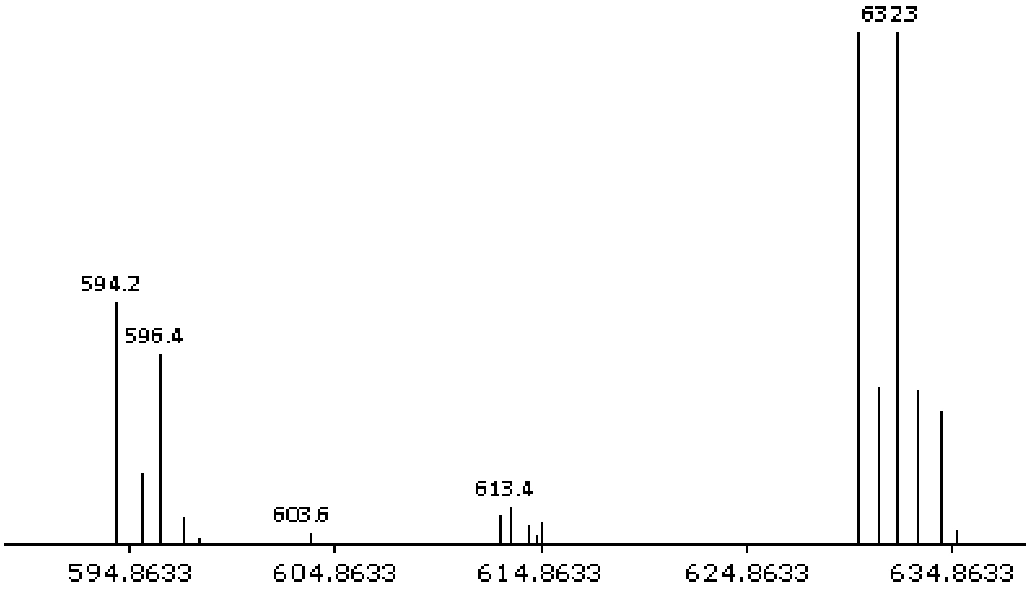

c) MS ESI$^+$ spectrum of [GaCl(Bn-NODP)] in MeOH from $GaCl_3$ + H_2(Bn-NODP)·2HCl in H_2O/HCl

Figure S6 Spectroscopic data for [GaF(Bn-NODP)]

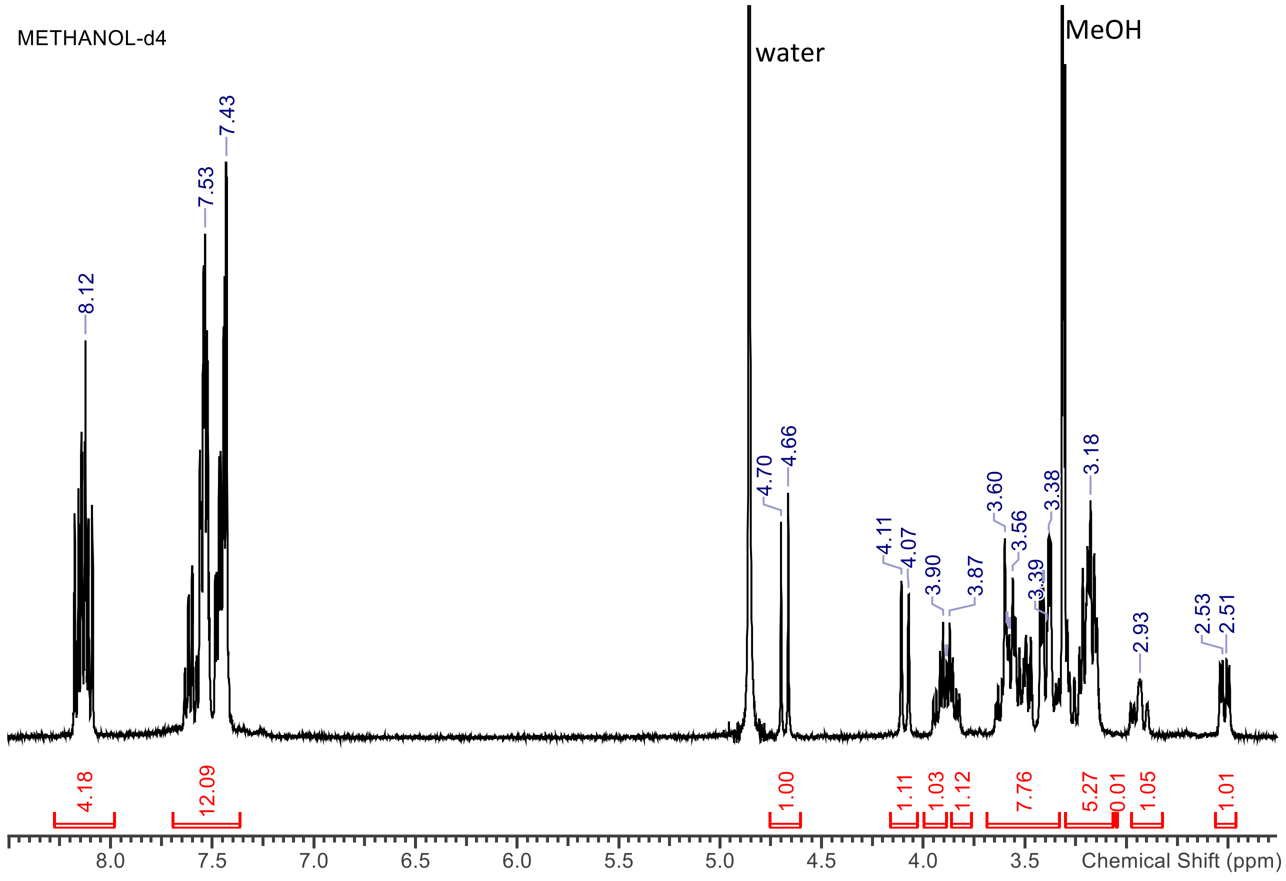

a) ^{1}H NMR: [GaF(Bn-NODP)] in d_4-MeOH

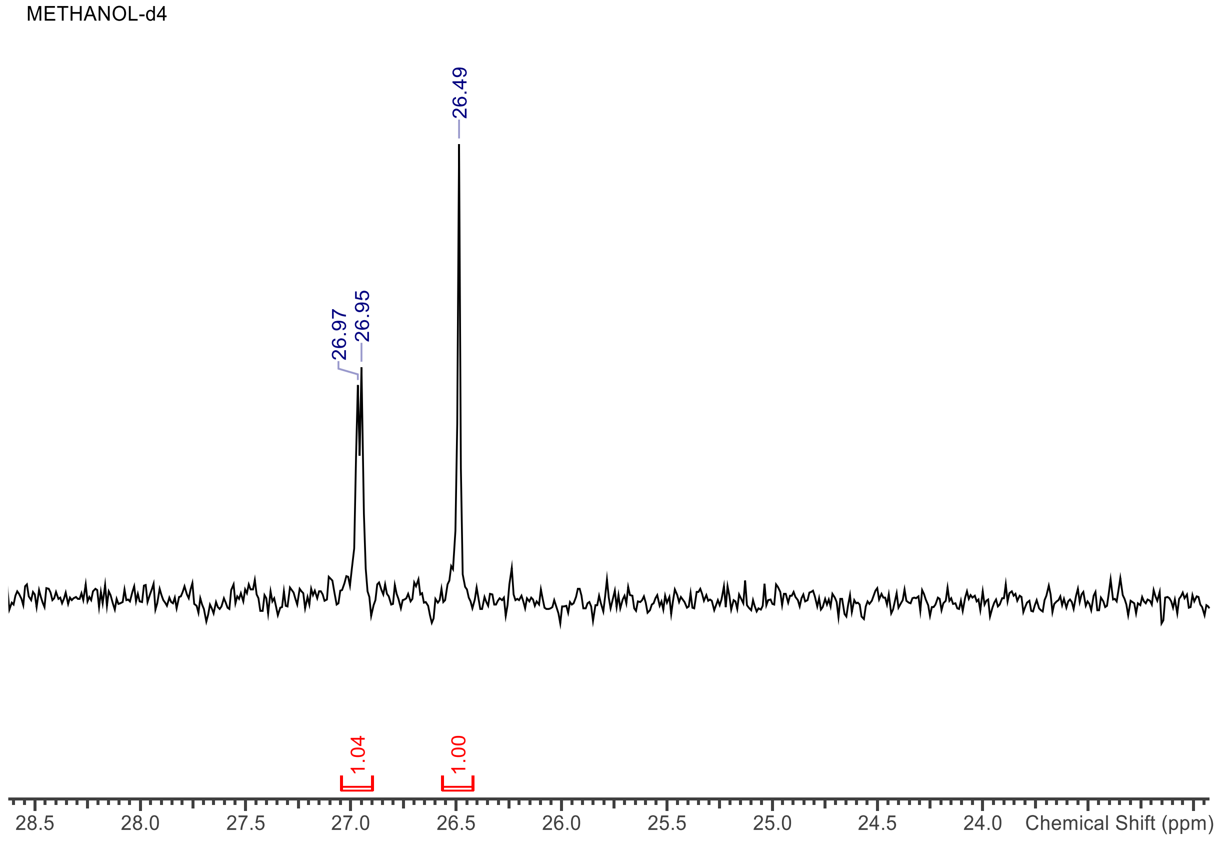

b) $^{31}P\{^{1}H\}$ NMR: [GaF(Bn-NODP)] in d_4-MeOH

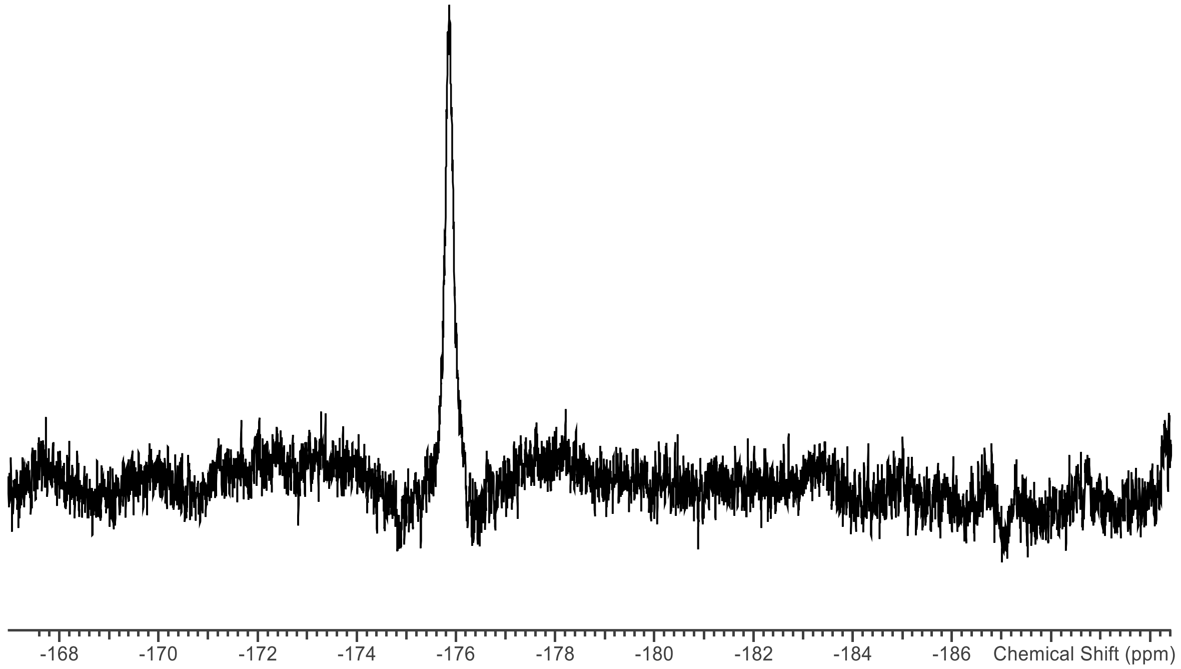

c) $^{18}F\{^{1}H\}$ NMR: [GaF(Bn-NODP)] in d_4-MeOH

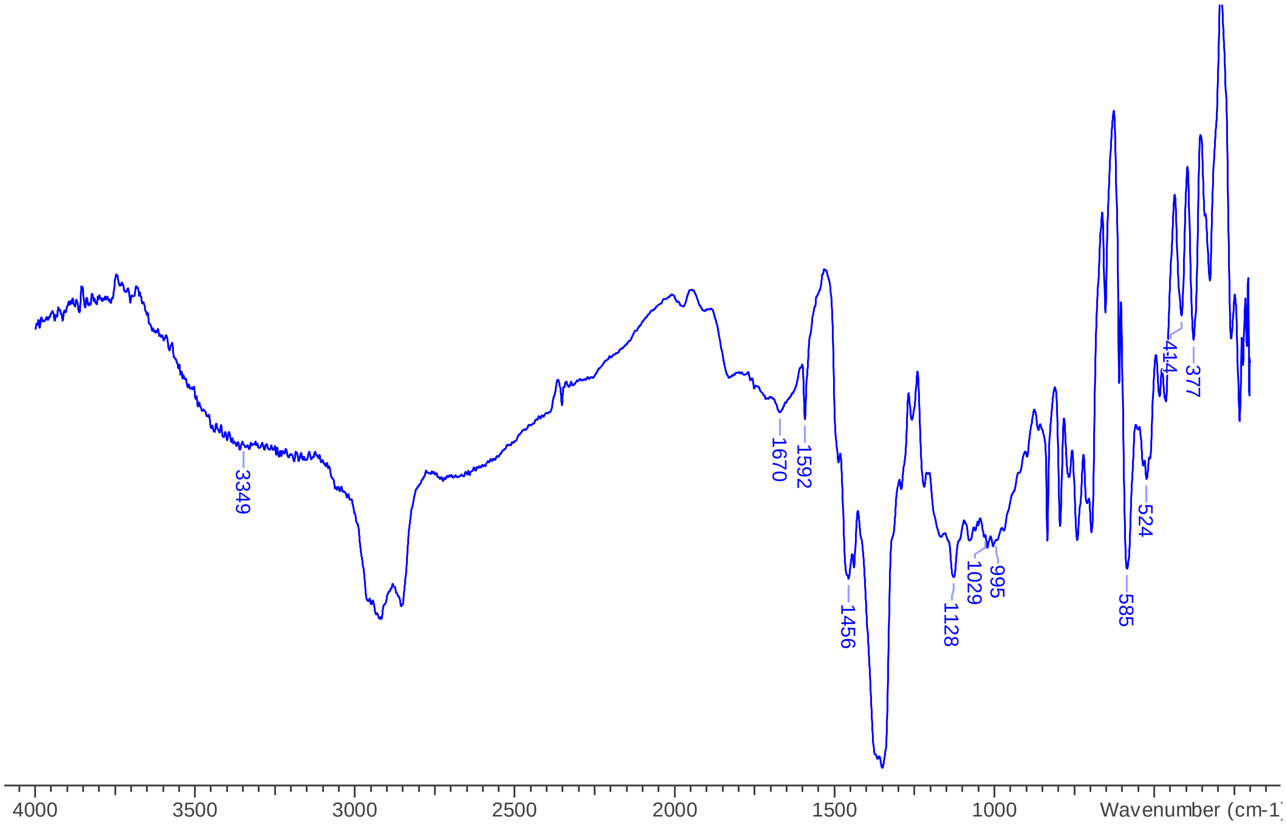

d) IR spectrum (Nujol mull) of [GaF(Bn-NODP)]

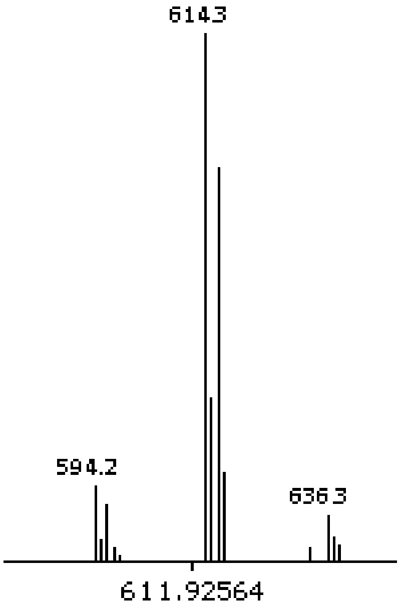

e) MS ESI⁺ spectrum of [GaF(Bn-NODP)] in MeOH

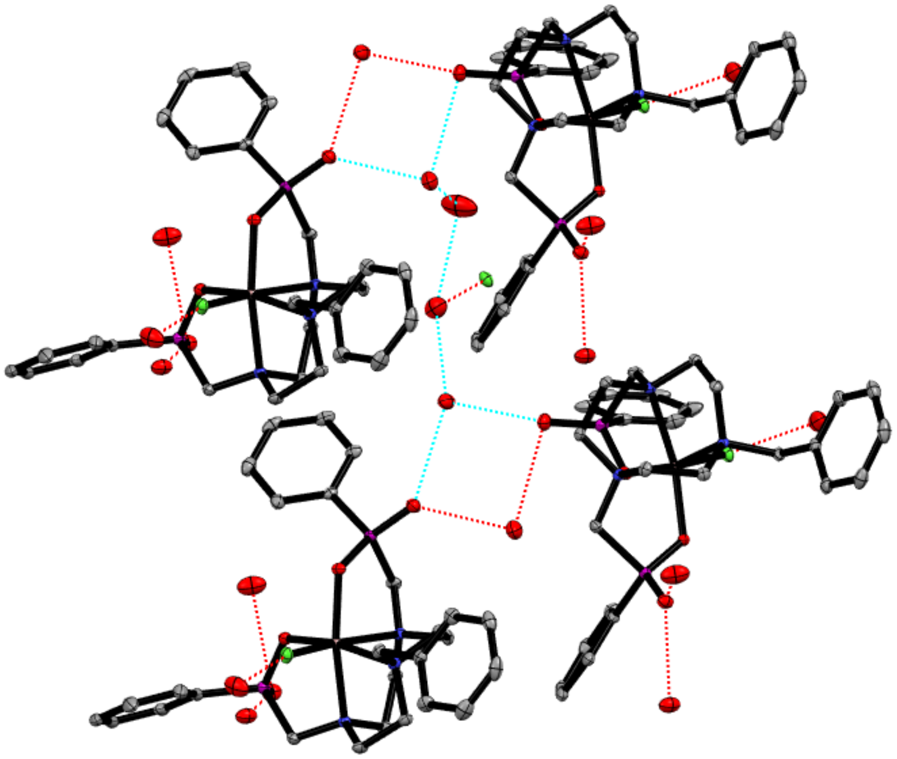

Figure S7 View of the H-bonding interactions in the structure of [GaF(Bn-NODP)]·4H_2O, showing the F···H-O (······) and O···H-O (······) hydrogen-bonding interactions.

Figure S8 Spectroscopic data for [FeCl(Bn-NODP)]

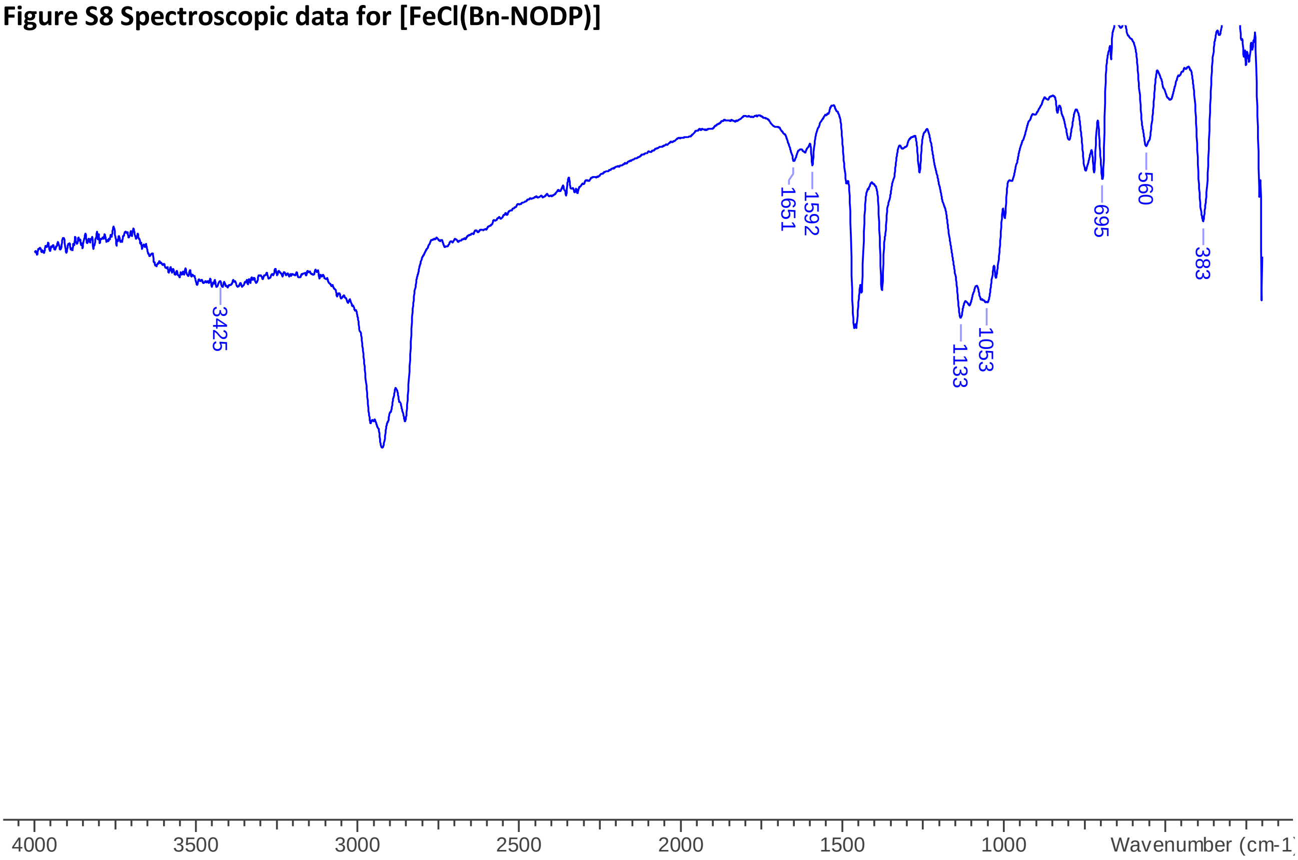

a) IR spectrum (Nujol mull) of [FeCl(Bn-NODP)]

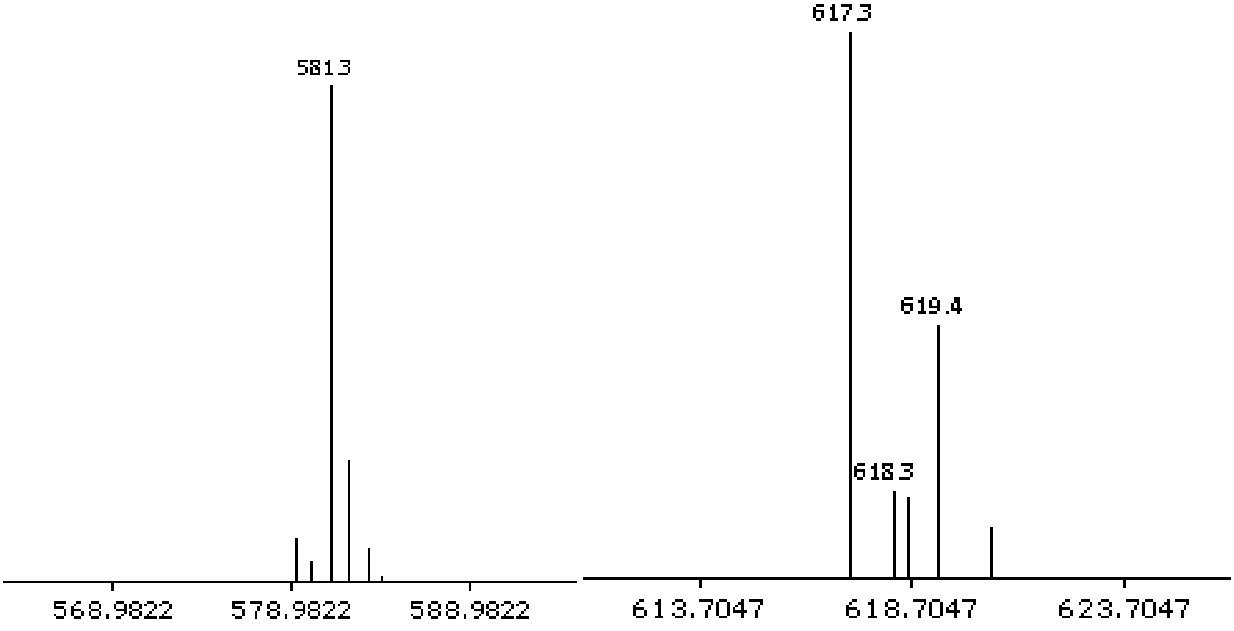

b) MS ESI^{+} spectrum of [FeCl(Bn-NODP)] in MeOH

Figure S9 Spectroscopic data for [FeF(Bn-NODP)]

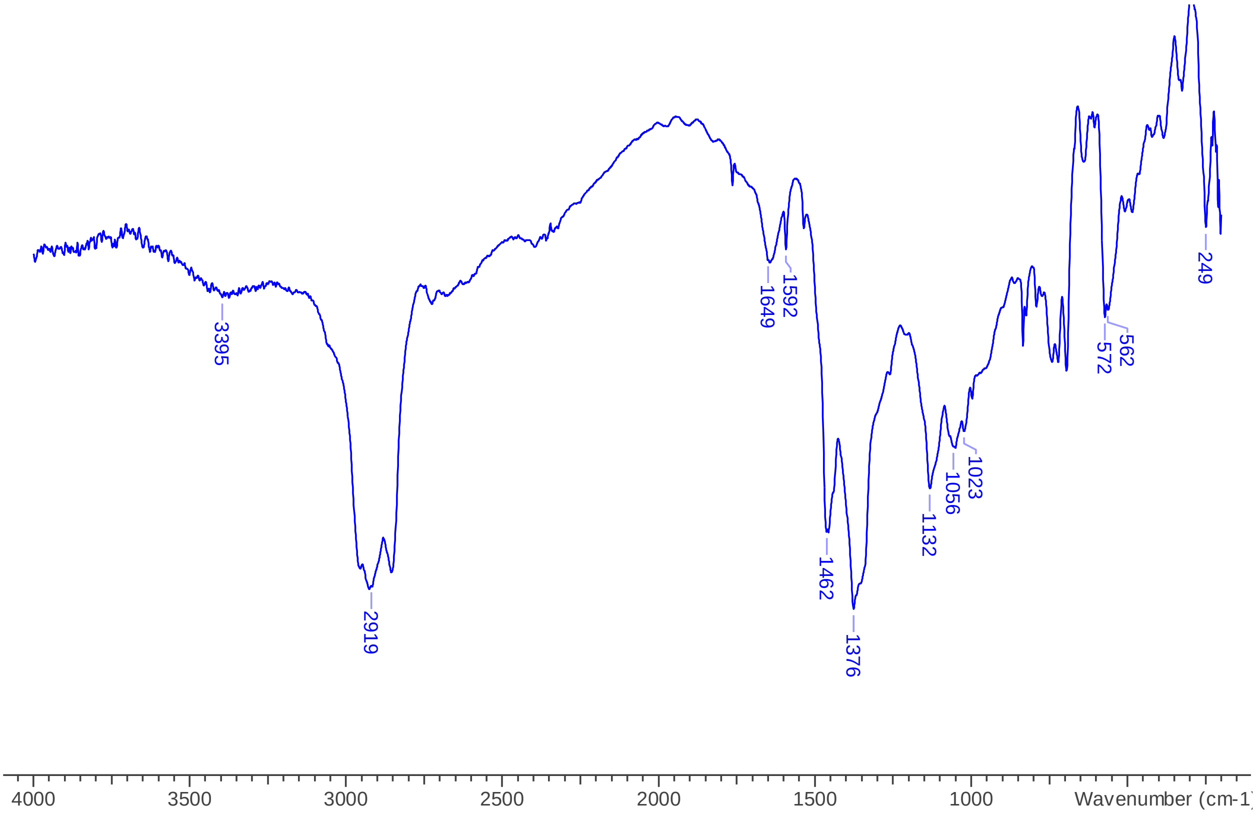

a) IR spectrum (Nujol mull) of [FeF(Bn-NODP)]

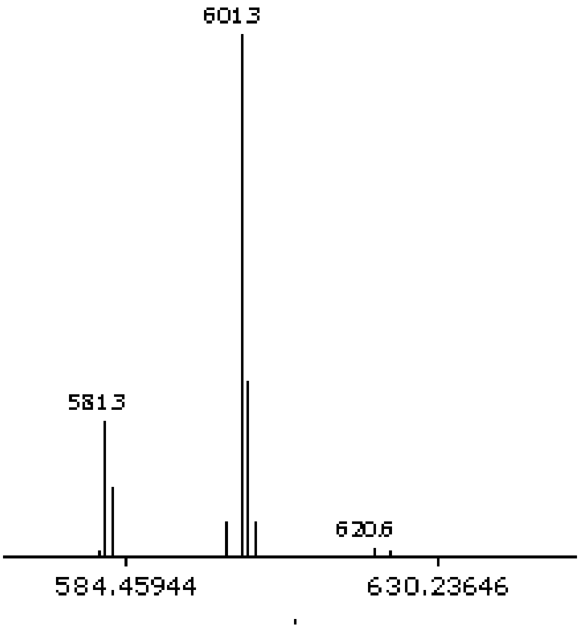

b) MS ESI$^+$ spectrum of [FeF(Bn-NODP)] in MeOH

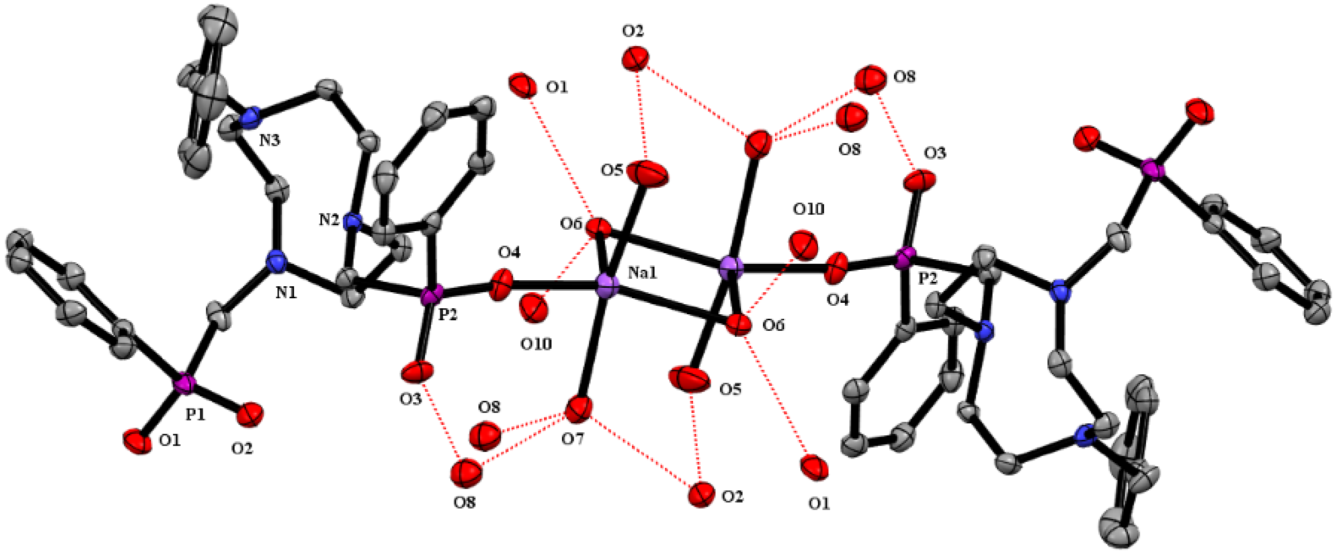

Figure S10 View of a small portion of the extended 3D structure in $[\{H(Bn\text{-}NODP)Na(H_2O)_2\}_2(\mu\text{-}OH_2)]\cdot 6H_2O$, formed through H-bonding and showing the H-bonding interactions (······) between the coordinated H_2O ligands, the phosphinate groups and the lattice water.

Table S1 X-ray crystallographic data[a]

Compound	$[H_2(Bn\text{-}NODP)]\cdot 2HCl\cdot H_2O$	$[\{H(Bn\text{-}NODP)Na(H_2O)_2\}_2(\mu\text{-}OH_2)]\cdot 6H_2O$	$[GaF(Bn\text{-}NODP)]\cdot 4H_2O$
Formula	$C_{27}H_{39}Cl_2N_3O_5P_2$	$C_{54}H_{92}N_6Na_2O_{20}P_4$	$C_{27}H_{41}FGaN_3O_8P_2$
M	618.45	1315.19	686.29
Crystal system	Monoclinic	Triclinic	Orthorhombic
Space group (no.)	$P2_1/n$ (14)	$P\bar{1}$ (2)	$Pna2_1$ (33)
a /Å	17.6440(5)	9.1875(2)	19.7067(3)
b /Å	6.9152(2)	9.8137(2)	9.51900(10)
c /Å	25.6780(7)	18.3971(3)	16.2977(2)
α /°	90	85.999(2)	90
β /°	107.947(3)	87.795(2)	90
γ /°	90	75.081(2)	90
U /$Å^3$	2980.57(15)	1598.56(6)	3057.25(7)
Z	4	1	4
μ(Mo-K_α) /mm^{-1}	0.367	0.208	1.063
F(000)	1304.0	700	1432.0
Total number reflns	36118	33645	78125
R_{int}	0.042	0.031	0.038
Unique reflns	7605	8259	7871
No. of params, restraints	357, 0	428, 0	391, 1
GOF	1.130	0.973	1.033
R_1, wR_2 [$I > 2\sigma(I)$][b]	0.058, 0.144	0.046, 0.121	0.021, 0.051
R_1, wR_2 (all data)[b]	0.072, 0.151	0.060, 0.130	0.024, 0.052

[a] common data: T = 100 K; wavelength (Mo-K_α) = 0.71073 Å; θ(max) = 27.5°; [b] $R_1 = \Sigma||F_o| - |F_c||/\Sigma|F_o|$; $wR_2 = [\Sigma w(F_o^2 - F_c^2)^2 / \Sigma wF_o^4]^{1/2}$.

DFT Calculations

Table S2 Total and relative energy (ΔE and ΔG^{ϕ}_{298K}) of [GaF(Bn-NODP)] isomers at the B3LYP-D3 and BP86-D3 levels

(a) B3LYP-D3

Isomer	Total SCF Energy, E / a.u	Relative Energy ΔE a.u.; in brackets kcal mol^{-1}	Standard Free Energy $G^{\phi}_{298K,}$ / a.u.	Relative Free Energy $\Delta G^{\phi}_{298K,}$ / kcal mol^{-1}	Sum of HB Energies ΣHB_i / kcal mol^{-1} (no. of HBs)
***Isomer 1**/SS*	-4222.<br>207078	0.02533<br>(15.89)	-4221.<br>695559	15.42293	20.25<br>(11)
***Isomer 2**/SS*	-4222.<br>212544	0.01986<br>(12.46)	-4221.<br>700289	12.45481	29.30<br>(11)
***Isomer 2**/RR*	-4222.<br>214339	0.01807<br>(11.34)	-4221.<br>214339	11.3391	26.52<br>(11)
***Isomer 1**/SR*	-4222.<br>219575	0.01283<br>(8.05)	-4221.<br>707356	8.0202	26.10<br>(12)
***Isomer 1**/RS*	-4222.<br>219990	0.01242<br>(7.79)	-4221.<br>708366	7.386416	22.79<br>(10)
***Isomer 2**/RS*	-4222.<br>223941	0.00848<br>(5.31)	-4221.<br>711775	5.247235	29.90<br>(10)
***Isomer 1**/RR*	-4222.<br>232407	0.00	-4221.<br>720137	0.00	27.34<br>(11)

(b) BP86-D3

Isomer	Total SCF Energy, E / a.u	Relative Energy ΔE a.u.; in brackets kcal mol^{-1}	Standard Free Energy $G^{\phi}_{298K,}$ / a.u.	Relative Free Energy $\Delta G^{\phi}_{298K;}$ / kcal mol^{-1}	Sum of HB Energies ΣHB_i / kcal mol^{-1} (no of HBs)
***Isomer 1**/SS*	-4222.<br>438703	0.021138<br>(13.42)	-4221.<br>946424	13.17331	22.20<br>(12)
***Isomer 2**/SS*	-4222.<br>442832	0.01726<br>(10.83)	-4221.<br>951710	9.856293	31.18<br>(12)
***Isomer 2**/RR*	-4222.<br>443971	0.016117<br>(10.11)	-4221.<br>950080	10.87913	20.63<br>(11)
***Isomer 1**/SR*	-4222.<br>449286	0.01080<br>(6.78)	-4221.<br>956568	6.807852	27.24<br>(12)
***Isomer 1**/RS*	-4222.<br>449669	0.01042<br>(6.54)	-4221.<br>95753	6.204187	26.42<br>(12)
***Isomer 2**/RS*	-4222.<br>452089	0.00799<br>(5.02)	-4221.<br>959413	5.022587	30.73<br>(10)
***Isomer 1**/RR*	-4222.<br>460088	0.00	-4221.<br>967417	0.00	30.08<br>(12)

Table S3 Nuclear-Nuclear Repulsion Energies, E_{nn} of GaF(Bn-NODP) isomers at the B3LYP-D3 and BP86-D3 levels

Isomer	B3LYP-D3 E_{nn}	B3LYP-D3 Relative E_{nn} a.u.; in brackets kcal.mol^{-1}	BP86-D3 E_{nn}	BP86-D3 Relative E_{nn} a.u.; in brackets kcal.mol
***Isomer 1**/SS*	6224.5645561432	47.97263 (30103.29)	6218.7186282315	58.294718 (36580.50)
***Isomer 2**/SS*	6215.3818797427	38.78995 (24341.07)	6204.1512889696	43.727379 (27439.35)
***Isomer 2**/RR*	6195.8116547774	19.21973 (12060.56)	6184.8007243653	24.376814 (15296.69)
***Isomer 1**/SR*	6199.3118010873	22.71987 (14256.94)	6188.4629881521	28.039078 (17594.79)
***Isomer 1**/RS*	6203.8383774725	27.24645 (17097.41)	6192.2706516973	31.846741 (19984.14)
***Isomer 2**/RS*	6177.2935938257	0.70167 (440.30)	6159.2249375389	-1.1989727 (-752.37)
***Isomer 1**/RR*	6176.5919274651	0	6160.4239102618	0

Table S4 Comparison of the computed bond lengths and angles for the GaCl(Bn-NODP) Isomer 1/RR, at the B3LYP-D3 and BP86-D3 levels

Bond Lengths

Optimised GaCl(Bn-NODP)		
Gaussian numbers (GaCl)	B3LYP-D3 / Å	BP86-D3 / Å
Ga1-Cl2	2.28498	2.29129
Ga1-O6	1.89109	1.90630
Ga1-O3	1.94008	1.95434
Ga1-N5	2.20721	2.20684
Ga1-N4	2.24307	2.23772
Ga1-N12	2.23141	2.22557
P68-O3	1.57140	1.58737
P68-O71	1.49831	1.51310
P69-O6	1.57483	1.59119
P69-O70	1.49697	1.51161

Bond Angles

Optimised GaCl(Bn-NODP)		
Gaussian numbers (GaCl)	B3LYP-D3 / °	BP86-D3 / °
O3-Ga1-N5	84.37186	85.20574
O3-Ga1-N4	88.23407	87.94866
O6-Ga1-N4	84.87851	85.91754
O6-Ga1-N12	91.81696	91.40612
O3-P68-C43	100.46348	100.88171
O6-P69-C20	100.90984	101.41835
Cl2-Ga1-N5	94.61898	94.11281
Cl2-Ga1-N4	171.65632	171.70083
Cl2-Ga1-N12	93.76840	93.84740
Cl2-Ga1-O3	96.82084	96.84645

Cl2-Ga1-O6	100.67948	99.87921

Table S5 Computed Mulliken charges on the Ga atom and the six atoms bonded to Ga (F, N, N, $N_{(trans\ Bn)}$, O, O) in the optimized structures of the seven isomers shown in Table 2 [GaF(Bn-NODP)] at the B3LYP-D3 and BP86-D3 levels, as well as the Mulliken charges on all atoms for structures 1/RR and 2/RS.

Mulliken totals ***1***/*RR*:

**Atom**	**Atom no**	**B3LYP-D3**	**BP86-D3**
Ga	1	1.491165	1.296008
F	4	-0.527094	-0.4765
N	10	-0.541892	-0.47934
N	11	-0.548677	-0.48883
$N_{(trans\ Bn)}$	9	-0.552810	-0.48984
O	5	-0.780415	-0.70253
O	6	-0.802515	-0.7223

Mulliken totals ***2/***_RS_:

**Atom**	**Atom no**	**B3LYP-D3**	**BP86-D3**
Ga	1	1.515325	1.322455
F	3	-0.543349	-0.486951
N	5	-0.549937	-0.489496
N	6	-0.550484	-0.492558
$N_{(trans\ Bn)}$	14	-0.548670	-0.485273
O	8	-0.778700	-0.700806
O	2	-0.775676	-0.698419

Mulliken totals ***1***/*RS*:

**Atom**	**Atom no**	**B3LYP-D3**	**BP86-D3**
Ga	1	1.517375	1.320882
F	2	-0.514378	-0.465336
N	4	-0.536252	-0.474594
N	5	-0.536427	-0.475875
$N_{(trans\ Bn)}$	12	-0.548574	-0.486776
O	3	-0.809015	-0.728458
O	6	-0.779896	-0.702473

Mulliken totals ***1***/*SR*:

**Atom**	**Atom no**	**B3LYP-D3**	**BP86-D3**
Ga	1	1.516848	1.322100
F	2	-0.525202	-0.475128
N	4	-0.529055	-0.465961
N	5	-0.545712	-0.486627
$N_{(trans\ Bn)}$	12	-0.544178	-0.482088
O	3	-0.775075	-0.694449
O	6	-0.787013	-0.708932

Mulliken totals ***2***/*RR*:

**Atom**	**Atom no**	**B3LYP-D3**	**BP86-D3**
Ga	1	1.536884	1.345038
F	3	-0.528925	-0.474972
N	4	-0.546954	-0.487064

N	5	-0.538948	-0.480732
N(trans Bn)	12	-0.542090	-0.479353
O	2	-0.786228	-0.709546
O	6	-0.782235	-0.704545

Mulliken totals ***2**/SS*:

**Atom**	**Atom no**	**B3LYP-D3**	**BP86-D3**
Ga	1	1.547391	1.353508
F	3	-0.534844	-0.480406
N	4	-0.536811	-0.475752
N	5	-0.546728	-0.489225
N(trans Bn)	13	-0.544141	-0.480898
O	2	-0.779122	-0.702033
O	7	-0.786129	-0.707745

Mulliken totals ***1**/SS*:

**Atom**	**Atom no**	**B3LYP-D3**	**BP86-D3**
Ga	1	1.549725	1.355386
F	2	-0.515957	-0.467324
N	4	-0.522718	-0.461073
N	5	-0.531743	-0.472265
N(trans Bn)	12	-0.538608	-0.477502
O	3	-0.783798	-0.703302
O	6	-0.787026	-0.709414

Mulliken charges for ***1**/RR*

***1**/RR*	**B3LYP-D3**	**BP86-D3**
1Ga	1.491165	1.296008
2P	1.190567	1.084583
3P	1.200206	1.094949
4F	-0.527094	-0.4765
5O	-0.780415	-0.70253
6O	-0.802515	-0.7223
7O	-0.606471	-0.55455
8O	-0.600628	-0.54853
9N	-0.552810	-0.48984
10N	-0.541892	-0.47934
11H	-0.548677	-0.48883
12C	-0.094867	-0.14768
13H	0.125411	0.142598
14H	0.169927	0.188785
15C	-0.346002	-0.38375
16H	0.197915	0.218693
17H	0.148977	0.166554
18C	-0.135360	-0.20052
19H	0.183388	0.200373
20H	0.209972	0.230788
21C	-0.347063	-0.38526
22H	0.189946	0.20938
23H	0.148934	0.166338
24C	-0.060399	-0.10456
25H	0.145783	0.164706
26H	0.122955	0.139015

27C	-0.118784	-0.16865
28H	0.125783	0.143937
29H	0.196599	0.21163
30C	-0.067607	-0.11315
31H	0.163083	0.180037
32H	0.120121	0.136831
33C	-0.404814	-0.3957
34C	-0.396843	-0.38762
35C	-0.134641	-0.18457
36H	0.141064	0.160645
37H	0.198926	0.210702
38C	-0.060803	-0.1074
39H	0.159933	0.177051
40H	0.118702	0.135456
41C	-0.084289	-0.09446
42H	0.094410	0.108651
43C	-0.064808	-0.07422
44H	0.199687	0.214182
45C	-0.057355	-0.06522
46H	0.120438	0.128595
47C	-0.073836	-0.0829
48H	0.164971	0.17489
49C	-0.091452	-0.10476
50H	0.095666	0.105455
51C	-0.093979	-0.10737
52H	0.094755	0.104396
53C	-0.060261	-0.06802
54H	0.122958	0.131166
55C	-0.092280	-0.10554
56H	0.097969	0.108006
57C	-0.077162	-0.07394
58C	-0.081902	-0.09077
59H	0.096549	0.106077
60C	-0.080550	-0.08539
61H	0.120727	0.133781
62C	-0.080195	-0.08929
63H	0.096169	0.105544
64C	-0.105826	-0.11963
65H	0.095789	0.105221
66C	-0.084126	-0.09043
67H	0.099776	0.109707
68C	-0.100371	-0.11367
69H	0.096091	0.105561
70C	-0.091524	-0.10547
71H	0.102291	0.112033

Mulliken charges for ***2**/RS*

***1**/RR*	**B3LYP-D3**	**BP86-D3**
1 Ga	1.515325	1.322455
2 O	-0.775676	-0.698419
3 F	-0.543349	-0.486951
4 O	-0.602099	-0.550188
5 N	-0.549937	-0.489496
6 N	-0.550484	-0.492558
7 O	-0.600499	-0.549506
8 O	-0.778700	-0.700806

9 C	-0.099262	-0.151448
10 H	0.124370	0.141658
11 H	0.180556	0.199291
12 C	-0.079928	-0.083925
13 H	0.118407	0.131452
14 N	-0.548670	-0.485273
15 C	-0.138562	-0.189878
16 H	0.141536	0.161111
17 H	0.201117	0.212567
18 C	-0.085526	-0.084674
19 C	-0.058159	-0.103354
20 H	0.158758	0.175804
21 H	0.118725	0.135234
22 C	-0.344936	-0.382963
23 H	0.189816	0.208534
24 H	0.148204	0.165787
25 C	-0.086601	-0.097103
26 H	0.109808	0.124469
27 C	-0.135484	-0.197066
28 H	0.203206	0.223113
29 H	0.197209	0.217416
30 C	-0.093225	-0.107366
31 H	0.100552	0.110260
32 C	-0.083444	-0.089721
33 H	0.098799	0.108671
34 C	-0.044171	-0.089844
35 H	0.141861	0.159432
36 H	0.123327	0.140060
37 C	-0.092562	-0.105676
38 H	0.099467	0.109390
39 C	-0.063516	-0.107743
40 H	0.152471	0.171551
41 H	0.119306	0.135329
42 C	-0.118822	-0.166366
43 H	0.127864	0.146205
44 H	0.191156	0.205814
45 C	-0.359932	-0.397422
46 H	0.191655	0.211040
47 H	0.150638	0.168263
48 P	1.187888	1.080931
49 P	1.195668	1.090953
50 C	-0.061975	-0.070303
51 C	-0.103288	-0.117223
52 C	-0.080889	-0.089916
53 C	-0.092509	-0.105776
54 C	-0.059041	-0.067066
55 C	-0.400401	-0.390853
56 H	0.164259	0.173526
57 H	0.092534	0.102363
58 H	0.096595	0.106076
59 H	0.095952	0.105681
60 H	0.122386	0.130511
61 C	-0.057382	-0.065361
62 C	-0.092833	-0.105996
63 C	-0.080396	-0.089380
64 C	-0.105856	-0.119911
65 C	-0.065382	-0.072331

66 C	-0.401758	-0.391868
67 H	0.125452	0.133461
68 H	0.094940	0.104755
69 H	0.094995	0.104512
70 H	0.089504	0.099415
71 H	0.170949	0.176640

Table S6 Computed BP86-D3 Mulliken charges on the Ga atom and the six atoms bonded to Ga (Cl, N, N, $N_{(trans\ Bn)}$, O, O) for the isolated [GaCl(Bn-NODP)] Isomer ***1****/RR* (with no water).

Atom	Charge
Ga	1.151097
Cl	-0.456280
N	-0.481561
N	-0.478005
$N_{(trans\ Bn)}$	-0.467094
O	-0.711052
O	-0.687027

Table S7 Tables (14 in total) of computed hydrogen bond (HB) strengths for the 7 [GaF(Bn-NODP)] isomers listed in Table 2, at the B3LYP-D3 and BP86-D3 levels

Table for Hydrogen Bond strengths using ***1****/RR*/B3LYP-D3
MULTIWFN outputs—using calculated PEDs (Potential Energy Densities, V(r))

MultiWfn identifies 160 CPS.
System has 71 Atoms, 78 “formal bonds” and 160 CPs; i.e. we expect (160-71-78) = 11 “non-bonded interactions”

CP No.	V(r)/au	V(r) /kcal.mol^{-1}	Gaussian Atom numbering	Bond Type
86	-0.009747	-6.12	4F—14H	F--H
88	-0.008948	-5.61	4F—22H	F--H
107	-0.007581	-4.76	8O—29H	O--H
110	-0.011175	-7.01	4F—19H	F--H
112	-0.006553	-4.11	4F—48H	F--H
123	-0.008038	-5.04	6O—29H	O--H
137	-0.007393	-4.64	6O—16H	O--H
138	-0.006590	-4.14	5O—20H	O--H
141	-0.007512	-4.71	5O—37H	O--H
142	-0.005985	-3.76	6O—44H	O--H
145	-0.007607	-4.77	7O—37H	O—H

Table for Hydrogen Bond Strengths using ***1***/*RR*/BP86-D3
MULTIWFN outputs—using calculated PEDs (Potential Energy Densities, V(r))
MultiWfn identifies 161 CPS.
System has 71 Atoms, 78 "formal bonds" and 161 CPs; i.e. we expect (161-71-78) = 12 "non-bonded interactions"

CP No.	V(r)/au	V(r) /kcal.mol^{-1}	Gaussian Atom numbering	Bond Type
86	-0.005587	-3.50566	25H—41C	C—H
87	-0.010144	-6.36544	14H—4F	F—H
90	-0.009271	-5.81743	22H—4F	F—H
108	-0.007949	-4.98804	29H—8O	O—H
111	-0.011258	-7.06458	19H—4F	F—H
113	-0.006822	-4.28067	48H—4F	F—H
123	-0.008083	-5.0724	29H—6O	O—H
138	-0.007774	-4.87826	16H—6O	O—H
139	-0.006893	-4.32516	20H—5O	O—H
142	-0.007573	-4.7522	37H—5O	O—H
143	-0.006666	-4.18279	44H—6O	O—H
146	-0.007843	-4.92162	37H—7O	O—H

Table for Hydrogen Bond Strengths using ***2***/*RS*/B3LYP-D3

MULTIWFN outputs—using calculated PEDs (Potential Energy Densities, V(r))

MultiWfn identifies 159 CPS.
System has 71 Atoms, 78 "formal bonds" and 159 CPs; i.e. we expect (159-71-78) = 10 "non-bonded interactions"

CP No.	V(r)/au	V(r) /kcal.mol^{-1}	Gaussian Atom Numbering	Bond Type
77	-0.010553	-6.621976	44H—7O	O—H
102	-0.010900	-6.839705	46H—3F	F—H
106	-0.007188	-4.510252	29H—2O	O—H
115	-0.012191	-7.650140	71H—3F	F—H
120	-0.012741	-7.995022	11H—3F	F—H
133	-0.007474	-4.690145	25H—8O	O—H
134	-0.010898	-6.838679	23H—3F	F—H
139	-0.006784	-4.257204	56H—3F	F—H
141	-0.008131	-5.101997	17H—8O	O—H
143	-0.008434	-5.292250	17H—4O	O—H

Table for Hydrogen Bond Strengths using ***2***/*RS/*BP86-D3
MULTIWFN outputs—using calculated PEDs (Potential Energy Densities, V(r))

MultiWfn identifies 159 CPS.
System has 71 Atoms, 78 “formal bonds” and 159 CPs; i.e. we expect (159-71-78) = 10 “non-bonded interactions”

CP No.	V(r)/au	V(r) /kcal.mol^{-1}	Gaussian Atom numbering	Bond Type
77	-0.011068	-6.945111	44H—7O	O—H
101	-0.011015	-6.911830	46H—3F	O—F
105	-0.007551	-4.738471	29H—2O	O—H
113	-0.012690	-7.963251	71H—3F	O—F
119	-0.012948	-8.124978	11H—3F	O—F
133	-0.007732	-4.851881	28H—8O	O—H
134	-0.010981	-6.890538	23H—3F	O—F
139	-0.007063	-4.432018	56H—3F	O—F
142	-0.008810	-5.528575	17H—4O	O—H
159	-0.008091	-5.077047	17H—8O	O—H

Table for Hydrogen Bond Strengths using ***1***/*RS*/B3LYP-D3
MULTIWFN outputs—using calculated PEDs (Potential Energy Densities, V(r))

MultiWfn identifies 159 CPS.
System has 71 Atoms, 78 “formal bonds” and 159 CPs; i.e. we expect (159-71-78) = 10 “non-bonded interactions”

CP No.	V(r)/au	V(r) /kcal.mol^{-1}	Gaussian Atom numbering	Bond Type
96	-0.0106953	-6.71	42H—2F	F—H
103	-0.0059196	-3.71	61C—9H	C—H
111	-0.0076898	-4.83	9H—3O	O—H
121	-0.0121781	-7.64	27H—2F	F—H
134	-0.0069070	-4.33	3O—21H	O—H
136	-0.0020568	-1.29	66H—21H	H—H
139	-0.0074722	-4.69	15H—6O	O—H
142	-0.0063295	-3.97	26H—6O	O—H
143	-0.0071137	-4.46	15H—70O	O—H
144	-0.0062976	-3.95	3O—67H	O—H

Table for Hydrogen Bond Strengths using ***1***/*RS*/BP86-D3
MULTIWFN outputs—using calculated PEDs (Potential Energy Densities, V(r))

MultiWfn identifies 161 CPS.
System has 71 Atoms, 78 "formal bonds" and 161 CPs; i.e we expect (161-71-78) = 12 "non-bonded interactions"

CP No.	V(r)/au	V(r) /kcal.mol^{-1}	Gaussian Atom numbering	Bond Type
79	-0.0025516	-1.6011694	33H—60C	C—H
93	-0.0056719	-3.5591665	38H—23C	C—H
99	-0.0110664	-6.9442836	42H—2F	F—H
104	-0.0069207	-4.3427932	9H—61C	C—H
112	-0.0076114	-4.7762573	9H—3O	O—H
123	-0.0123308	-7.7376878	27H—2F	F—H
136	-0.0021998	-1.3804092	62H—21H	H—H
137	-0.0072643	-4.5584379	21H—3O	O—H
143	-0.0075745	-4.7530623	15H—6O	O—H
144	-0.0065834	-4.1311300	26H—6O	O—H
145	-0.0073000	-4.5808373	15H—70O	O—H
146	-0.0071171	-4.4660639	67H—3O	O—H

Table for Hydrogen Bond Strengths using ***1***/*SR*/B3LYP-D3
MULTIWFN outputs—using calculated PEDs (Potential Energy Densities, V(r))

MultiWfn identifies 161 CPS.
System has 71 Atoms, 78 "formal bonds" and 161 CPs; i.e. we expect (161-71-78) = 12 "non-bonded interactions"

CP No.	V(r)/au	V(r) /kcal.mol^{-1}	Gaussian Atom numbering	Bond Type
83	-0.002520	-1.581508	18H—61C	C—H
86	-0.007200	-4.517922	21H—3O	O—H
87	-0.007804	-4.897162	9H—59O	O—H
92	-0.008459	-5.307882	9H—3O	O—H
100	-0.006347	-3.982582	15H—60C	C—H
113	-0.007310	-4.587219	15H—6O	O—H
122	-0.002177	-1.366271	11H—65C	C—H
125	-0.006257	-3.926202	26H—6O	O—H
134	-0.005764	-3.617275	56H—2F	F—H
136	-0.008475	-5.317874	45H—2F	F—H
145	-0.009401	-5.899187	42H—2F	F—H
146	-0.011460	-7.191110	27H—2F	F—H

Table for Hydrogen Bond Strengths using ***1***/*SR*/BP86-D3
MULTIWFN outputs—using calculated PEDs (Potential Energy Densities, V(r))

MultiWfn identifies 161 CPS.
System has 71 Atoms, 78 “formal bonds” and 161 CPs; i.e. we expect (161-71-78) = 12 “non-bonded interactions”

CP No.	V(r)/au	V(r) / kcal mol^{-1}	Gaussian Atom numbering	Bond Type
83	-0.002761	-1.73	61C—18H	C—H
85	-0.007458	-4.68	21H—3O	O—H
87	-0.008153	-5.12	59O—9H	O—H
93	-0.008540	-5.36	3O—9H	O—H
100	-0.007425	-4.66	60C—15H	C—H
113	-0.007270	-4.56	6O—15H	O—H
122	-0.002514	-1.58	65C—11H	C—H
124	-0.006606	-4.15	26H—6O	O—H
134	-0.006033	-3.79	56H—2F	F—H
136	-0.008747	-5.49	2F—45H	F—H
145	-0.009796	-6.15	2F—42H	F—H
146	-0.011483	-7.21	2F—27H	F—H

Table for Hydrogen Bond strengths using ***2***/*RR*/B3LYP-D3
MULTIWFN outputs—using calculated PEDs (Potential Energy Densities, V(r))

MultiWfn identifies 160 CPS.
System has 71 Atoms, 78 “formal bonds” and 160 CPs; i.e. we expect (160-71-78) = 11 “non-bonded interactions”

CP No.	V(r)/au	V(r) /kcal mol^{-1}	Gaussian Atom numbering	Bond Type
81	-0.010692	-6.71	44H—3F	F—H
87	-0.013162	-8.26	9H—3F	F—H
91	-0.007472	-4.69	64C—42H	C—H
96	-0.006731	-4.22	3F—54H	F—H
103	-0.011042	-6.93	3F—21H	F—H
118	-0.007301	-4.58	2O—27H	O—H
121	-0.003154	-1.98	65H—27H	H—H
123	-0.000796	-0.50	59C—24H	C—H
139	-0.007615	-4.78	6O—26H	O—H
141	-0.008281	-5.20	6O—15H	O—H
150	-0.008272	-5.19	15H—70O	O—H

Test Case Table for Hydrogen bond Strengths using ***2****/RR/*BP86-D3
MULTIWFN outputs—using calculated PEDs (Potential Energy Densities, V(r))

MultiWfn identifies 160 CPS.
System has 71 Atoms, 78 "formal bonds" and 160 CPs; i.e. we expect (160-71-78) = 11 "non-bonded interactions"

CP No.	V(r)/au	V(r) /kcal.mol^{-1}	Gaussian Atom numbering	Bond Type
82	-0.00857909	-5.38	15H—70O	O—H
101	-0.00776257	-4.87	26H—6O	O—H
119	-0.001132560	-0.71	24H—59C	C—H
123	-0.007920299	-4.97	27H—2O	O—H
126	-0.011096526	-6.96	21H—3F	F—H
128	-0.003398407	-2.13	65H—27H	H—H
131	-0.013443700	-8.44	9H—3F	F—H
138	-0.006929221	-4.35	54H—3F	F—H
139	-0.008565792	-5.38	42H—64C	C—H
143	-0.010810911	-6.78	44H—3F	F—H
160	-0.008262787	-5.18	15H—6O	O—H

Table for Hydrogen Bond strengths using ***2****/SS/*B3LYP-D3
MULTIWFN outputs—using calculated PEDs (Potential Energy Densities, V(r))

MultiWfn identifies 160 CPS.
System has 71 Atoms, 78 "formal bonds" and 160 CPs; i.e. we expect (160-71-78) = 11 "non-bonded interactions"

CP No.	V(r)/au	V(r) /kcal.mol^{-1}	Gaussian Atom numbering	Bond Type
87	-0.010597	-6.649986	43H—6O	O—H
96	-0.007405	-4.646751	28H—2O	O—H
112	-0.002053	-1.288476	12H—50O	O—H
117	-0.007183	-4.507311	27H—7O	O—H
122	-0.008032	-5.040415	16H—7O	O—H
126	-0.006643	-4.168489	16H—54C	C—H
128	-0.011717	-7.352777	45H—3F	F—H
138	-0.013680	-8.584193	10H—3F	F—H
143	-0.011554	-7.250100	70H—3F	F—H
146	-0.010465	-6.566982	22H—3F	F—H
155	-0.004068	-2.552954	23H—59H	H—H

Table for Hydrogen Bond Strengths using ***2***/*SS*/BP86-D3
MULTIWFN outputs—using calculated PEDs (Potential Energy Densities, V(r))

MultiWfn identifies 161 CPS.
System has 71 Atoms, 78 “formal bonds” and 161 CPs; i.e. we expect (161-71-78) = 12 “non-bonded interactions”

CP No.	V(r)/au	V(r) /kcal.mol^{-1}	Gaussian Atom numbering	Bond Type
87	-0.0111517	-7.00	43H—6O	O—H
97	-0.0077040	-4.83	28H—2O	O—H
112	-0.0024021	-1.51	12H—50C	C—H
118	-0.0075128	-4.71	27H—7O	O—H
122	-0.0078520	-4.93	16H—7O	O—H
127	-0.0078613	-4.93	16H—54C	C—H
128	-0.0118409	-7.43	45H—3F	F—H
134	-0.0024381	-1.53	19H—53C	C—H
140	-0.0139588	-8.76	10H—3F	F—H
144	-0.0119885	-7.52	70H—3F	F—H
150	-0.0105646	-6.63	22H—3F	F—H
156	-0.0040906	-2.57	59H—23H	H—H

Table for Hydrogen Bond strengths using ***1***/*SS*/B3LYP-D3
MULTIWFN outputs—using calculated PEDs (Potential Energy Densities, V(r))

MultiWfn identifies 161 CPS.
System has 71 Atoms, 78 “formal bonds” and 161 CPs; i.e. we expect (161-71-78) = 11 “non-bonded interactions”

CP No.	V(r)/au	V(r) /kcal.mol^{-1}	Gaussian Atom numbering	Bond Type
86	-0.010594	-6.64782	43H—2F	H—F
98	-0.012653	-7.94019	27H—2F	H—F
122	-0.005992	-3.76019	26H—6O	O—H
126	-0.007973	-5.00343	9H—3O	O—H
128	-0.005957	-3.73807	9H—62C	C—H
130	-0.007114	-4.46426	15H—6O	O—H
132	-0.001757	-1.10223	26H—68H	H—H
133	-0.002075	-1.30212	11H—52C	C—H
135	-0.006130	-3.84654	15H—48C	C—H
138	-0.002488	-1.56143	18H—48C	C—H
141	-0.001811	-1.13651	21H—67H	H—H

Table for Hydrogen Bond strengths using ***1****/SS*/BP86-D3
MULTIWFN outputs—using calculated PEDs (Potential Energy Densities, V(r))

MultiWfn identifies 161 CPS.
System has 71 Atoms, 78 "formal bonds" and 161 CPs; i.e. we expect (161-71-78) = 12 "non-bonded interactions"

CP No.	V(r)/au	V(r) /kcal.mol^{-1}	Gaussian Atom numbering	Bond Type
86	-0.011046	-6.93	43H—2F	F—H
98	-0.012628	-7.92	27H—2F	F—H
101	-0.002591	-1.63	33H—57C	C—H
123	-0.006345	-3.98	6O—26H	O—H
127	-0.008032	-5.04	9H—3O	O—H
129	-0.006939	-4.35	62C—9H	C—H
131	-0.007083	-4.44	15H—6O	O—H
133	-0.002433	-1.53	11H—52C	C—H
134	-0.001847	-1.16	26H—68H	H—H
136	-0.007240	-4.54	15H—48C	C—H
139	-0.002696	-1.69	18H—49C	C—H
142	-0.001894	-1.19	21H—67H	H—H

Table S8 AIM results obtained for [GaCl(BN-NODP)] ***Isomer 1****/RR* at the B3LYP-D3 and BP86-D3 levels

Isomer 1*/RR* [GaCl(BN-NODP)] B3LYP-D3 results:

Table for Hydrogen Bond strengths using Ga-Cl ***1****/RR*/B3LYP-D3

MULTIWFN outputs—using calculated PEDs (Potential Energy Densities, V(r))

MultiWfn identifies 161 CPs.

System has 71 Atoms, 78 "formal bonds" and 161 CPs; i.e. we expect (161-71-78) = 12 "non-bonded interactions"

CP No.	V(r)/au	V(r) /kcal.mol^{-1}	Gaussian Atom numbering	Bond Type
85	-0.005029	-3.15557	38H—23C	C—H
88	-0.005899	-3.70137	42H—2Cl	Cl—H
89	-0.005973	-3.74805	45H—2Cl	Cl—H
107	-0.010356	-6.49833	9H—71O	O—H
108	-0.007954	-4.99133	27H—2Cl	Cl—H
120	-0.004775	-2.99651	66H—2Cl	Cl—H
137	-0.005811	-3.64629	26H—6O	O—H
138	-0.007163	-4.4946	21H—6O	O—H
141	-0.007622	-4.78316	15H—6O	O—H
143	-0.005781	-3.62739	67H—3O	O—H
145	-0.007426	-4.6600	15H—70O	O—H
161	-0.007706	-4.83583	9H—3O	O—H

Isomer 1*/RR* GaCl(BN-NODP) BP86-D3 results

Table for Hydrogen Bond strengths using Ga-Cl ***1***/*RR*/BP86-D3

MULTIWFN outputs—using calculated PEDs (Potential Energy Densities, V(r))

MultiWfn identifies 160 CPs.

System has 71 Atoms, 78 “formal bonds” and 160 CPs; i.e. we expect (160-71-78) = 11 “non-bonded interactions”

CP No.	V(r)/au	V(r) /kcal.mol^{-1}	Gaussian Atom numbering	Bond Type
85	-0.005273	-3.30892	38H—23C	C—H
88	-0.006228	-3.907848	42H—2Cl	Cl—H
91	-0.006331	-3.97259	45H—2Cl	Cl—H
106	-0.010636	-6.67429	71O—9H	O—H
109	-0.008224	-5.16069	27H—2Cl	Cl—H
120	-0.005064	-3.17784	66H—2Cl	Cl—H
137	-0.007561	-4.74429	21H—3O	O—H
138	-0.006072	-3.81006	26H—6O	O—H
141	-0.007739	-4.85653	15H—6O	O—H
143	-0.006460	-4.05354	67H—3O	O—H
145	-0.007627	-4.78609	15H—70O	O—H

Table S9

(a) Mulliken atomic charge densities obtained by taking the geometry of [GaF(Bn-NODP)] ***isomer1/RR*** optimized at the BP86-D3 level and carrying out fixed point calculations of $(Ga\text{-}F)^0$ and $(Ga\text{-}F)^{2+}$ and the $ligand^0$ and $ligand^{2-}$. Comparison of Tables S9 (a) and (b) shows that [GaF(Bn-NODP)] is closer to $(Ga\text{-}F)^{2+}$ $ligand^{2-}$ than $(Ga\text{-}F)^0(ligand)^0$.

	**BP86-D3**	
**Mulliken charge**	**Ga-F**	**$(Ga\text{-}F)^{2+}$**
**Ga**	0.446889	1.973077
**F**	-0.446889	0.026923
**Total charge**	0.000	2.000

	**BP86-D3**	
**Mulliken charge**	**Ligand**	**$Ligand^{2-}$**
**N (N8)**	-0.3588	-0.2125
**N (N9)**	-0.2971	-0.2308
**$N_{(trans\ Bn)}$ (N7)**	-0.1147	-0.1814
**O (O3)**	-0.7482	-0.6168
**O (O4)**	-0.7792	-0.5835
**Total charge of the 5 atoms bonded to Ga (apart from F)**	-2.298	-1.825

(b) Computed BP86-D3 Mulliken charges on the Ga atom and the six atoms bonded to Ga (F, N, N, $N_{(trans\ Bn)}$, O, O) for case (d) listed in the text for [GaF(Bn-NODP)] ***isomer 1****/RR*

**Atom**	**GaF(Bn-NODP) *isomer 1/RR* case (d) optimized**
**Ga**	1.2960
**F**	-0.4765
**Total change on Ga-F unit**	+0.8195
**N**	-0.4793
**N**	-0.4888
**$N_{(trans\ Bn)}$**	-0.4898
**O**	-0.7025
**O**	-0.7223
**Total charge of the 5 atoms bonded to Ga (apart from F)**	-2.8827

***isomer 1**/RR [GaF(Bn-NODP)]*	
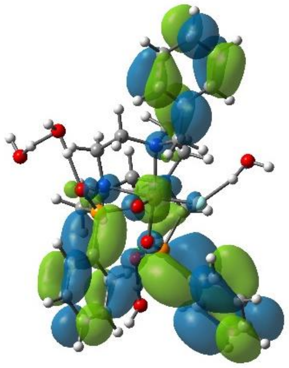	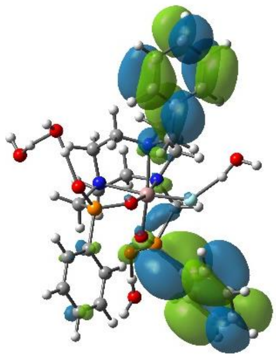
LUMO	LUMO +1
-1.75 eV	-1.65 eV
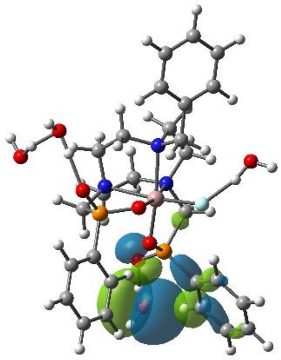	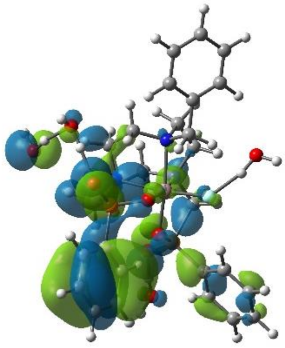
HOMO	HOMO -1
-5.78 eV	-6.24 eV

Figure S11: Representations of the HOMO – 1, HOMO, LUMO, and LUMO + 1 of ***isomer 1**/RR* [GaF(Bn-NODP)]·4H_2O determined by BP86-D3 calculations.

isomer 1/*RR [GaF(Bn-NODP)]*	
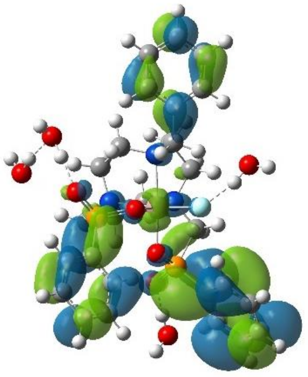	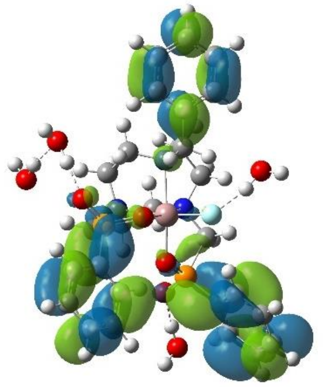
LUMO	LUMO +1
-0.87 eV	-0.77 eV
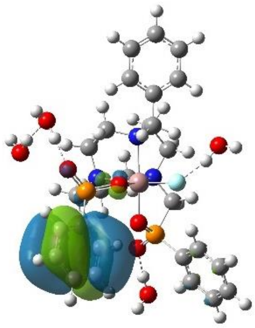	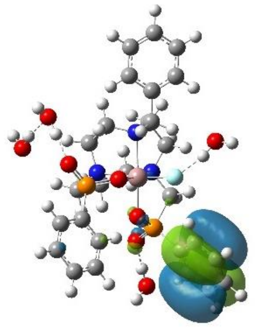
HOMO	HOMO -1
-6.95 eV	-6.98 eV

Figure S12: Representations of the HOMO – 1, HOMO, LUMO, and LUMO + 1 of ***isomer 1***/*RR* [GaF(Bn-NODP)]·4H_2O determined by B3LYP-D3 calculations.

***isomer 1**/RR [GaF(Bn-NODP)]*	
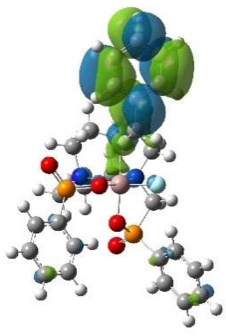	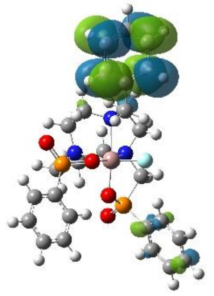
LUMO	LUMO +1
-1.77 eV	-1.66 eV
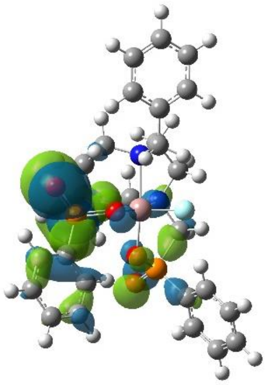	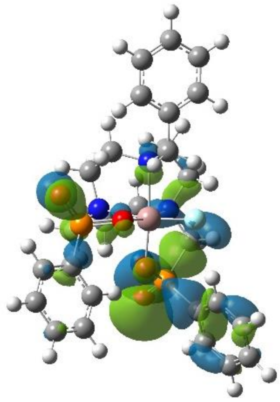
HOMO	HOMO -1
-5.91 eV	-5.97 eV

Figure S13: Representations of the HOMO – 1, HOMO, LUMO, and LUMO + 1 of ***isomer 1**/RR* [GaF(Bn-NODP)] determined by BP86-D3 calculations.

isomer 1*/RR [GaF(Bn-NODP)]*	
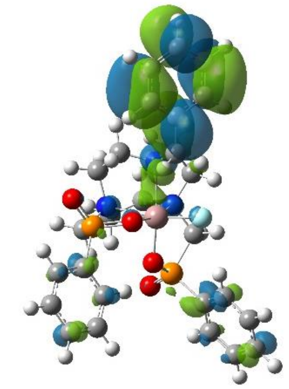	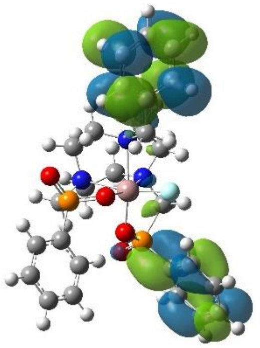
LUMO	LUMO +1
-0.87 eV	-0.75 eV
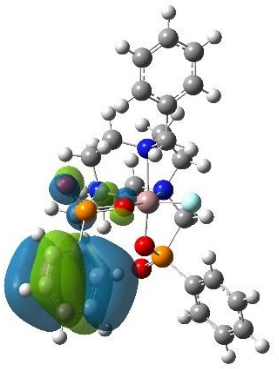	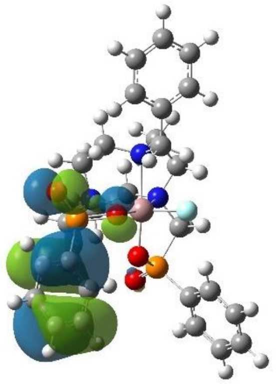
HOMO	HOMO -1
-6.82 eV	-6.87 eV

Figure S14: Representations of the HOMO – 1, HOMO, LUMO, and LUMO + 1 of ***isomer 1/****RR* [GaF(Bn-NODP)] determined by B3LYP-D3 calculations.

***isomer 2**/RS [GaF(Bn-NODP)]*	
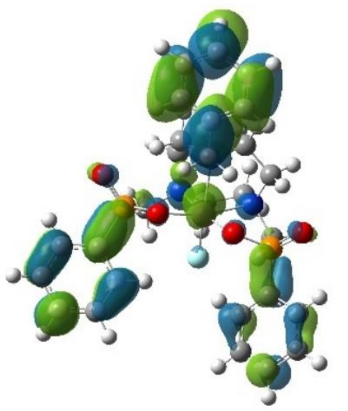	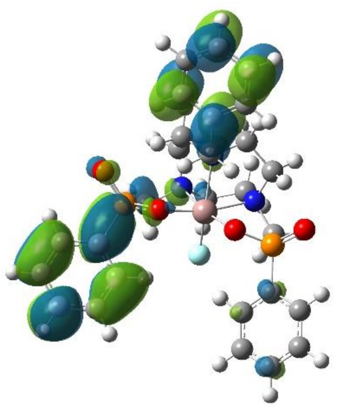
LUMO	LUMO +1
-1.69 eV	-1.59 eV
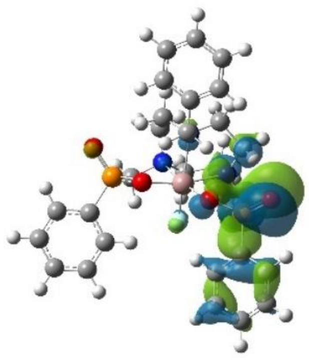	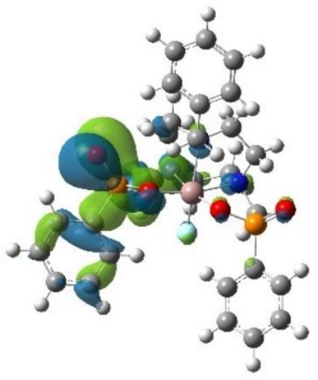
HOMO	HOMO -1
-5.90 eV	-5.99 eV

Figure S15: Representations of the HOMO – 1, HOMO, LUMO, and LUMO + 1 of ***isomer 2**/RS* [GaF(Bn-NODP)] determined by BP86-D3 calculations.

***isomer 2**/RS [GaF(Bn-NODP)]*	
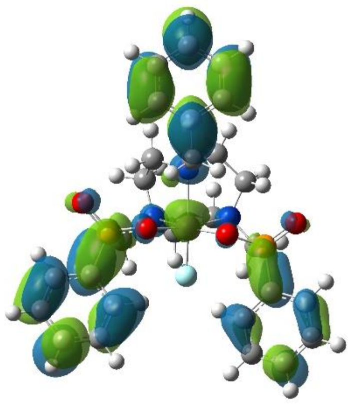	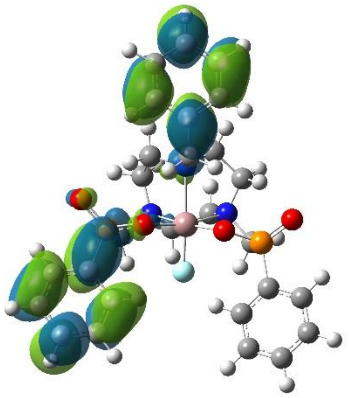
LUMO	LUMO +1
-0.81 eV	-0.71 eV
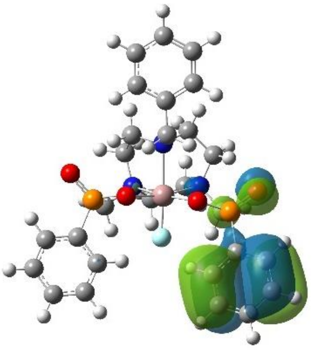	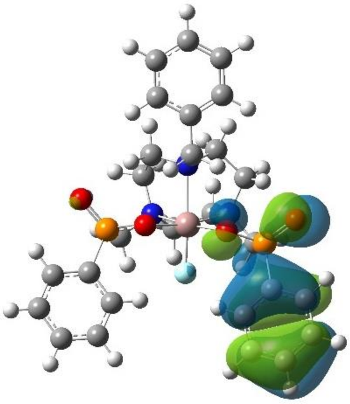
HOMO	HOMO -1
-6.84 eV	-6.87 eV

Figure S16: Representations of the HOMO – 1, HOMO, LUMO, and LUMO + 1 of ***isomer 2**/RS* [GaF(Bn-NODP)] determined by B3LYP-D3 calculations.

Crystal structure of ***isomer 1***/*RR* [GaF(Bn-NODP)]·$4H_2O$	Crystal structure of ***isomer 1***/*RR* [GaF(Bn-NODP)]
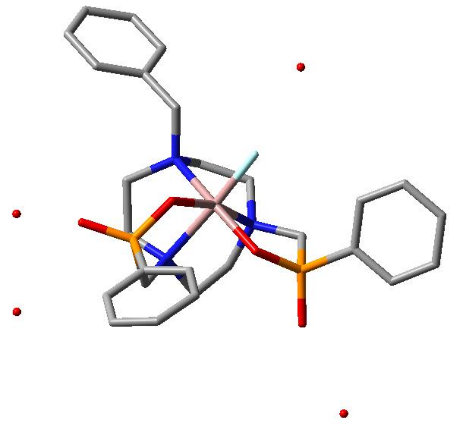	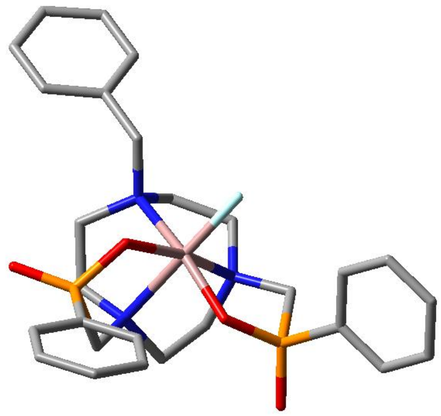
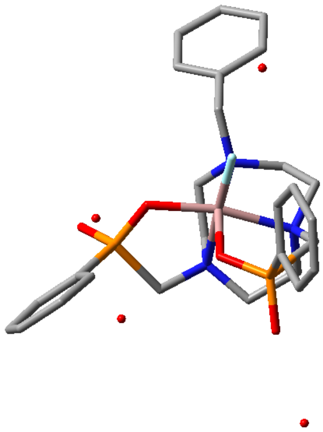	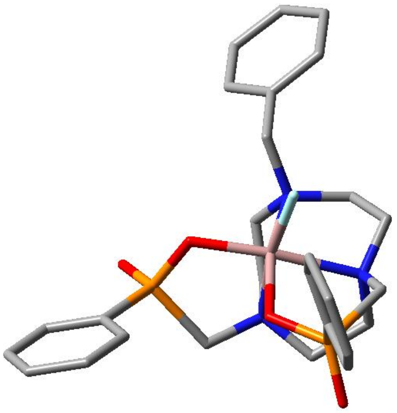
Optimized – with water	Optimized - no water
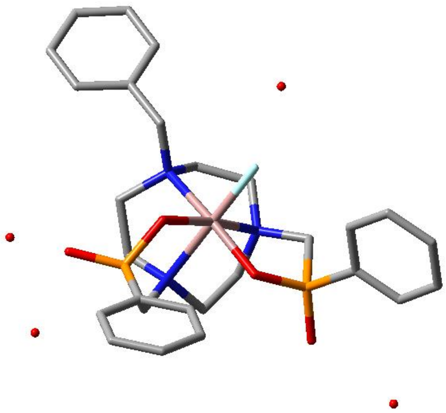	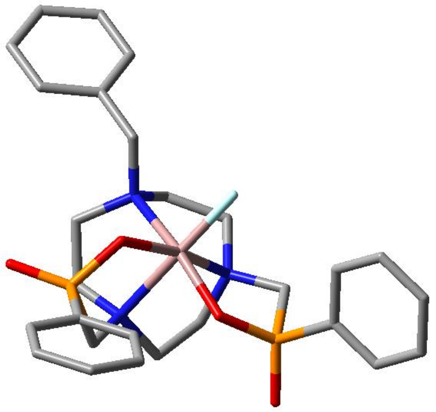

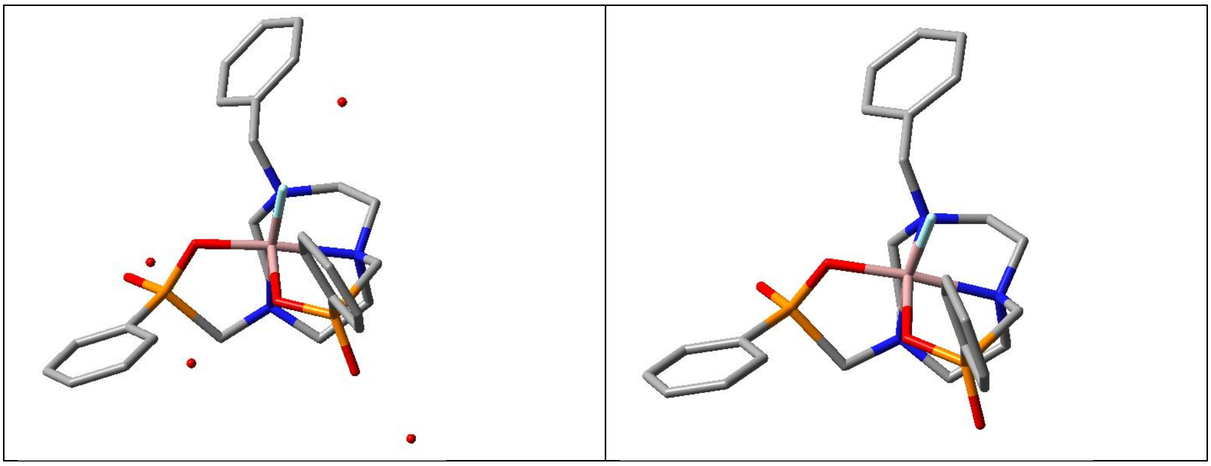

Figure S17 Alternative views of the structures of [GaF(Bn-NODP)] (***isomer 1***/*RR*) used in the calculations described in the text (a)-(d) of the manuscript showing the structures obtained from the X-ray data (a) and (c) and the optimization calculations (b) and (d) both with and without water ([a] BP86-D3 functional); the labels (a) – (d) follow those in the text of the manuscript.

Table S10 Comparison of the computed bond lengths and angles for the [GaF(Bn-NODP)] ***isomer 1***/*RR*, at the B3LYP-D3 and BP86-D3 levels with experimental values from X-ray diffraction.

Bond Lengths (Å):

[GaF(Bn-NODP)]		Optimized with 4 water molecules		Optimized with no water	
Atom numbers (from X-ray)	X-ray	B3LYP-D3	BP86-D3	B3LYP-D3	BP86-D3
Ga1-F1	1.832(1)	1.867990	1.88617	1.84404	1.86061
Ga1-O2	1.936(1)	1.898268	1.91391	1.89184	1.90662
Ga1-O1	1.940(2)	1.939172	1.95327	1.92736	1.94187
Ga1-N1	2.141(2)	2.178686	2.17824	2.19888	2.19865
Ga1-N2	2.150(2)	2.187681	2.18495	2.22452	2.22521
Ga1-N3	2.153(2)	2.176042	2.17324	2.20222	2.20013
P1-O1	1.536(2)	1.565671	1.58137	1.57082	1.58700
P1-O3	1.493(2)	1.504601	1.51961	1.49690	1.51153
P2-O2	1.536(1)	1.566928	1.58196	1.57463	1.59096
P2-O4	1.496(2)	1.507530	1.52359	1.49701	1.51167

Bond Angles (°):

[GaF(Bn-NODP)]		Optimized with 4 water molecules		Optimized with no water	
Atom numbers (from X-ray)	X-ray	B3LYP-D3	BP86-D3	B3LYP-D3	BP86-D3
O1-Ga1-N1	86.00(7)	85.02812	85.76643	85.20643	86.04083
O1-Ga1-N2	93.69(7)	89.68799	89.34606	88.68411	88.34250
O2-Ga1-N2	85.40(6)	86.81614	87.70819	85.97785	86.87735
O2-Ga1-N3	98.92(6)	93.26792	92.89707	91.89960	91.46438
O1-P1-C10	102.8(1)	101.20188	101.55684	100.92460	101.26590
O2-P2-C19	102.73(9)	102.53498	103.21099	101.17605	101.68515
F1-Ga1-N1	95.85(6)	90.33238	90.53536	88.32602	88.12881
F1-Ga1-N2	173.10(6)	170.04822	170.78205	165.86117	165.95826
F1-Ga1-N3	90.38(6)	92.07573	92.61796	90.42106	90.68875
F1-Ga1-O1	92.27(6)	94.88982	94.72664	98.06078	98.26132

F1-Ga1-O2	97.84(6)	101.25561	99.92421	105.03536	104.20991

Radiochemistry

Table S11: Radiochemical purity (RCP) as a function of time in different formulations (at 1 mg of precursor per mL) for [M^{18}F(Bn-NODP)] (M = Ga, Fe)

[Ga^{18}F(Bn-NODP)]	% RCP	
Time / h	10 : 90 (EtOH/H_2O)	10 : 90 (EtOH/PBS)
0	100	100
1	96	95
2.5	94	94
3.5	94	95

[Fe^{18}F(Bn-NODP)]	% RCP	
Time (h)	10 : 90 (EtOH/H_2O)	10 : 90 (EtOH/PBS)
0	92	88
1	90	2
2	74	0
3	48	0
4	35	0

HPLC radiotraces from [GaCl(Bn-NODP)] + $^{18}F^{-}$ purified by SPE HLB cartridge at 1 mg/mL.

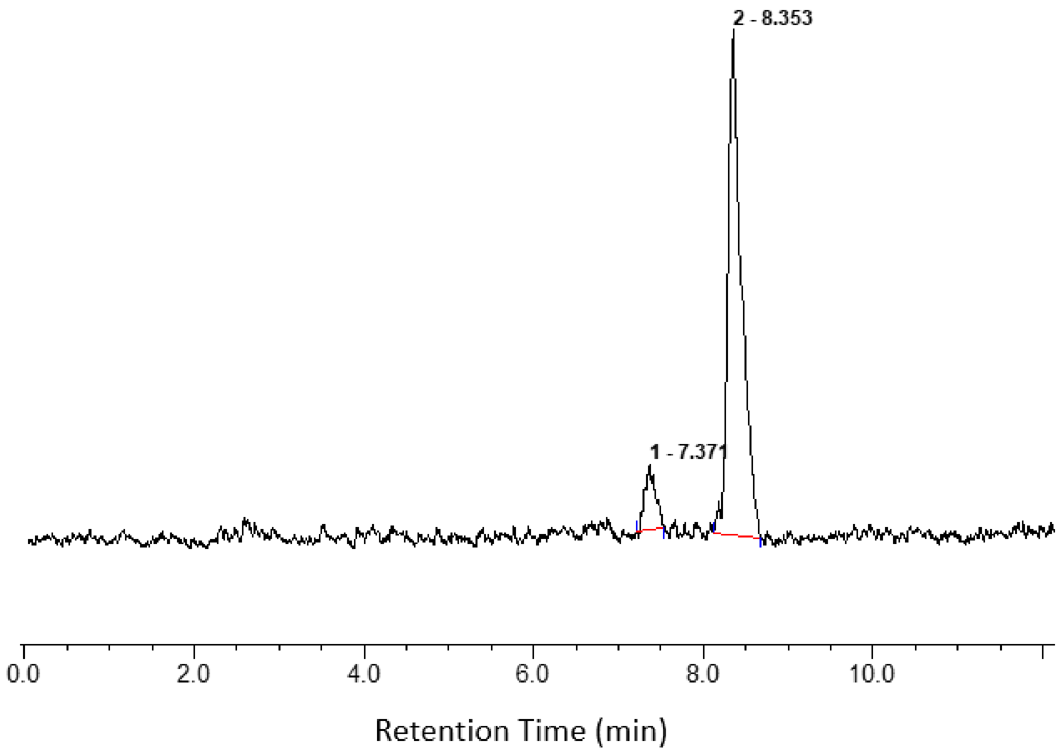

Figure S18 HPLC radiotrace from [GaCl(Bn-NODP)] + $^{18}F^{-}$ 1 mg/mL SPE purified formulated in 10:90 EtOH:1 M NaOAc Buffer t = 0 h.

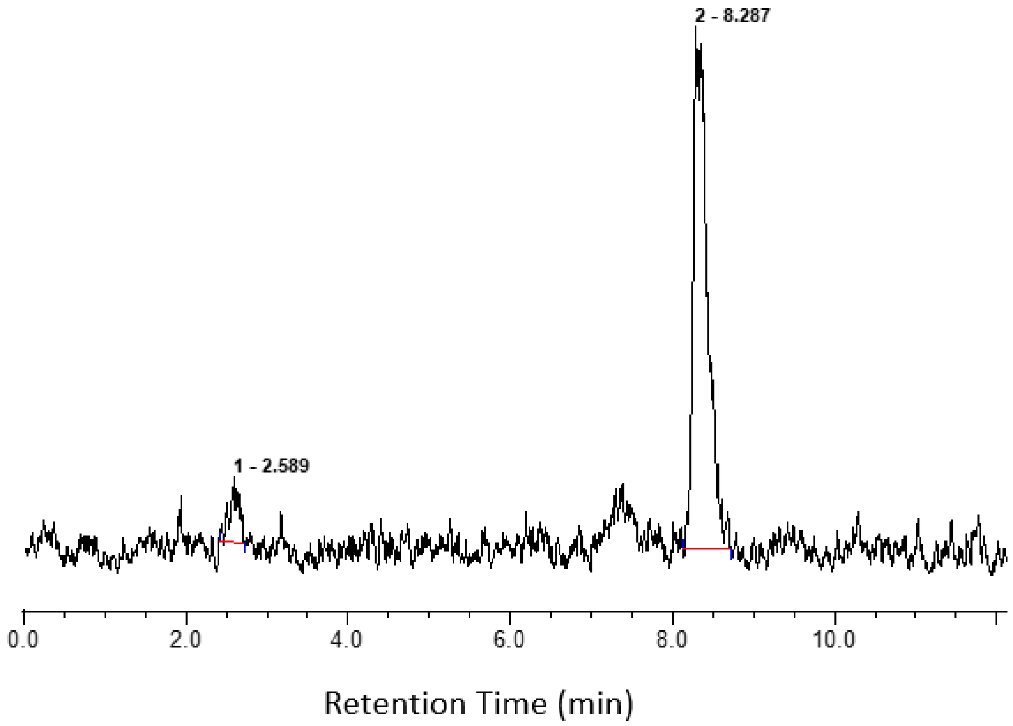

Figure S19 HPLC radiotrace from [GaCl(Bn-NODP)] + $^{18}F^{-}$ 1 mg/mL SPE purified formulated in 10:90 EtOH:1 M NaOAc Buffer t = 4 h.

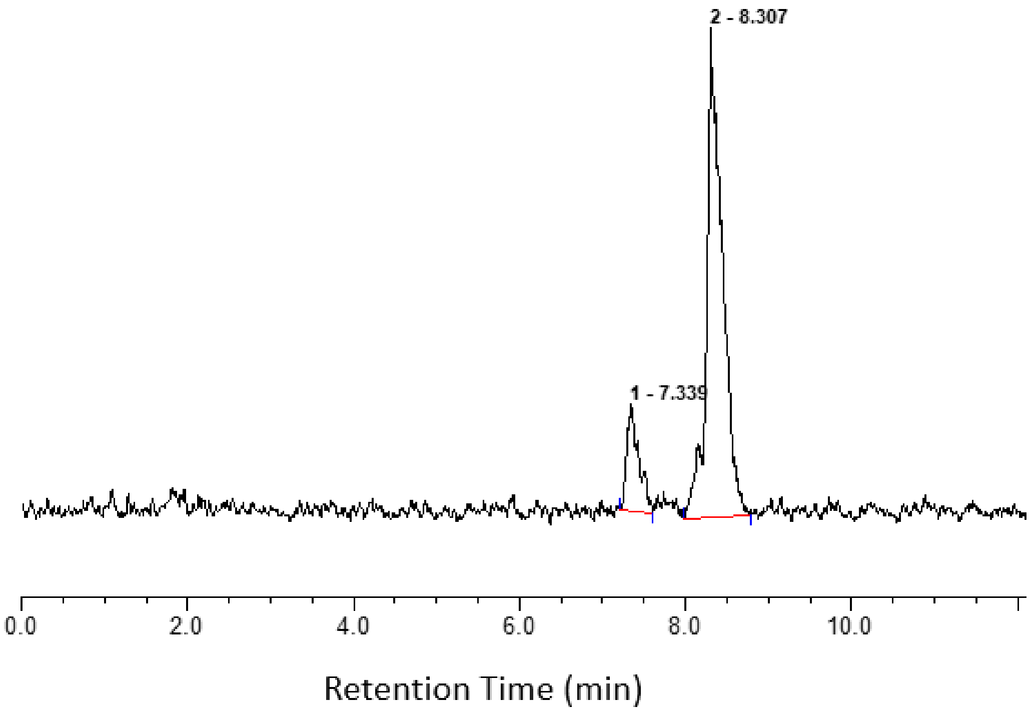

Figure S20 HPLC radiotrace from [GaCl(Bn-NODP)] + $^{18}F^{-}$ 1 mg/mL SPE purified formulated in 10:90 EtOH:Water t = 0

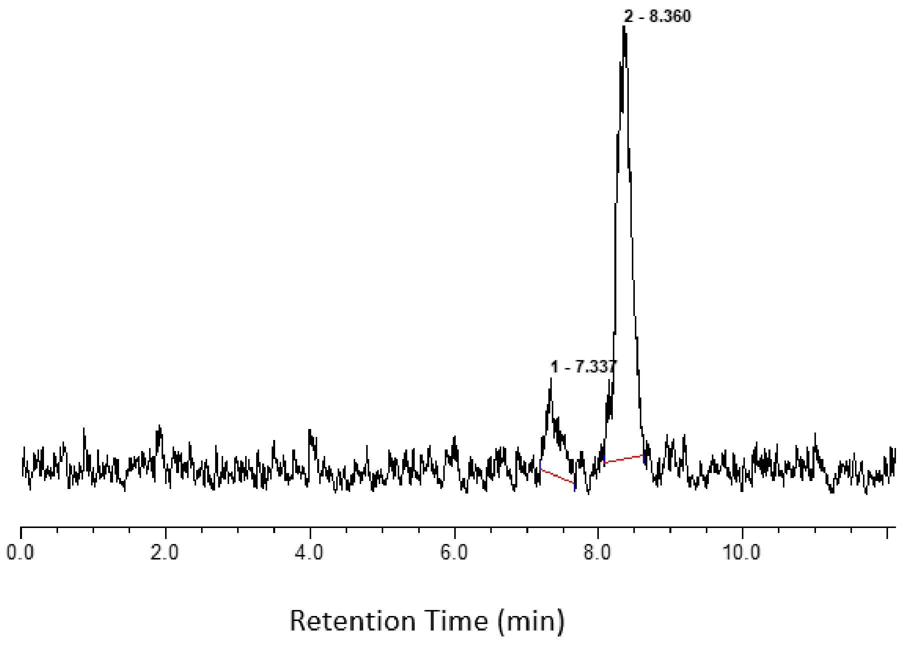

Figure S21 HPLC radiotrace from [GaCl(Bn-NODP)] + $^{18}F^{-}$ 1 mg/mL SPE purified formulated in 10:90 EtOH:Water t = 4 h.

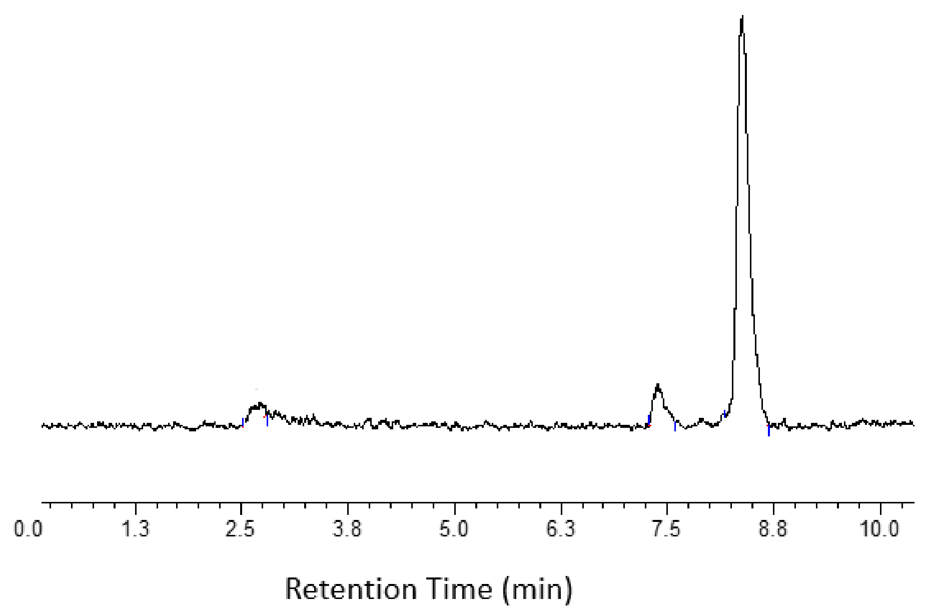

Figure S22 HPLC radiotrace from [GaCl(Bn-NODP)] + $^{18}F^{-}$ 1 mg/mL SPE purified formulated in 10:90 EtOH:PBS t = 0.

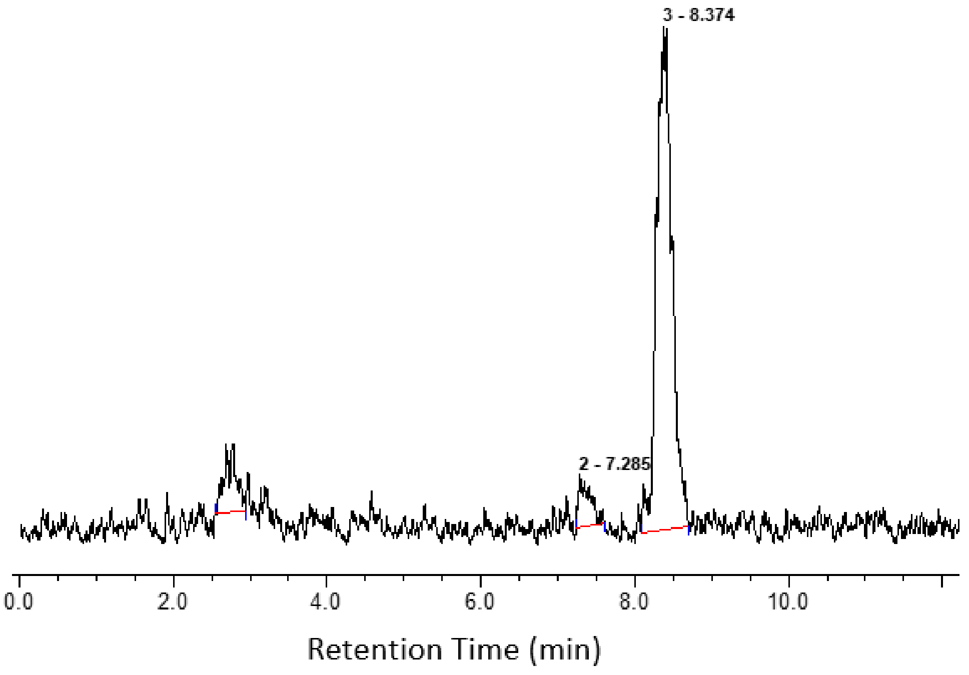

Figure S23 HPLC radiotrace from [GaCl(Bn-NODP)] + $^{18}F^{-}$ 1 mg/mL SPE purified formulated in 10:90 EtOH:PBS t = 4 h.

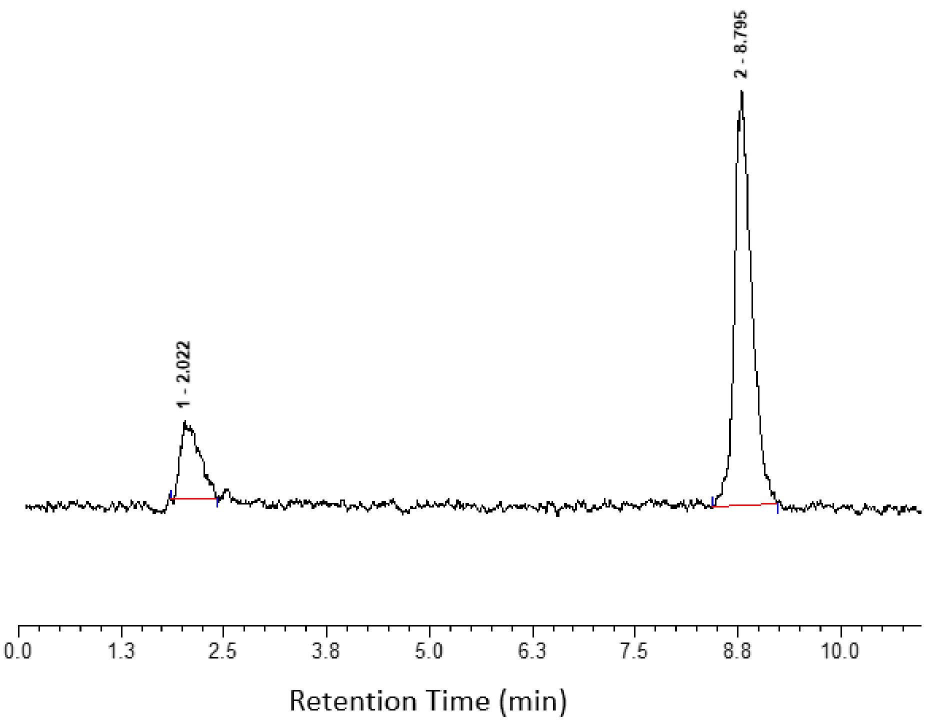

Figure S24 HPLC radiotrace from [FeCl(Bn-NODP)] + $^{18}F^{-}$ 1 mg/mL; crude

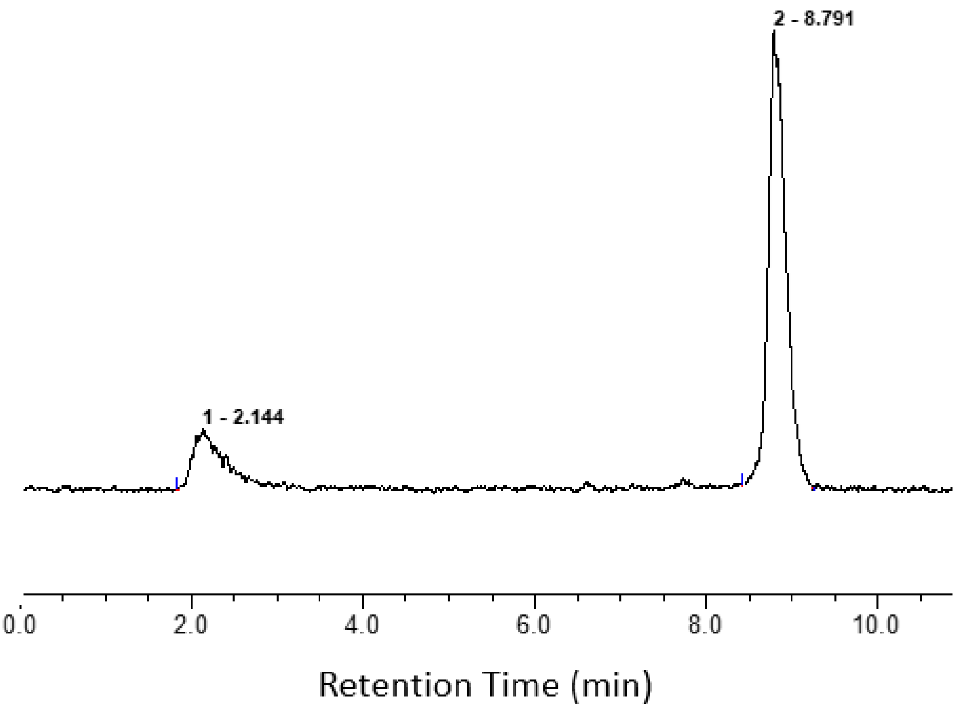

Figure S25 HPLC radiotrace from [Fe^{18}F(Bn-NODP)] + $^{18}F^{-}$ 0.1 mg/mL; crude

HPLC radiotraces from [FeCl(Bn-NODP)] purified by SPE HLB cartridge at 1 mg/mL.

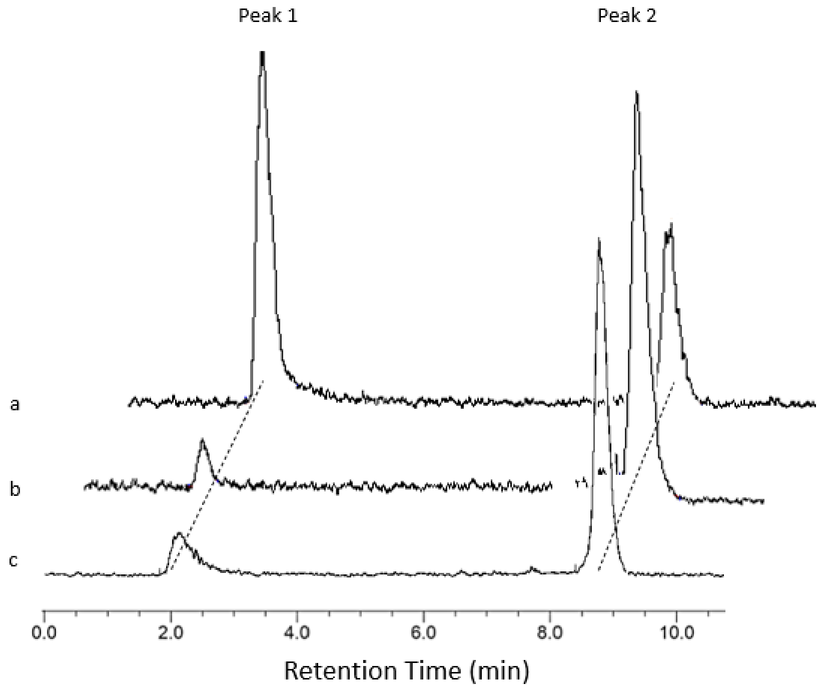

Figure S26 a) Analytical radio-HPLC of the crude product. Peak 1: R_t = 2.65 min 12.6 % ([^{18}F]F$^-$], peak 2: R_t = 8.79 min 87.4 % ([Fe^{18}F(Bn-NODP)]); b) analytical radio-HPLC trace of the purified product eluted from an HLB cartridge (formulated in 10% EtOH/ 90% Water); Peak 1: R_t = 1.9 min 6.1 % ([^{18}F]F$^-$], peak 2: R_t = 8.77 min 93.9 % ([Fe^{18}F(Bn-NODP)]); c) analytical radio-HPLC trace of the purified product eluted from an HLB cartridge (formulated in 10% EtOH/ 90% Water after 240 mins; Peak 1: R_t = 2.30 min 64.9 % ([^{18}F]F$^-$], peak 2: R_t = 8.82 min 35.1 % ([Fe^{18}F(Bn-NODP)]).
